# Genome-wide association study of long COVID

**DOI:** 10.1038/s41588-025-02100-w

**Published:** 2025-05-21

**Authors:** Vilma Lammi, Tomoko Nakanishi, Samuel E. Jones, Shea J. Andrews, Juha Karjalainen, Beatriz Cortés, Heath E. O’Brien, Ana Ochoa-Guzman, Brian E. Fulton-Howard, Martin Broberg, Hele H. Haapaniemi, Masahiro Kanai, Matti Pirinen, Axel Schmidt, Ruth E. Mitchell, Abdou Mousas, Massimo Mangino, Alicia Huerta-Chagoya, Nasa Sinnott-Armstrong, Elizabeth T. Cirulli, Marc Vaudel, Alex S. F. Kwong, Amit K. Maiti, Minttu M. Marttila, Daniel C. Posner, Alexis A. Rodriguez, Chiara Batini, Francesca Minnai, Anna R. Dearman, C. A. Robert Warmerdam, Celia B. Sequeros, Thomas W. Winkler, Daniel M. Jordan, Raimonds Rešcenko, Lorenzo Miano, Jacqueline M. Lane, Ryan K. Chung, Beatriz Guillen-Guio, Olivia C. Leavy, Laura Carvajal-Silva, Kevin Aguilar-Valdés, Erika Frangione, Lindsay Guare, Ekaterina Vergasova, Eirini Marouli, Pasquale Striano, Ummu Afeera Zainulabid, Ashutosh Kumar, Hajar Fauzan Ahmad, Ryuya Edahiro, Shuhei Azekawa, Long COVID Host Genetics Initiative, Vilma Lammi, Tomoko Nakanishi, Samuel E. Jones, Shea J. Andrews, Juha Karjalainen, Brian E. Fulton-Howard, Amit K. Maiti, Minttu M. Marttila, Eirini Marouli, Pasquale Striano, Ummu Afeera Zainulabid, Ashutosh Kumar, Hajar Fauzan Ahmad, Hugo Zeberg, Hanna M. Ollila, FinnGen, Vilma Lammi, Samuel E. Jones, Martin Broberg, Hele H. Haapaniemi, Masahiro Kanai, Matti Pirinen, Nasa Sinnott-Armstrong, Mari E. K. Niemi, Mark J. Daly, Andrea Ganna, Hanna M. Ollila, VA Million Veteran Program, Daniel C. Posner, Alexis A. Rodriguez, Kelly Cho, Tianxi Cai, Sudha K. Iyengar, Shiuh-Wen Luoh, Ravi K. Madduri, MexGen-COVID Initiative, Ana Ochoa-Guzman, Alicia Huerta-Chagoya, Carlos A. Aguilar Salinas, Seung Hyuk T. Lee, Hortensia Moreno-Macias, Päivi Pajukanta, Michelle Duran-Gomez, Teresa Tusié-Luna, DBDS Genomic Consortium, Celia B. Sequeros, Sisse R. Ostrowski, Søren Brunak, David Westergaard, Bjarke Feenstra, Anne Sofie B. Mortensen, Christian Erikstrup, Ole B. V. Pedersen, Karina Banasik, Frank Geller, GEN-COVID Multicenter Study, Francesca Minnai, Simone Furini, Chiara Fallerini, Kristina Zguro, Margherita Baldassarri, Francesca Colombo, Alessandra Renieri, PHOSP-COVID Collaborative Group, Beatriz Guillen-Guio, Olivia C. Leavy, Louise V. Wain, GENCOV Study, Erika Frangione, Olga Vishnyakova, Xu Xinyi, Jennifer Taher, Lloyd T. Elliott, Jordan Lerner-Ellis, Estonian Biobank Research Team, Arne Kukkonen, Erik Abner, Shiuh-Wen Luoh, Christian Erikstrup, Ole B. V. Pedersen, Jordan Lerner-Ellis, Alicia Colombo, Joseph J. Grzymski, Makoto Ishii, Yukinori Okada, Noam D. Beckmann, Meena Kumari, Ralf Wagner, Iris M. Heid, Catherine John, Patrick J. Short, Per Magnus, Laura Ansone, Luca V. C. Valenti, Sulggi A. Lee, Louise V. Wain, Ricardo A. Verdugo, Karina Banasik, Frank Geller, Lude H. Franke, Alexander Rakitko, Emma L. Duncan, Alessandra Renieri, Konstantinos K. Tsilidis, Rafael de Cid, Ahmadreza Niavarani, Erik Abner, Teresa Tusié-Luna, Shefali S. Verma, George Davey Smith, Nicholas J. Timpson, Ravi K. Madduri, Kelly Cho, Mark J. Daly, Andrea Ganna, Eva C. Schulte, J. Brent Richards, Kerstin U. Ludwig, Michael Marks-Hultström, Hugo Zeberg, Hanna M. Ollila

**Affiliations:** 1 Institute for Molecular Medicine Finland (FIMM), Helsinki Institute of Life Science (HiLIFE), University of Helsinki, Helsinki, Finland; 2 Department of Human Genetics, McGill University, Montreal, Quebec Canada; 3 Centre for Clinical Epidemiology, Department of Medicine, Lady Davis Institute, Jewish General Hospital, McGill University, Montreal, Quebec Canada; 4 Kyoto-McGill International Collaborative Program in Genomic Medicine, Graduate School of Medicine, Kyoto University, Kyoto, Japan; 5 Department of Genome Informatics, Graduate School of Medicine, the University of Tokyo, Tokyo, Japan; 6 Research Fellow, Japan Society for the Promotion of Science, Tokyo, Japan; 7 Department of Psychiatry and Behavioral Sciences, University of California San Francisco, San Francisco, CA USA; 8 Program in Medical and Population Genetics, Broad Institute of Harvard and MIT, Cambridge, MA USA; 9 Stanley Center for Psychiatric Research, Broad Institute of Harvard and MIT, Cambridge, MA USA; 10 Analytic and Translational Genetics Unit, Massachusetts General Hospital, Boston, MA USA; 11 Genomes for Life-GCAT Lab, CORE Program, Germans Trias i Pujol Research Institute (IGTP), Badalona, Spain; 12 Grup de REcerca en Impacte de les Malalties Cròniques i les seves Trajectòries (GRIMTra), Barcelona, Spain; 13 Sano Genetics Limited, London, UK; 14 Unidad de Biología Molecular y Medicina Genómica, Instituto Nacional de Ciencias Médicas y Nutrición Salvador Zubirán, Mexico City, Mexico; 15 Genetics and Genomic Sciences, Icahn School of Medicine at Mount Sinai, New York City, NY USA; 16 Broad Institute, Cambridge, MA USA; 17 Analytical and Translational Genetics Unit, Massachusetts General Hospital, Harvard Medical School, Boston, MA USA; 18 Department of Mathematics and Statistics, University of Helsinki, Helsinki, Finland; 19 Department of Public Health, University of Helsinki, Helsinki, Finland; 20 Institute of Human Genetics, University of Bonn, School of Medicine and University Hospital Bonn, Bonn, Germany; 21 MRC Integrative Epidemiology Unit, University of Bristol, Bristol, UK; 22 Population Health Sciences, Bristol Medical School, University of Bristol, Bristol, UK; 23 Department of Hygiene and Epidemiology, University of Ioannina School of Medicine, Ioannina, Greece; 24 Department of Twin Research, King’s College London, London, UK; 25 Program in Metabolism and Program in Medical and Population Genetics, Broad Institute of MIT and Harvard, Cambridge, MA USA; 26 Center for Genomic Medicine and Diabetes Unit, Endocrine Division, Department of Medicine, Massachusetts General Hospital, Boston, MA USA; 27 Departamento de Medicina Genómica y Toxicología Ambiental, Instituto de Investigaciones Biomédicas, Universidad Nacional Autónoma de México, Mexico City, Mexico; 28 Unidad de Biología Molecular y Medicina Genómica, Instituto Nacional de Ciencias Médicas y Nutrición, Mexico City, Mexico; 29 Herbold Computational Biology Program, Public Health Sciences Division, Fred Hutchinson Cancer Center, Seattle, WA USA; 30 Department of Genome Sciences, University of Washington, Seattle, WA USA; 31 Finnish Institute of Molecular Medicine, University of Helsinki, Helsinki, Finland; 32 Helix, San Mateo, CA USA; 33 Mohn Center for Diabetes Precision Medicine, Department of Clinical Science, University of Bergen, Bergen, Norway; 34 Department of Genetics and Bioinformatics, Health Data and Digitalization, Norwegian Institute of Public Health, Oslo, Norway; 35 Computational Biology Unit, Department of Informatics, University of Bergen, Bergen, Norway; 36 Centre for Clinical Brain Sciences, Division of Psychiatry, University of Edinburgh, Edinburgh, UK; 37 Department of Genetics and Genomics, Mydnavar, Southfield, MI USA; 38 University of Helsinki, Helsinki, Finland; 39 Helsinki University Central Hospital, Helsinki, Finland; 40 VA Boston Healthcare System, Boston, MA USA; 41 Data Science and Learning, Argonne National Laboratory, Lemont, IL USA; 42 Department of Population Health Sciences, University of Leicester, Leicester, UK; 43 University Hospitals of Leicester NHS Trust, Leicester, UK; 44 Institute for Biomedical Technologies—National Research Council, Segrate, Italy; 45 Department of Medical Biotechnology and Translational Medicine (BioMeTra), Università degli Studi di Milano, Milan, Italy; 46 Institute for Social and Economic Research, University of Essex, Colchester, UK; 47 Department of Genetics, University Medical Center Groningen, University of Groningen, Groningen, the Netherlands; 48 Oncode Investigator, Utrecht, the Netherlands; 49 Novo Nordisk Foundation Center for Protein Research, Faculty of Health and Medical Sciences, University of Copenhagen, Copenhagen, Denmark; 50 Department of Genetic Epidemiology, University of Regensburg, Regensburg, Germany; 51 Charles Bronfman Institute for Personalized Medicine, Icahn School of Medicine at Mount Sinai, New York City, NY USA; 52 Department of Genetics and Genomic Sciences, Icahn School of Medicine at Mount Sinai, New York City, NY USA; 53 Latvian Biomedical Research and Study Centre, Riga, Latvia; 54 Università degli Studi di Milano, Milan, Italy; 55 Brigham and Women’s Hospital Division of Sleep and Circadian Disorders, Boston, MA USA; 56 Massachusetts General Hospital, Center for Genomic Medicine, Boston, MA USA; 57 Broad Institute, Molecular and Population Genetics Program, Cambridge, MA USA; 58 Center for Computational Biology, University of California Berkeley, Berkeley, CA USA; 59 The Institute for Lung Health, NIHR Leicester Biomedical Research Centre, University of Leicester, Leicester, UK; 60 Departamento de Oncología Básico Clínica, Facultad de Medicina, Universidad de Chile, Santiago, Chile; 61 Mount Sinai Hospital, Sinai Health, Toronto, Ontario Canada; 62 Department of Pathology and Laboratory Medicine, University of Pennsylvania, Philadelphia, PA USA; 63 Genotek Ltd, Moscow, Russia; 64 William Harvey Research Institute, Barts and the London School of Medicine and Dentistry, Queen Mary University of London, London, UK; 65 IRCCS G Gaslini, Genoa, Italy; 66 Department of Internal Medicine, Kulliyyah of Medicine, International Islamic University Malaysia, Pahang, Malaysia; 67 Department of Anatomy, All India Institute of Medical Sciences—Patna, Patna, India; 68 Faculty of Industrial Sciences and Technology, Universiti Malaysia Pahang Al Sultan Abdullah, Pahang, Malaysia; 69 Department of Statistical Genetics, Osaka University Graduate School of Medicine, Suita, Japan; 70 Department of Respiratory Medicine and Clinical Immunology, Osaka University Graduate School of Medicine, Suita, Japan; 71 Division of Pulmonary Medicine, Department of Medicine, Keio University School of Medicine, Tokyo, Japan; 72 Department of Respiratory Medicine, Nagoya University Graduate School of Medicine, Nagoya, Japan; 73 VA Portland Health Care System, Portland, Portland, OR USA; 74 Division of Hematology and Medical Oncology, Knight Cancer Institute, Oregon Health and Science University, Portland, OR USA; 75 Department of Clinical Immunology, Aarhus University Hospital, Aarhus, Denmark; 76 Department of Clinical Immunology, Zealand University Hospital—Køge, Køge, Denmark; 77 University of Toronto, Toronto, Ontario Canada; 78 Lunenfeld-Tanenbaum Research Institute, Sinai Health, Toronto, Ontario Canada; 79 Departamento de Anatomía Patológica, Facultad de Medicina, Universidad de Chile, Santiago, Chile; 80 Servicio de Anatomía Patológica, Hospital Clínico de la Universidad de Chile, Santiago, Chile; 81 Department of Internal Medicine, University of Nevada Reno, School of Medicine, Reno, NV USA; 82 Laboratory for Systems Genetics, RIKEN Center for Integrative Medical Sciences, Yokohama, Japan; 83 Laboratory of Statistical Immunology, Immunology Frontier Research Center (WPI-IFReC), Osaka University, Suita, Japan; 84 Division of Data Driven Medicine, Department of Medicine, Icahn School of Medicine at Mount Sinai, New York City, NY USA; 85 Institute of Medical Microbiology and Hygiene, Molecular Microbiology (Virology), University of Regensburg, Regensburg, Germany; 86 Institute of Clinical Microbiology and Hygiene, University Hospital Regensburg, Regensburg, Germany; 87 Leicester National Institute for Health and Care Research, Biomedical Research Centre, Glenfield Hospital, Leicester, UK; 88 Centre for Fertility and Health, Norwegian Institute of Public Health, Oslo, Norway; 89 Department of Pathophysiology and Transplantation, Università degli Studi di Milano, Milan, Italy; 90 Biological Resource Center, Fondazione IRCCS Ca’ Granda Ospedale Maggiore Policlinico, Milan, Italy; 91 Department of Medicine, Division of HIV, Infectious Diseases and Global Medicine, University of California, San Francisco, CA USA; 92 Instituto de Investigación Interdisciplinaria y Facultad de Medicina, Universidad de Talca, Talca, Chile; 93 Statens Serum Institute, Copenhagen, Denmark; 94 Department of Endocrinology, Guy’s and St Thomas’ NHS Foundation Trust, London, UK; 95 Department of Twin Research and Genetic Epidemiology, King’s College London, London, UK; 96 Medical Genetics, University of Siena, Siena, Italy; 97 Med Biotech Hub and Competence Center, Department of Medical Biotechnologies, University of Siena, Siena, Italy; 98 Genetica Medica, Azienda Ospedaliero-Universitaria Senese, Siena, Italy; 99 Department of Epidemiology and Biostatistics, School of Public Health, Imperial College London, London, UK; 100 Digestive Oncology Research Center, Digestive Disease Research Institute, Shariati Hospital, Tehran University of Medical Sciences, Tehran, Iran; 101 Estonian Genome Center, Institute of Genomics, University of Tartu, Tartu, Estonia; 102 Instituto de Investigaciones Biomédicas, UNAM, Mexico City, Mexico; 103 Instituto Nacional de Ciencias Médicas y Nutrición Salvador Zubirán, Mexico City, Mexico; 104 Data Science and Learning Division, Argonne National Laboratory, Lemont, IL USA; 105 Department of Medicine, Harvard Medical School and Mass General Brigham, Boston, MA USA; 106 Department of Psychiatry, University of Munich, Munich, Germany; 107 Institute of Human Genetics, University Hospital, Faculty of Medicine, University of Bonn, Bonn, Germany; 108 Institute of Virology, Technical University of Munich/Helmholtz Munich, Munich, Germany; 109 Institute of Psychiatric Phenomics and Genomics, University of Munich, Munich, Germany; 110 Department of Psychiatry, University Hospital, Faculty of Medicine, University of Bonn, Bonn, Germany; 111 5 Prime Sciences Inc, Montreal, Quebec, Canada; 112 Department of Epidemiology, Biostatistics and Occupational Health, McGill University, Montreal, Quebec Canada; 113 Lady Davis Institute of Medical Research, Jewish General Hospital, McGill University, Montreal, Quebec Canada; 114 Anaesthesiology and Intensive Care Medicine, Department of Surgical Sciences, Uppsala University, Uppsala, Sweden; 115 Department of Evolutionary Genetics, Max Planck Institute for Evolutionary Anthropology, Leipzig, Germany; 116 Department of Physiology and Pharmacology, Karolinska Institutet, Stockholm, Sweden; 117 Center for Genomic Medicine, Massachusetts General Hospital, Boston, MA USA; 118 Anesthesia, Critical Care, and Pain Medicine, Massachusetts General Hospital and Harvard Medical School, Boston, MA USA; 119 Broad Institute of MIT and Harvard, Cambridge, MA USA; 120 Victor Phillip Dahdaleh Institute of Genomic Medicine at McGill University and Department of Human Genetics, McGill University, Montreal, Quebec Canada; 121 Lady Davis Institute, Jewish General Hospital, McGill University, Montreal, Quebec Canada; 122 Research Centre of the Centre Hospitalier de l’Université de Montréal (CRCHUM), Montreal, Quebec Canada; 123 Centre hospitalier de l’Université de Montréal (CHUM), Montreal, Quebec Canada; 124 Institut universitaire de cardiologie et de pneumologie de Québec, Université Laval, Quebec, Quebec Canada; 125 The Meakins-Christie Laboratories at the Research Institute of the McGill University Heath, Centre Research Institute, and Department of Medicine, Faculty of Medicine, McGill University, Montreal, Quebec Canada; 126 Department of Psychiatry and Psychotherapy, University of Bonn, Bonn, Germany; 127 Center for Human Genetics, University Hospital of Marburg, Marburg, Germany; 128 Department of Biomedical Informatics, Harvard Medical School, Boston, MA USA; 129 Louis Stokes Cleveland VA Medical Center, Cleveland, Ohio, USA; 130 Department of Population and Quantitative Health Sciences, Case Western Reserve University, Cleveland, OH USA; 131 Instituto Nacional de Ciencias Medicas y Nutricion, Ciudad de México, Mexico; 132 Department of Human Genetics, David Geffen School of Medicine at UCLA, Los Angeles, CA USA; 133 Universidad Autonoma Metropolitana, Mexico City, Mexico; 134 Institute for Precision Health, David Geffen School of Medicine at UCLA, Los Angeles, CA USA; 135 Division of Infection Control, Norwegian Institute of Public Health, Oslo, Norway; 136 Department of Medicine, Department of Genetics, Division of Translational Medicine and Human Genetics, Institute for Translational Medicine and Therapeutics, University of Pennsylvania, Philadelphia, PA USA; 137 Department of Genetics, University of Pennsylvania, Philadelphia, PA USA; 138 Department of Medicine, Division of Translational Medicine and Human Genetics, Institute for Translational Medicine and Therapeutics, University of Pennsylvania, Philadelphia, PA USA; 139 Institute of Psychiatric Phenomics and Genomics (IPPG), University Hospital, LMU Munich, Munich, Germany; 140 Max-Planck Institute of Psychiatry, Munich, Germany; 141 Department of Psychiatry and Psychotherapy, University Medical Center Goettingen, Goettingen, Germany; 142 German Center for Neurodegenerative Diseases (DZNE), Goettingen, Germany; 143 Neurosciences and Signaling Group, Institute of Biomedicine (iBiMED), Department of Medical Sciences, University of Aveiro, Aveiro, Portugal; 144 Department of Internal Medicine II, University Hospital rechts der Isar, Technical University of Munich, School of Medicine, Munich, Germany; 145 Department of Psychiatry and Behavioral Sciences, Johns Hopkins University, Baltimore, MD USA; 146 Department of Psychiatry and Behavioral Sciences, SUNY Upstate Medical University, Syracuse, NY USA; 147 Department of Genetic Epidemiology in Psychiatry, Central Institute of Mental Health, Medical Faculty Mannheim, University of Heidelberg, Mannheim, Germany; 148 Institute of Clinical Chemistry and Pathobiochemistry, Klinikum rechts der Isar, School of Medicine, Technical University of Munich, Munich, Germany; 149 TranslaTUM, Center for Translational Cancer Research, Technical University of Munich, Munich, Germany; 150 German Center for Infection Research (DZIF), Munich Site, Braunschweig, Germany; 151 Institute of Computational Biology, Helmholtz Center Munich, Oberschleissheim, Germany; 152 Department of Psychosomatic Medicine and Psychotherapy, Klinikum rechts der Isar, School of Medicine, Technical University of Munich, Munich, Germany; 153 Department of Psychiatry and Psychotherapy, University Hospital, LMU Munich, Munich, Germany; 154 Institute of Psychiatric Phenomics and Genomics (IPPG), LMU University Hospital, LMU Munich, Munich, Germany; 155 Department of Psychiatry and Psychotherapy, University of Bern, Bern, Switzerland; 156 Department of Internal Medicine II, Klinikum Rechts der Isar, School of Medicine, Technical University of Munich, Munich, Germany; 157 Department of Twin Research and Epidemiology, King’s College London, London, UK; 158 Genómica Evolutiva y Médica de Magallanes (GEMMa), Centro Asistencial, Docente e Investigación (CADI-UMAG), Punta Arenas, Chile; 159 Escuela de Medicina, Universidad de Magallanes, Punta Arenas, Chile; 160 Interuniversity Center for Healthy Aging, Santiago, Chile; 161 Departamento de Ciencias de la Computación, Facultad de Ciencias Físicas y Matemáticas, Universidad de Chile, Santiago, Chile; 162 Molecular and Translational Immunology Laboratory, Department of Clinical Biochemistry and Immunology, Pharmacy Faculty, University of Concepción, Concepción, Chile; 163 Departamento de Tecnología Médica, Facultad de Ciencias de la Salud, Universidad de Antofagasta, Antofagasta, Chile; 164 Servicio de Anatomía, Hospital Clínico de la Universidad de Chile, Santiago, Chile; 165 ATACAMA OMICS, Laboratorio de Biología Molecular y Genómica, Facultad de Medicina, Universidad de Atacama, Copiapó, Chile; 166 Departamento de Tecnología Médica, Universidad de Tarapacá, Arica, Chile; 167 Instituto de Investigación Interdisciplinaria y Escuela de Medicina, Universidad de Talca, Talca, Chile; 168 AUSTRAL-omics, Vicerrectoría de Investigación Desarrollo y Creación Artística, Universidad Austral de Chile, Valdivia, Chile; 169 Unidades de Diagnóstico Fundación Arturo López Pérez, Providencia, Chile; 170 Facultad de Medicina, Universidad de Atacama, Copiapó, Chile; 171 Laboratorio Clínico del Área Técnica de Biología Molecular, Hospital del Salvador, Santiago, Chile; 172 Programa de Genética Humana del Instituto de Ciencias Biomédicas (ICBM), Facultad de Medicina, Universidad de Chile, Santiago, Chile; 173 Departamento de Oncología Básico Clínica, Facultad de Medicina and Departamento de Ciencias y Tecnología Farmacéutica, Universidad de Chile, Santiago, Chile; 174 Departamento de Ciencias y Tecnología Farmacéutica, Universidad de Chile, Santiago, Chile; 175 AUSTRAL-omics, Vicerrectoría de Investigación Desarrollo y Creación Artística, Valdivia, Chile; 176 Instituto de Ciencias Ambientales y Evolutivas, Facultad de Ciencias, Universidad Austral de Chile, Valdivia, Chile; 177 Department of Clinical Immunology, Copenhagen University Hospital—Rigshospitalet, Copenhagen, Denmark; 178 Department of Medical Endocrinology and Metabolism, Copenhagen University Hospital (Rigshospitalet), Copenhagen, Denmark; 179 ISGlobal, Hospital Clínic - Universitat de Barcelona, Barcelona, Spain; 180 CIBER de Enfermedades Infecciosas (CIBERINFEC), Barcelona, Spain; 181 Genomes for Life-GCAT lab, Barcelona, Spain; 182 Germans Trias i Pujol Research Institute (IGTP), Badalona, Spain; 183 Grup de REcerca en Impacte de les Malalties Cròniques i les seves Trajectòries (GRIMTra), (2021 SGR 01537), Badalona, Spain; 184 ISGlobal, Barcelona, Spain; 185 Universitat Pompeu Fabra (UPF), Barcelona, Spain; 186 CIBER Epidemiología y Salud Pública (CIBERESP), Madrid, Spain; 187 IMIM (Hospital del Mar Medical Research Institute), Barcelona, Spain; 188 Department of Electrical, Electronic and Information Engineering ‘Guglielmo Marconi’, University of Bologna, Cesena, Italy; 189 Department of Medical Biotechnologies, Med Biotech Hub and Competence Center, University of Siena, Siena, Italy; 190 Medical Genetics Unit, University of Siena, Policlinico Le Scotte, Siena, Italy; 191 Med Biotech Hub and Competence Centre, Department of Medical Biotechnologies, University of Siena, Siena, Italy; 192 Institute for Biomedical Technologies, National Reasearch Council, Segrate, Italy; 193 Eligens SIA, Riga, Latvia; 194 University of Nevada, School of Medicine, Reno, NV USA; 195 Renown Health, Reno, NV USA; 196 First Department of Internal Medicine and Infectious Diseases Unit, University Hospital of Ioannina, Ioannina, Greece; 197 Biomedical Research Foundation Academy of Athens, Athens, Greece; 198 Center for Evidence-Based Medicine, Department of Health Services, Policy and Practice, School of Public Health, Brown University, Providence, RI USA; 199 Department of Pulmonary and Critical Care, School of Medicine, Shariati Hospital, Tehran University of Medical Sciences, Tehran, Iran; 200 General Intensive Care Unit, Department of Anesthesiology, School of Medicine, Shariati Hospital, Tehran University of Medical Sciences, Tehran, Iran; 201 Intensive Care Unit, Department of Emergency, School of Medicine, Shariati Hospital, Tehran University of Medical Sciences, Tehran, Iran; 202 Department of Critical Care Medicine, Noorafshar Hospital, Tehran, Iran; 203 Department of Infectious Diseases, Keio University School of Medicine, Tokyo, Japan; 204 Department of Psychiatry, Icahn School of Medicine at Mount Sinai, New York City, NY USA; 205 Simon Fraser University, Burnaby, British Columbia Canada; 206 Brigham and Women’s Hospital and Harvard Medical School, Boston, MA USA; 207 Istituto di Ricerche Farmacologiche Mario Negri IRCCS, Milan, Italy; 208 Fondazione IRCCS Ca’ Granda Ospedale Maggiore Policlinico, Milan, Italy; 209 University of Oslo, Oslo, Norway

**Keywords:** Infectious diseases, Genome-wide association studies

## Abstract

Infections can lead to persistent symptoms and diseases such as shingles after varicella zoster or rheumatic fever after streptococcal infections. Similarly, severe acute respiratory syndrome coronavirus 2 (SARS‑CoV‑2) infection can result in long coronavirus disease (COVID), typically manifesting as fatigue, pulmonary symptoms and cognitive dysfunction. The biological mechanisms behind long COVID remain unclear. We performed a genome-wide association study for long COVID including up to 6,450 long COVID cases and 1,093,995 population controls from 24 studies across 16 countries. We discovered an association of *FOXP4* with long COVID, independent of its previously identified association with severe COVID-19. The signal was replicated in 9,500 long COVID cases and 798,835 population controls. Given the transcription factor FOXP4’s role in lung physiology and pathology, our findings highlight the importance of lung function in the pathophysiology of long COVID.

## Abstract (web summary)

A genome-wide study by the Long COVID Host Genetics Initiative identifies an association between the *FOXP4* locus and long COVID, implicating altered lung function in its pathophysiology.

## Main

The coronavirus disease 2019 (COVID-19) pandemic has led to the recognition of a new condition known as postacute sequelae of COVID-19 (PASC), post-COVID-19 condition or long COVID. The World Health Organization’s definition includes any symptoms that present typically within three months after COVID-19 and persist for at least two months^1^. Common symptoms include fatigue, pulmonary dysfunction, muscle and chest pain, dysautonomia and cognitive disturbances^2–6^. The incidence of long COVID varies widely, with estimates in severe acute respiratory syndrome coronavirus 2 (SARS‑CoV‑2)-infected individuals ranging from 10% to 70%^7^. Long COVID is more common in individuals who have been hospitalized or treated at the intensive care unit due to COVID-19 (refs. ^7,8^). However, long COVID can also occur in those with initially mild COVID-19 symptoms^9^. Moreover, several mechanisms may contribute to long COVID, including alterations of the serotonin system that may be related to cognitive changes^10^, mitochondrial mechanisms to fatigue^11^ and mechanisms involving complement and platelet activation to vascular disease observed in patients with long COVID^12^.

The COVID-19 Host Genetics Initiative (COVID-19 HGI) was launched to investigate host genetics in COVID-19 susceptibility, hospitalization and critical illness^13–16^. These findings implicate canonical pathways involved in viral entry, mucosal airway defense and type I interferon response^15–18^.

**Fig. 1:**
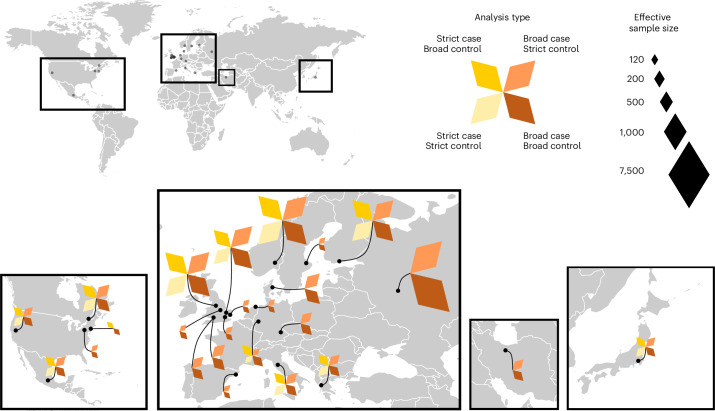
Geographic overview of studies contributing to the Long COVID HGI. The 24 studies contributing to the Long COVID HGI data freeze 4 served as the discovery cohorts for the GWAS meta-analyses. Each color represents a meta-analysis with specific case and control definitions. Strict case definition, long COVID after test-verified SARS-CoV-2 infection; broad case definition, long COVID after any SARS-CoV-2 infection; strict control definition, individuals that had SARS-CoV-2 but did not develop long COVID; broad control definition, population control, that is, all individuals in each study that did not meet the long COVID criteria. Effective sample sizes are shown as the size of each diamond shape, and locations of sample collection in (from left to right) North America, Europe, Middle East and Asia. For more detailed sample sizes, see Supplementary Table 11.

To elucidate biological mechanisms behind long COVID, we conducted a genome-wide association study (GWAS) and replication in 33 cohorts across 19 countries, totaling 15,950 individuals with long COVID and 1,892,830 controls (Fig. 1).

## Results

### Genetic variants in *FOXP4* locus associated with long COVID

We performed a meta-analysis of 24 independent GWAS of long COVID using two case definitions and two control definitions. A strict long COVID case definition required having an earlier test-verified SARS-CoV-2 infection (strict case definition), while a broader long COVID case definition also included self-reported or clinician-diagnosed SARS-CoV-2 infection (broad case definition). The broad definition included all contributing studies, whereas the strict definition included 11 studies (Supplementary Tables 11 and 12). Controls were either population controls, or participants that had recovered from SARS-CoV-2 infection without long COVID (strict control definition; Fig. 1 and Supplementary Tables 11 and 12). Data were obtained from 16 countries, representing populations from six genetic ancestries. The most common symptoms in the questionnaire-based studies were fatigue, shortness of breath and problems with memory and concentration. However, there was some heterogeneity in the frequency of symptoms (Supplementary Fig. 1).

**Fig. 2:**
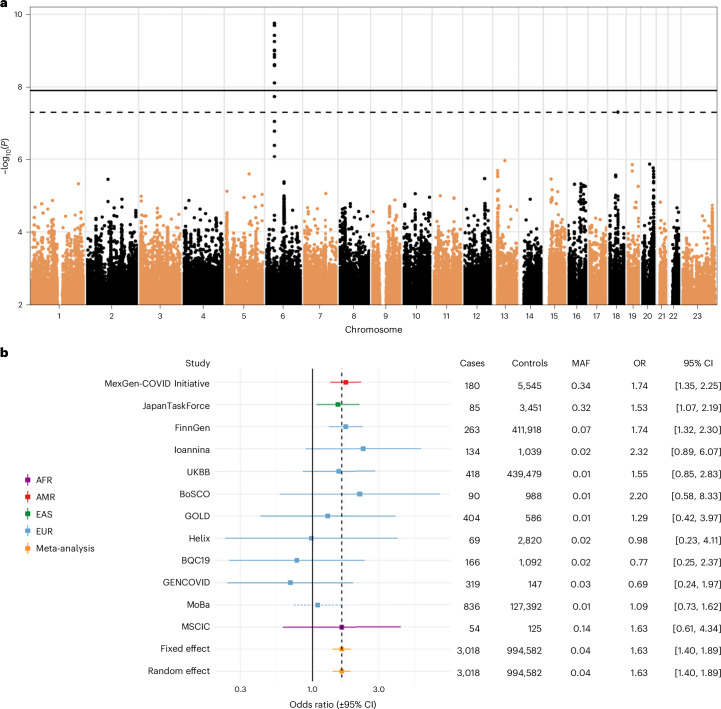
Meta-analysis of 11 GWAS studies of long COVID shows an association at the *FOXP4* locus. **a**, Manhattan plot of long COVID after test-verified SARS-CoV-2 infection (strict case definition, *n* = 3,018) compared to all other individuals in each dataset (population controls, broad control definition, *n* = 994,582). A genome-wide significant association with long COVID was found in the chromosome 6, upstream of the *FOXP4* gene (chr6: 41,515,652 G:C, GRCh38, rs9367106, as the lead variant; *P* = 1.76 × 10^−^^10^, Bonferroni *P* = 7.06 × 10^−^^10^, increased risk with the C allele, OR = 1.63, 95% CI = 1.40–1.89). Horizontal lines indicate genome-wide significance thresholds for IVW meta-analysis before (*P* < 5 × 10^−^^8^, dashed line) and after (1.25 × 10^−^^8^) Bonferroni correction over the four long COVID meta-analyses (INCMNSZ = MexGen-COVID Initiative). **b**, Chromosome 6 lead variant across the contributing studies and ancestries in GWAS meta-analyses of long COVID with strict case definition and broad control definition. Lead variant rs9367106 (solid line) and if missing, imputed by the variant with the highest LD with the lead variant for illustrative purpose, that is, rs12660421 (*r* = 0.98 in European in 1,000 G + HGDP samples^55^, dotted lines). For the imputed variants, *β* was weighted by multiplying by the LD correlation coefficient (*r* = 0.98). Centre, OR; error bar, 95% CI. Genetic ancestries marked by colors. MAF varies across ancestries, ranging from 1% to 34% (Supplementary Fig. 4). AFR, African; AMR, Admixed American; EAS, East Asian; EUR, European; UKBB, UK Biobank. (Results for the other three GWAS meta-analyses in Supplementary Figs. 2 and 3a–c).

The GWAS meta-analysis using the strict case definition (*n* = 3,018) and the broad control definition (*n* = 994,582) identified a genome-wide significant association within the *FOXP4* locus (chr6: 41,515,652 G > C, Genome Reference Consortium Human Build 38 (GRCh38), rs9367106, as the lead variant; *P* = 1.8 × 10^−10^; Fig. 2 and Supplementary Table 13). The C allele at rs9367106 was associated with an increased risk of long COVID (odds ratio (OR) = 1.63, 95% confidence interval (CI) = 1.40–1.89, risk allele frequency = 4.2%). The association replicated in an independent sample from eight additional contributing cohorts with 5,226 individuals with long COVID and 260,036 population controls (*P* = 0.025, OR = 1.13, 95% CI = 1.02–1.25; Supplementary Fig. 3d). Furthermore, the lead variants rs9367106 and rs12660421 replicated in the VA Million Veteran Program (MVP) in the strict case analyses with the broad control definition (*P* = 1 × 10^−^^4^, OR = 1.21, 95% CI = 1.10–1.34, long COVID cases, *n* = 4,274 and controls, *n* = 538,799; Supplementary Fig. 3e,f) and with the strict control definition (*P* = 0.0018, OR = 1.17, 95% CI = 1.06–1.29, long COVID cases, *n* = 4,274 and controls, *n* = 73,739; Supplementary Fig. 3g,h).

We observed an association, albeit not genome-wide significant, with rs9367106-C and long COVID also in all other three meta-analyses, including our largest meta-analysis with the broad case definition (*n* = 6,450) and the broad control definition (*n* = 1,093,995) from 24 studies (OR = 1.34, 95% CI = 1.20–1.49, *P* = 1.1 × 10^−^^7^; Supplementary Figs. 2 and 3). Analyses with the strict case definition (*n* = 2,964) and strict control definition (*n* = 37,935; OR = 1.30, 95% CI = 1.09–1.56, *P* = 3.8 × 10^−^^3^), and with the broad case definition (*n* = 6,396) and strict control definition (*n* = 46,208; OR = 1.16, 95% CI = 1.02–1.32, *P* = 0.023), further supported our findings (Supplementary Fig. 3).

**Fig. 3:**
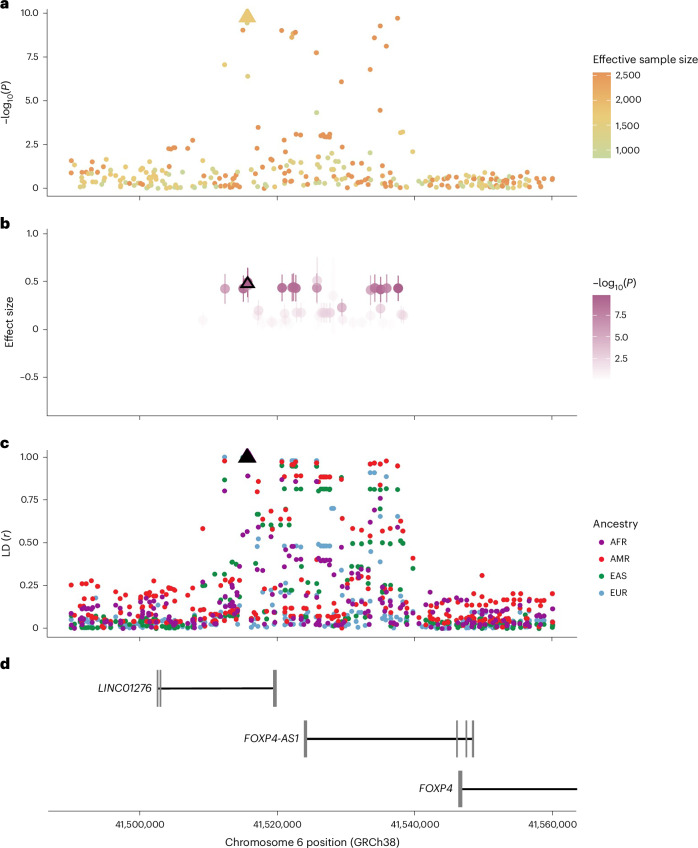
The chromosome 6 region (chr6: 41,490,001–41,560,000 (70 kb); *FOXP4* locus) in the long COVID GWAS meta-analysis. Long COVID meta-analysis with strict case (*n* = 3,018) and broad control (*n* = 994,582) definition (Fig. 2). *X* axis shows the position on chromosome 6 (GRCh38). The long COVID lead variant (rs9367106) is depicted with a triangle in each plot. **a**, Locus zoom plot with each variant colored by effective sample size and showing statistical significance (IVW GWAS meta-analysis −log_10_ *P* value) on *y* axis. **b**, Each variant colored by statistical significance and showing effect sizes (center, coefficients; error bar, 95% CI on *y* axis). **c**, Each variant colored by ancestry and showing LD correlation coefficient (*r*) with the long COVID lead variant on *y* axis. **d**, Ensembl genes in the region (*FOXP4* not fully shown; www.ensembl.org)^56^.

To examine the consistency of the *FOXP4* signal across the contributing studies, we investigated the effect in each study (Fig. 2b). Genetic variants in the meta-analysis had varying statistical power due to missingness, due to genotyping and imputation quality, and due to differences in allele frequency differences between populations. Therefore, the genetic variant that was present in majority of the studies was the most statistically significant variant, not necessarily because it is the causal variant but because it had the best statistical power. We, therefore, examined the effect size of variants within 30 kb around the lead variant (rs9367106, *r*^2^ > 0.01 in individuals of Europeans in the Human Genome Diversity Project^19^ and 1000 Genomes Project^20,21^) and effective sample size of at least one-third the sample size of the lead variant. Through this analysis, we identified a haplotype spanning the genomic region chr6:41,512,355–41,537,458 located upstream of *FOXP4* gene (Fig. 3d), for which variants had *P* values less than 5 × 10^−^^7^ (Fig. 3a) and effect sizes similar to the lead variant across ancestries (Fig. 3b,c). This analysis identified 15 variants (Supplementary Table 14). Relying on linkage disequilibrium (LD) in the 1000 Genomes Project across African, East Asian European, admixed American and South Asian populations, we found 18 variants cosegregating with the lead variant with tightest LD at the end of the haplotype (*r*^2^ > 0.5; Supplementary Table 15). Nine variants overlapped between these two analyses.

### Frequency of long COVID variants varies across ancestries

The allele frequency of rs9367106-C at the *FOXP4* locus varied across the study populations ranging from 1.6% in non-Finnish Europeans to 7.1% in Finnish, 19% in admixed Americans and 36% in East Asians (Supplementary Fig. 4; https://gnomad.broadinstitute.org/variant/6-41515652-G-C?dataset=gnomad_r3). Most of the contributing studies comprised individuals of European ancestry (Supplementary Fig. 5). Despite smaller sample sizes, we observed significant associations in admixed American, East Asian and Finnish ancestries (Fig. 2b), owing to the higher allele frequency, and thus larger statistical power to detect an association with the rs9367106 variant in these cohorts.

### Risk variants, *FOXP4* expression and COVID-19 severity

**Fig. 4:**
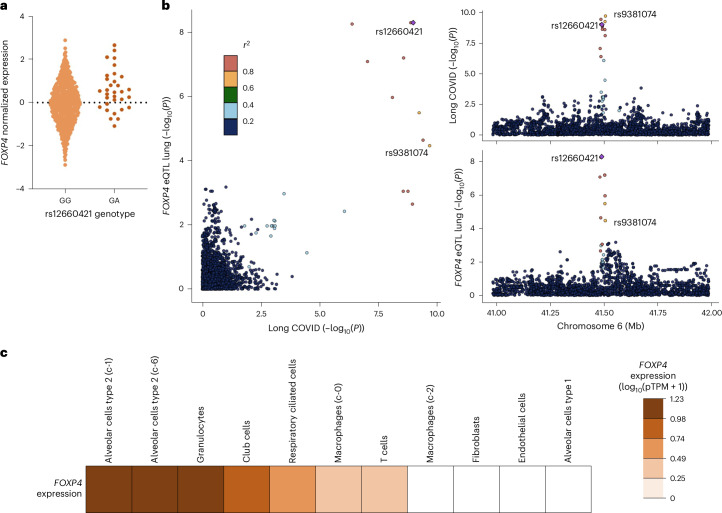
*FOXP4* expression in the lung. **a**, The lead variant rs9367106 was not found in the GTEx dataset, but a proxy variant (rs12660421, chr6: 41,520,640) in high LD (*r*^2^ = 0.97, rs12660421-A allele is correlated with the long COVID risk allele rs9367106-C) showed a significant eQTL after multiple testing correction, increasing *FOXP4* expression in the lung (*P* = 5.3 × 10^−9^, NES; expression with GA genotype compared to expression with GG, normalized to 0) = 0.56; GTEx V8 lung samples with GG genotype, *n* = 483, GA genotype, *n* = 32; https://gtexportal.org/home/snp/rs12660421). For other tissues, see multitissue eQTL plot in Supplementary Fig. 6. **b**, Colocalization analysis using eQTL data from GTEx v8 tissue type and long COVID GWAS meta-analysis association data (Supplementary Note). Plots illustrate −log_10_ *P* value for long COVID (*x* axis) and for *FOXP4* expression in the lung (*y* axis), regional association of the *FOXP4* locus variants with long COVID (top right) and regional association of the *FOXP4* variants with RNA expression measured in the lung in GTEx (bottom right). Variants are colored by 1000 Genomes European-ancestry LD *r*^2^ with the lead variant (rs12660421) for *FOXP4* expression in lung tissue (the most significant long COVID variant overlapping the GTEx v8 dataset (rs9381074) also annotated). **c**, Human Protein Atlas RNA single-cell type tissue cluster data (transcript expression levels summarized per gene and cluster) of lung (GSE130148) showing *FOXP4* expression in unaffected individuals. The values were visualized using log_10_ (pTPM + 1) values. Each annotation is taken from the clustering results performed in the Human Protein Atlas. pTPM, protein transcripts per million.

We next investigated whether the long COVID variants were associated with differential expression of any of the surrounding genes within a 100-kb window (*FOXP4*, *FOXP4-AS1*, *LINC01276* and *MIR4641*). We found that rs12660421-A is associated with an increase in *FOXP4* expression in the lung (*P* = 5.3 × 10^−^^9^, normalized effect size (NES) = 0.56) and in the hypothalamus (*P* = 2.6 × 10^−^^6^, NES = 1.4; Fig. 4a and Supplementary Fig. 6; GTEx, https://gtexportal.org/home/snp/rs12660421). Furthermore, there were no additional expression quantitative trait loci (eQTL) or colocalization with the expression of *FOXP4-AS1* (Supplementary Table 16). *FOXP4* (HUGO Gene Nomenclature Committee ID: 20842) is a transcription factor gene that has a broad tissue expression pattern and is expressed in nearly all tissues, with the highest expression in the cervix, the thyroid, the vasculature, the stomach and the testis^22^. The expression also spans a broad set of cell types, including endothelial lung cells, immune cells and myocytes^23^. A colocalization analysis suggested that the association signal of long COVID is the same signal that associates with the differential expression of *FOXP4* in the lung (posterior probability = 0.91; Supplementary Fig. 7a,b and Supplementary Table 17).

Furthermore, variants in the *FOXP4* region have also been identified as risk factors for COVID-19 hospitalization, colocalizing with *FOXP4* expression eQTL in the COVID-19 HGI meta-analyses and follow-up studies^16,24^ (Supplementary Fig. 8 and Supplementary Table 18). Our colocalization analysis demonstrated the *FOXP4* association identified here as the same association identified for COVID-19 severity (posterior probability > 0.97; Supplementary Fig. 7e,f and Supplementary Table 17).

### *FOXP4* expression in blood is associated with long COVID

To understand whether higher *FOXP4* expression was seen in long COVID, we collected blood samples from participants with or without active SARS-CoV-2 infection. We discovered that the higher *FOXP4* levels in nonacute COVID-19 samples were associated with increased risk of long COVID (OR = 2.31 per 1 s.d. increase in *FOXP4* expression, 95% CI = 1.27–4.22, *P* = 0.0063; Supplementary Fig. 9), while *FOXP4* levels in acute COVID-19 samples were not associated with long COVID (*P* = 0.62). This is orthogonal evidence to the genetic signal that higher *FOXP4* levels may lead to long COVID.

### *FOXP4* expression in alveolar and immune cells in the lung

As lung tissue consists of several cell types, we wanted to elucidate the relevant cells that express *FOXP4* and may contribute to long COVID. We analyzed single-cell sequencing data from the Tabula Sapiens, a previously published atlas of single-cell sequencing data in healthy individuals free of COVID-19^25^. *FOXP4* expression was the highest in type 2 alveolar cells in individuals without SARS-CoV-2 infection (Fig. 4c) and during active infection (Supplementary Fig. 10), suggesting that SARS-CoV-2 infection was not required for *FOXP4* expression. Furthermore, type 2 alveolar cells are capable of mounting robust innate immune responses, thus participating in the immune regulation in the lung. Additionally, type 2 alveolar cells secrete surfactant, keep the alveoli free from fluid, and serve as progenitor cells repopulating damaged epithelium after injury^26^. In addition, we observed nearly equally high expression of *FOXP4* in granulocytes that similarly participate in the regulation of innate immune responses. Overall, the findings suggest a possible role of both immune and alveolar cells in the lung and higher expression of *FOXP4* in long COVID.

### *FOXP4* variants located at active chromatin in the lung

To understand the possible causal variation at the *FOXP4* locus, we performed statistical fine mapping using SLALOM^27^ (Supplementary Note). There were nine variants within the 95% credible set with the maximum posterior probability of 0.28 for rs9381074 (Supplementary Fig. 11). Given the strong LD pattern among the nine variants within the credible set, fine mapping alone might not be able to pinpoint a single causal variant in this locus. Therefore, to understand possible functional regulatory effects behind the variant association, we used the data from the Regulome database^28,29^, ENCODE^30^ and VannoPortal^31^. While the majority of the long COVID variants were at active enhancer or transcription factor binding sites, four variants had direct evidence of transcription factor binding based on chromatin immunoprecipitation sequencing experiments (Supplementary Tables 19 and 20). One of these variants (rs9381074) was directly located on a region that had DNA methylation marks across multiple tissues, including immune and lung cells (H3K27me3 and H3K4me1, H3K4me3, H3K27ac, H3K4me2 and H3K4me3), and had evidence of transcriptional activity from 49 different transcription factors, of which we saw the most consistent direct binding of FOXA1 across 55 experiments. Furthermore, we downloaded DNase sequencing data from the ENCODE project and observed that rs9381074 was directly positioned on a DNase hypersensitivity site in the lung (Supplementary Note). Finally, this variant is the same variant implicated by statistical fine mapping, suggesting the rs9381074 variant as the causal variant for association at the *FOXP4* locus.

### *FOXP4* variant associated with lung cancer

To understand the role of *FOXP4* and its associations across diseases, we performed phenome-wide association analysis. We first focused on Biobank Japan^32^, as the long COVID risk allele frequency is highest in East Asia. Phenome-wide association study (PheWAS) between rs9367106 and all phenotypes in Biobank Japan (*n* = 262) revealed that long COVID risk allele was associated with lung cancer (*P* = 1.2 × 10^−6^, Bonferroni *P* = 3.1 × 10^−^^4^, OR = 1.13, 95% CI = 1.07–1.18; Supplementary Fig. 8 and Supplementary Table 18). Furthermore, the long COVID risk allele is in LD with the known risk variants for non-small cell lung carcinoma in Chinese and European populations^33^ (rs1853837, *r*^2^ = 0.88 in East Asians^34^) and for lung cancer in never-smoking Asian women^35^ (rs7741164, *r*^2^ = 0.98 in East Asians^34^). Colocalization analysis supported that the associations in this locus (within 500 kb of rs9367106) for long COVID and lung cancer shared the same genetic signal (colocalization posterior probability = 0.98; Supplementary Fig. 7c,d). COVID-19 phenotypes and lung cancer traits were the only associations found with linked variants in the GWAS Catalog (Supplementary Table 21).

We then broadened the analysis to other cohorts. Using data from FinnGen and Open Targets, we observed a robust gene level PheWAS association with prostate cancer, immune traits including reticulocytes and chronotype (Supplementary Tables 22–24). Moreover, colocalization analysis provided by Open Targets showed that *FOXP4* expression and *FOXP4* splice QTLs colocalized with blood count traits specifically in the blood and the thyroid, but the blood count traits did not colocalize with the expression in the lung (Supplementary Table 25). These findings suggest that separate regulatory variation may contribute to tissue-specific expression and the control of otherwise ubiquitously expressed *FOXP4* and contribute to trait associations in a tissue-specific manner.

### Long COVID and other phenotypes

**Fig. 5:**
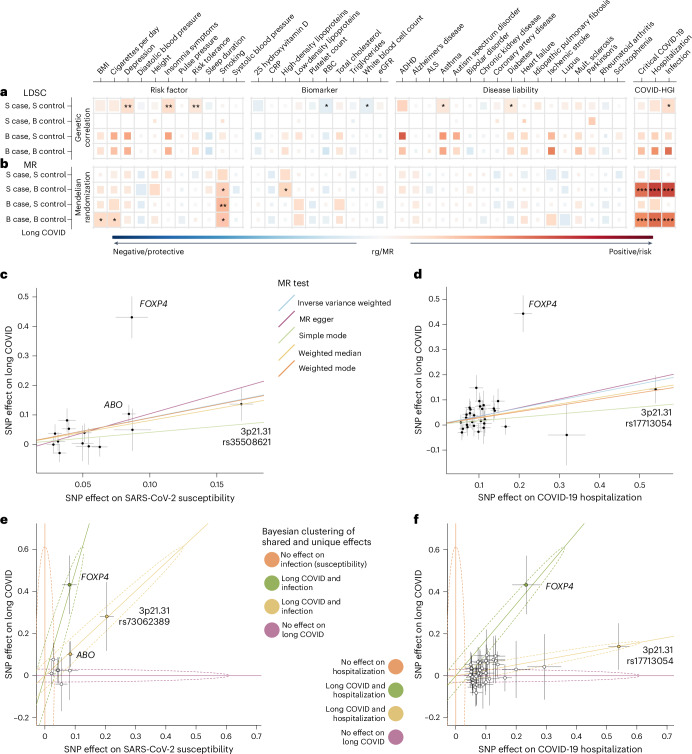
Genetic correlations and MR causal estimates between long COVID and potential risk factors, biomarkers and diseases. **a**,**b**, LD score regression (**a**, LDSC, top; Supplementary Table 27) and IVW MR (**b**, fixed-effects model,bottom; Supplementary Table 29 and Supplementary Data) were used for calculating two-sided *P* values. The size of each colored square corresponds to statistical significance (***P < 0.0001, full-sized square; **P < 0.01, full-sized square; *P < 0.05, full-sized square; *P* < 0.1, large square; *P* < 0.5, medium square and *P* > 0.5, small square; not corrected for multiple comparisons). A full list of traits is provided in Supplementary Table 26. For sample sizes in each long COVID GWAS meta-analysis using strict (S) or broad (B) case and control definitions, see Supplementary Table 11. **c**, MR scatter plot with effect sizes (*β* ± s.e.) of each variant on COVID-19 susceptibility (reported SARS-CoV-2 infection) as exposure and long COVID (strict case, broad control definition) as outcome (*P* (IVW, fixed effects) = 1.8 × 10^−7^, pleiotropy *P* = 0.47; Supplementary Table 30). **d**, Similarly, MR with COVID-19 hospitalization as exposure and long COVID as outcome (*P* (IVW fixed effects) = 4.8 × 10^−8^, pleiotropy *P* = 0.83; Supplementary Table 30). **e**, Analysis of shared and unique effects between SARS-CoV-2 infection susceptibility and long COVID using a Bayesian mixture model showed *ABO* and 3p21.31 rs73062389 as having shared effects (posterior probability > 0.99). *FOXP4* variant association was discovered in the long COVID meta-analyses but showed also an effect on the susceptibility of the initial infection, though smaller than on long COVID (Supplementary Table 34). (Effects shown as *β*, error bars represent 95% confidence intervals.) **f**, Similarly, analysis of shared and unique effects between COVID-19 severity and long COVID using a Bayesian mixture model showed *FOXP4* variant with a joint effect (posterior probability > 0.9), differing from the other severity variants due to its larger effect on long COVID (Supplementary Table 35). BMI, body mass index; CRP, C-reactive protein; eGFR, estimated glomerular filtration rate; ADHD, attention-deficit hyperactivity disorder.

We investigated the relationship between long COVID and cardiometabolic, behavioral and psychiatric traits^36^ (Fig. 5 and Supplementary Table 26). We found positive genetic correlations between long COVID and insomnia symptoms, depression, risk tolerance, asthma, diabetes and SARS-CoV-2 infection, while we saw negative correlations with red and white blood cell counts (Fig. 5a). However, identified correlations were only nominally significant without multiple testing correction (*P* < 0.05; Supplementary Table 27). The observed scale heritability estimates of long COVID ranged from 0.97% to 12.36% (s.e. = 0.0362), with the highest heritability in the strict case and strict control definitions (Supplementary Table 28).

We used Mendelian randomization (MR) to estimate potential risk factors by analyzing the same traits mentioned above (Supplementary Table 26). Genetically predicted earlier smoking initiation (*P* = 0.022), more cigarettes consumed per day (*P* = 0.046), higher levels of high-density lipoproteins (*P* = 0.029) and higher body mass index (*P* = 0.046) were nominally significant causal risk factors of long COVID (Fig. 5b and Supplementary Table 29). However, none of these associations survived correction for multiple comparisons.

### *FOXP4* signal not explained simply by COVID-19 severity

Earlier research has suggested that COVID-19 severity is a risk factor for long COVID^8,37–39^ and *FOXP4* variants have earlier been implicated in COVID-19 severity^6^. Our initial GWAS and robust replication across different cohorts show *FOXP4* variants also associated with long COVID. However, the results pose an interesting question of whether the mechanism of *FOXP4* association with long COVID is the same mechanism that contributes to COVID-19 severity. We thus investigated the relationship between COVID-19 hospitalization and long COVID by performing a two-sample MR (Supplementary Table 30). In terms of causality, we caution that COVID-19 hospitalization as causal exposure is difficult to interpret because both long COVID and COVID-19 hospitalization are two outcomes of the same underlying infection. Nevertheless, the relationship between the effect size for long COVID versus the effect size for COVID-19 severity can shed some light on the role of COVID-19 severity in long COVID. To perform two-sample MR without overlapping samples, we have excluded the studies that contributed to the current long COVID freeze 4 and computed a meta-analysis of SARS-CoV-2 infection susceptibility and COVID-19 hospitalization of the remaining cohorts in the COVID-19 HGI. We observed a causal relationship of susceptibility and hospitalization on long COVID (strict case and broad control definition; inverse variance-weighted (IVW) MR, *P* = 1.8 × 10^−7^ for infection and *P* = 4.8 × 10^−8^ for hospitalization) with no evidence of pleiotropy (MR–Egger intercept *P* = 0.47 and 0.83, respectively; Fig. 5c,d and Supplementary Table 30). Furthermore, sensitivity analysis by leaving one variant out (Supplementary Table 31), or by including long COVID cohorts with European-ancestry only (Supplementary Table 32), both supported a robust causal association between COVID hospitalization and long COVID. Nevertheless, the Wald ratio of long COVID to COVID-19 hospitalization for the *FOXP4* variant is 1.97 (95% CI = 1.36–2.57), which is significantly greater than the slope of the MR-estimated relationship between COVID-19 hospitalization and long COVID (0.35, 95% CI = 0.12–0.57). Furthermore, adjusting or stratifying the long COVID GWAS for hospitalization did not explain the association between *FOXP4* and long COVID (Supplementary Table 33a).

Thus, the *FOXP4* signal demonstrates a stronger association with long COVID than expected, meaning that it cannot simply be explained by its association with either susceptibility or severity of the acute disease alone (Fig. 5c,d). A recent systematic review of epidemiological data found a positive association between COVID-19 hospitalization and long COVID with a relationship on a log-odds scale of 0.91 (95% CI = 0.68–1.14)^40^. Even assuming this stronger relationship between COVID-19 hospitalization and long COVID, the observed effect of the *FOXP4* variant on long COVID still exceeds what would be expected based on the association with severity alone.

When SARS-CoV-2 infection is required for COVID-19 disease, and for severe COVID-19, an important question is whether all genetic variants that increase COVID-19 susceptibility or severity are equally large risk factors for long COVID. Bayesian methods provide an opportunity to estimate whether some variants that affect COVID-19 susceptibility or severity systematically contribute to the risk of long COVID more than the other variants. To answer this question, we estimated the posterior probabilities for all susceptibility and severity variants for long COVID using four models—susceptibility/severity only, long COVID only and two models for joint effects that differed in their slopes. We observed that for COVID-19 susceptibility, the 3p21.31 locus and the *ABO* locus contributed to both susceptibility and long COVID with a high posterior probability (Fig. 5e and Supplementary Table 34). Moreover, while many severity variants are also likely to contribute to long COVID, their slope between long COVID and severity effects was smaller than that of *FOXP4* (Fig. 5f and Supplementary Table 35).

Finally, previous studies have shown a potential effect of vaccination, strain and severity on long COVID^5,7,41–44^. To clarify these factors with long COVID, we used data from additional cohorts, including FinnGen. We observed that, while adjusting for severity or vaccination status did not remove the signal, there was a possible stronger risk of *FOXP4* risk alleles before vaccination and with wild-type and Alpha strains (Supplementary Table 33b,c). A significant association of the *FOXP4* locus with long COVID in individuals before vaccination was observed. Although the effect remained positive postvaccination (OR = 1.3), the lack of significant association in these cases may be influenced by the relatively small sample size of individuals diagnosed with long COVID after vaccination (*n* = 40; Supplementary Table 33b). Earlier epidemiological studies have shown that immunization against COVID-19 is associated with a reduced risk of long COVID^43–45^. Our data are in line with these earlier observations. Furthermore, we sought replication for the strain association in the Estonian Biobank, where higher risk was also observed with earlier strains, particularly the Alpha strain (*P* = 0.0138).

The possible time-dependent association with strain prompted us to explore the temporal relationship between *FOXP4* and long COVID from the start of the year 2020 till the spring of 2023. Using data from 3,684 individuals with long COVID from FinnGen, we observed a significant temporal association with the Cox proportional hazards model (HR = 1.3, 95% CI = 1.1–1.7, *P* = 0.005, *n*_population controls_ = 496,664; Supplementary Fig. 12). Moreover, particularly homozygosity for the *FOXP4* risk allele increased the risk for long COVID (recessive *P* = 2.3 × 10^−^^4^, OR = 5.64, 95% CI = 2.25–14.17). Moreover, we observed a consistently higher risk allele homozygosity among long COVID cases in the Estonian Biobank and MexGene-COVID (Supplementary Note). Overall, these results indicate a temporal relationship with *FOXP4* risk variants on long COVID and higher risk with homozygosity and earlier viral strains. In all these analyses, *FOXP4* stood out as an independent risk factor for long COVID.

### *FOXP4* associates with multiple symptoms of long COVID

We aimed to investigate the symptomatic associations between *FOXP4* and long COVID. We focused on well-established components of long COVID as documented in earlier literature^7^. Using symptom data from the two largest cohorts, FinnGen and MVP, we re-examined the association of *FOXP4* with long COVID, requiring lifetime symptoms from any of the previously identified subtypes. Our analysis revealed consistent associations across both MVP and FinnGen cohorts, with fatigue and asthma diagnoses, and β-adrenergic and proton pump inhibitor medication showing significant associations in the meta-analysis of the two cohorts (Supplementary Fig. 13 and Supplementary Table 36). The replication of these associations in datasets from two different countries, with distinct healthcare settings and patient populations, strengthens the robustness of the link between *FOXP4* and the plethora of manifestations of long COVID.

## Discussion

In this study, we aimed to understand the host genetic factors that contribute to long COVID, using data from 24 studies across 16 countries and replicating in independent cohorts. Our analysis identified genetic variants within the *FOXP4* locus as a risk factor for long COVID. The *FOXP4* gene is expressed in the lung and the genetic variants associated with long COVID are also associated with differential expression of *FOXP4* and with lung cancer and COVID-19 severity. Additionally, using MR, we characterized COVID-19 severity as a causal risk factor for long COVID. Overall, our findings provide genomic evidence consistent with previous epidemiological and clinical reports of long COVID, indicating that long COVID, similarly to other postviral conditions, is a heterogeneous disease entity where likely both individual genetic variants and the environmental risk factors contribute to disease risk.

Our analysis revealed a connection between long COVID and pulmonary endpoints through both individual variants at *FOXP4*, a transcription factor-coding gene previously linked to lung cancer and COVID-19 severity^24^, and MR analysis identifying smoking and COVID-19 severity as risk factors. Furthermore, expression analysis of the lung, and cell type-specific single-cell sequencing analysis, showed *FOXP4* expression in both alveolar cell types and immune cells of the lung.

*FOXP4* belongs to the subfamily P of the forkhead box transcription factor family genes and is expressed in various tissues, including the lungs and the gut^45,46^. Moreover, it is highly expressed in mucus-secreting cells of the stomach and intestines^47^, as well as in naïve B, natural killer and memory T_reg_ cells^48^, and required for normal T cell memory function following infection^49^. *FOXP1*/*FOXP2*/*FOXP4* are also required for promoting lung endoderm development by repressing expression of nonpulmonary transcription factors^50^, and the loss of *FOXP1*/*FOXP4* adversely affects airway epithelial regeneration^51^. Furthermore, *FOXP4* has been implicated in airway fibrosis^52^ and the promotion of lung cancer growth and invasion^53^. We find that the variants associated with long COVID are also associated with lung cancer in Biobank Japan^32^. These observations together with the present study may suggest that the connection between *FOXP4* and long COVID may be rooted in both lung function and immunology. Furthermore, *FOXP4* expression in both alveolar and immune cells in the lung, and the association with severe COVID-19 and pulmonary diseases such as cancer, suggests that *FOXP4* may participate in local immune responses in the lung.

Our functional analysis further implicated *FOXP4* as a risk factor for long COVID, irrespective of the genotype status of the here-identified risk variant. *FOXP4* expression levels were higher in individuals with long COVID than controls. Furthermore, we observed a consistent effect of *FOXP4* risk variants across ancestries. Moreover, having multiple ancestries enabled us to fine-map a likely causal variant at rs9381074, which was further supported by functional methylation and expression data.

We also discovered a causal relationship between SARS-CoV-2 infection and long COVID, as expected, and an additional causal risk between severe, hospital treatment-requiring COVID-19 and long COVID. This finding is in agreement with earlier epidemiological observations^8,37–39^. The relationship between COVID-19 severity and long COVID raises an interesting question—when SARS-CoV-2 infection is required for both COVID-19 and severe COVID-19, are all genetic variants that increase COVID-19 susceptibility or severity equally large risk factors for long COVID? In the present study, we aimed to answer this question by examining variant effect sizes between SARS-CoV-2 infection susceptibility, COVID-19 severity and long COVID using stratified and adjusted analyses, and by Bayesian modeling. Among the known SARS-CoV-2 susceptibility loci, *ABO* and 3p21.31 had a high probability of also contributing to long COVID. Moreover, the *FOXP4* variants had higher effect sizes for long COVID than expected based on the other severity variants, suggesting an independent role of *FOXP4* for long COVID that was not observed among the other COVID-19 severity variants. Such observation offers clues on biological mechanisms, such as *FOXP4* affecting pulmonary function and immunity, which then contribute to the development of long COVID. Overall, our study elucidates genetic risk factors for long COVID, the relationship between long COVID and severe COVID-19, and finally possible mechanisms of how *FOXP4* contributes to the risk of long COVID.

Moreover, while several lines of evidence from the original GWAS association, replication, stratified analyses to Bayesian analysis and the significance of individual variants suggest that *FOXP4* contributes to long COVID in a stronger way than expected, the mechanism that *FOXP4* associates with long COVID may be the same mechanism that contributes to COVID-19 severity. Future studies and iterations of this work will likely grow the number of observed genetic variants and further clarify the biological mechanisms underlying long COVID. We also caution that the genetic predisposition to long COVID might be dependent on SARS-CoV-2 variation and vaccination status, and that a large portion of our data was collected before the omicron wave and widespread vaccination (Supplementary Table 12), which might have an impact on the genetic associations.

The contribution of genetic factors to COVID-19 phenotypes is intriguing. As heritability in general is defined as the proportion of phenotypic variation attributable to genetic differences within a specific environment, in a hypothetical world where every environmental factor would be similar, heritability would theoretically approach 100%. However, as the heritability in infections can be shaped by exposure, viral strain, prophylactics, earlier immunity, for example, through vaccination efforts, or differences in diagnostic criteria, reporting or local recommendations, estimating heritability requires relatively large samples for precise estimates. Similarly, heritability in earlier studies of COVID-19 phenotypes was initially less than 1% for COVID-19 susceptibility, severity and critical illness even with over 46,000 COVID-19 cases and 2 million controls^6^. However, all COVID-19 traits showed robust genetic correlations with the known COVID-19 epidemiological risk factors. In our study, we similarly see low heritability with long COVID, which is a limitation in the current study. Nonetheless, the estimate provides a tool to understand between-trait correlations and will likely become more precise with larger sample sizes.

We recognize that the symptomatology of long COVID is variable and includes, in addition to lung symptoms, also other symptom domains such as fatigue and cognitive dysfunction^7,37,54^. In addition, the long-term effects of COVID-19 are still being studied, and more research is needed to understand the full extent of the long-term damage caused by SARS-CoV-2 and long COVID disease. We also recognize that the long COVID diagnosis is still evolving. Nevertheless, our study provides direct genetic evidence that lung pathophysiology can have an integral part in the development of long COVID.

## Methods

### Contributing studies

Participants of each of the contributing 33 studies provided written informed consent to participate in each respective study, with recruitment and ethics following study-specific protocols approved by their respective institutional review boards (details are provided in Supplementary Table 12).

For the initial discovery analysis, we used data from the following 24 studies: Avon Longitudinal Study of Parents and Children (ALSPAC), Bonn Study of COVID Genetics (BoSCO), Banque québécoise de la COVID-19 (BQC19), Danish Blood Donor Study (DBDS), Extended Cohort for E-health, Environment and DNA (EXCEED), FinnGen, GCAT | Genomes for life, Genetic Bases of COVID-19 Clinical Variability (GEN-COVID), Genotek, Genetics of Long COVID (GOLD), Helix Exome+ and Healthy Nevada Project COVID-19 Phenotypes (Helix), MexGen-COVID Initiative, COVID-19 Ioannina Biobank (Ioannina), Genome-wide assessment of the gene variants associated with severe COVID-19 phenotype in Iran (IrCovid), Japan COVID-19 Task Force (JapanTaskForce), Lifelines, Norwegian Mother, Father and Child Cohort Study (MoBa), Mount Sinai COVID Biobank (MSCIC), Penn Medicine BioBank (PMBB), Follow-UP study of patients with critical COVID-19/COVID-19 Cohort Study of the University Hospital of the Technical University Munich (SweCovid/COMRI), Tirschenreuth Study (TiKoCo), TwinsUK, UK Biobank and Understanding Society—UK Household Longitudinal Study. The total sample size of this Long COVID HGI data freeze 4 was 6,450 long COVID cases, 46,208 COVID-19-positive controls and 1,093,955 population controls (Supplementary Table 12). For the replication of the *FOXP4* lead variants, we obtained data from the following nine additional studies: COVID-19 cohort at LGDB (LatviaGDB), COVID-19 Genomics Network (C19-GenoNet), COVID-19 Host Immune Response Pathogenesis Study (CHIRP), Estonian Biobank (EstBB), Fondazione Genomics SARS-CoV-2 Study (FoGS), GENCOV Study (GENCOV), Mass General Brigham Biobank (MGB), The Post-hospitalization COVID-19 study (PHOSP-COVID) and VA MVP. The replication datasets together comprised 9,500 individuals with long COVID and 798,835 population controls (Supplementary Fig. 3d,e and Supplementary Table 12).

The effective sample sizes for each study shown in Fig. 1 were calculated for display using the given formula: (4 × *n*_case_ × *n*_control_)/(*n*_case_ + *n*_control_). The Long COVID HGI is a global and ongoing collaboration, open to all studies around the world that have data to run long COVID GWAS using our phenotypic criteria described below.

### Phenotype definitions

We used the following criteria for assigning case–control status for long COVID aligning with the World Health Organization guidelines^1^ (Supplementary Note; https://github.com/long-covid-hg/LongCovidTools/blob/main/PhenotypeDefinitions_LongCOVID_v1.docx). Study participants were defined as long COVID cases if, at least three months since SARS-CoV-2 infection or COVID-19 onset, they met any of the following criteria:

1. Presence of one or more self-reported COVID-19 symptoms that cannot be explained by an alternative diagnosis
2. Report of ongoing substantial impact on day-to-day activities
3. Any diagnosis codes of long COVID (for example, post-COVID-19 condition, ICD-10 code U09(.9))

Criteria 1 and 2 were applied only to questionnaire-based cohorts, whereas 3 was used in studies with electronic health records (EHR). Detailed phenotyping criteria and diagnosis codes of each study are provided in Supplementary Table 12.

We used two long COVID case definitions, a strict definition requiring a test-verified SARS-CoV-2 infection and a broad definition including self-reported or clinician-diagnosed SARS-CoV-2 infection (any long COVID).

We applied two control definitions. First, we used population controls, that is, everybody that is not the case. Population controls were genetic ancestry-matched individuals who were not defined as long COVID cases using the above-mentioned questionnaire or EHR-based definition. In the second analysis, we compared long COVID cases to individuals who had had SARS-CoV-2 infection but who did not meet the criteria of long COVID, that is, had fully recovered within three months from the infection.

We used in total four different case–control definitions to generate four GWASs as below:

1. Long COVID cases after test-verified SARS-CoV-2 infection versus population controls (the strict case definition versus the broad control definition)
2. Long COVID within test-verified SARS-CoV-2 infection (the strict case definition versus the strict control definition)
3. Any long COVID cases versus population controls (the broad case definition versus the broad control definition)
4. Long COVID within any SARS-CoV-2 infection (the broad case definition versus the strict control definition)

To further investigate the effect of *FOXP4* locus on the different manifestations of long COVID^7^ in the FinnGen and MVP datasets, we used combined criteria of any long COVID diagnosis (BB: ICD-10 diagnosis code: U09* (where * can be empty or any string, referring to subdiagnoses)) with lifetime occurrence of specific symptom diagnoses: diabetes (ICD-10: E10*, E11*, E12*, E13*, E14*), fatigue and malaise (ICD-10: R53*, G93.3), asthma (ICD-10: J45*), skin paresthesia (ICD-10: R20.2), β-adrenergic inhalants (Anatomical Therapeutic Chemical (ATC) drug code: R03AC*), headache (ICD-10: R51*), proton pump inhibitors (ATC: A02BC*) or cardiac arrhythmia/abnormalities of heartbeat (ICD-10: I49*, R00*; Supplementary Fig. 13 and Supplementary Table 36). The effect of the risk variant rs9367106-C on long COVID with each symptom or medication was estimated separately using logistic regression, adjusting for age, sex and ten principal components. Finnish ancestry from FinnGen and African, Admixed American and European ancestries from the MVP were first analyzed separately, followed by a meta-analysis and test for heterogeneity.

### GWAS

We largely applied the GWAS analysis plans used in the COVID-19 HGI^6^. Each study performed its own sample collection, genotyping, genotype and sample quality control, imputation and association analyses independently, according to our central analysis plan (https://github.com/long-covid-hg/LongCovidTools/blob/main/COVID19HostGenetics_AnalysisPlan_LongCOVID_v1.docx), before submitting the GWAS summary statistic level results for meta-analysis (details are provided in Supplementary Table 12). We recommended that GWASs were run using REGENIE^57^ on chromosomes 1–22 and X, although a minority of the contributing studies used SAIGE^58^ or PLINK2 (ref. ^59^; Supplementary Table 12). The minimum set of covariates to be included at runtime were age, age^2^, sex, age × sex and the first ten genetic principal components. We advised studies to include any additional study-specific covariates where needed, such as those related to genotype batches or other demographic and technical factors that could lead to stratification within the cohort. Studies (*n* = 2) performing the GWAS using software that does not account for sample relatedness (such as PLINK) were advised to exclude related individuals.

### GWAS meta-analyses

The meta-analysis pipeline was also adopted from the COVID-19 HGI flagship paper^16^. The code is available at Long COVID HGI GitHub (https://github.com/long-covid-hg/META_ANALYSIS/) and is a modified version of the pipeline developed for the COVID-19 HGI (https://github.com/covid19-hg/META_ANALYSIS). To ensure that individual study results did not suffer from excessive inflation, deflation and false positives, we manually investigated plots of the reported allele frequencies against aggregated gnomAD v3.0 (ref. ^55^) allele frequencies in the same population. We also evaluated whether the association standard errors were excessively small, given the calculated effective sample size, to identify studies deviating from the expected trend. Where these issues were detected, the studies were contacted to reperform the association analysis, if needed, and resubmit their results.

Before the meta-analysis itself, the summary statistics were standardized, filtered (excluding variants with allele frequency <0.1% or imputation INFO score <0.6), lifted over to reference genome build GRCh38 (in studies imputed to GRCh37) and harmonized to gnomAD v3.0 through matching by chromosome, position and alleles (Supplementary Note).

The meta-analysis was performed using a fixed-effects IVW method on variants that were present in at least two studies contributing to the specific phenotype being analyzed. To assess whether one study was primarily driving any associations, we simultaneously ran a leave-most-significant-study-out (LMSSO) meta-analysis for each variant (based on the variant’s study-level *P* value). Heterogeneity between studies was estimated using Cochran’s *Q* test^60^. Each set of meta-analysis results was then filtered to exclude variants whose total effective sample size (in the non-LMSSO analysis) was less than one-third of the total effective sample size of all studies contributing to that meta-analysis. We report significant loci that pass the genome-wide significance threshold (*P* ≤ 5 × 10^−8^/4 = 1.25 × 10^−^^8^) accounting for the number of GWAS meta-analyses we performed.

### Principal component projection

In a similar fashion to the COVID-19 HGI, we asked each study to project their cohort onto a multiethnic genetic principal component space (Supplementary Fig. 5), by providing studies with precomputed PC loadings and reference allele frequencies from unrelated samples from the 1000 Genomes Project^20,21^ and the Human Genome Diversity Project. The loadings and frequencies were generated for a set of 117,221 autosomal, common (minor allele frequency (MAF) ≥ 0.1%) and LD-pruned (*r*^2^ < 0.8; 500-kb window) SNPs that would be available in the imputed data of most studies. Access to the projecting and plotting scripts was made available to the studies at https://github.com/long-covid-hg/pca_projection.

### eQTL, PheWAS and colocalization

For the single (Bonferroni-corrected) genome-wide significant lead variant, rs9367106, we used the GTEx portal (https://gtexportal.org/)^22,23^ to understand whether this variant had any tissue-specific effects on gene expression. As rs9367106 was not available in the GTEx database, we first identified a proxy variant, rs12660421 (*r*^2^ = 0.90) using all individuals from the 1000 Genomes Project^20,21^ and then performed a lookup in the portal’s GTEx v8 dataset^23^.

To identify other phenotypes associated with rs9367106, we used the Biobank Japan PheWeb portal (https://pheweb.jp/)^9^ to perform a phenome-wide association analysis, as the MAF of rs9367106 is highest in East Asia. Furthermore, we explored variant and locus-level associations in Estonian Biobank, FinnGen and Open Targets.

To assess whether the *FOXP4* association is shared between long COVID, and tissue-specific eQTLs, lung cancer and COVID-19 hospitalization, we extracted a 1-Mb region centered on rs9367107 (chr6: 41,015,652–42,015,652) from the lung cancer and COVID-19 hospitalization summary statistics and the GTEx v8 data and performed colocalization analyses using the R package coloc (v5.1.0.1)^61,62^ in R v4.2.2. Colocalization locus zoom plots were created using the LocusCompareR R package v1.0.0 (ref. ^63^), with LD *r*^2^ estimated using 1000 Genomes European-ancestry individuals^20,21^.

### Genetic correlation and MR

We assessed the genetic overlap and causal associations between long COVID outcomes and the same set of risk factors, biomarkers and disease liabilities as in the COVID-19 HGI flagship paper^16^. Additionally, we tested the overlap and causal impact of COVID-19 susceptibility and hospitalization risk. Genetic correlations were assessed using Linkage Disequilibrium Score Regression v1.0.1 (ref. ^64^). Where there were sufficient genome-wide significant variants, the causal impact was tested in a two-sample MR framework using the TwoSampleMR (v0.5.6) R package^65^ with R v4.0.3. To avoid sample overlap between exposure GWASs (here COVID-19 hospitalization and SARS-CoV-2 reported infection) and outcome GWASs (here long COVID phenotypes), we performed meta-analyses of COVID-19 hospitalization and SARS-CoV-2 reported infection using data freeze 7 of the COVID-19 HGI by excluding studies that participated in the long COVID (data freeze 4) effort. Independent significant exposure variants with *P* ≤ 5 × 10^−^^8^ were identified by LD-clumping the full set of summary statistics using an LD *r*^2^ threshold of 0.001 (based on the 1000 Genomes European-ancestry reference samples^20,21^) and a 10-Mb clumping window. For each exposure–outcome pair, these variants were then harmonized to remove variants with mismatched alleles and ambiguous palindromic variants (MAF > 45%). Fixed-effects IVW meta-analysis was used as the primary MR method, with MR–Egger, weighted median estimator, weighted mode-based estimator and MR-PRESSO used in sensitivity analyses. Heterogeneity was assessed using the MR-PRESSO global test and pleiotropy using the MR–Egger intercept. The genetic correlation and MR analyses were implemented as a Snakemake Workflow made available at https://github.com/marcoralab/MRcovid. Leave-one-variant-out-MR and European-only long COVID analyses were run as sensitivity analyses to test the robustness of MR results with COVID hospitalization as exposure and long COVID as outcome.

Summaries of the exposure GWAS are provided in Supplementary Table 26, and the association statistics for all exposure variants are provided in Supplementary Data.

### Bayesian clustering of effects based on linear relationships

We compared effect size estimates between long COVID and COVID severity, and similarly, between long COVID and SARS-CoV-2 infection. COVID-19 hospitalization was used as a proxy for severity. For this purpose, we selected those variants that had earlier association evidence at the genome-wide significant level for COVID-19 severity or SARS-CoV-2 infection and examined whether these variants had joint or higher effect than expected for long COVID. The linemodels R package was utilized for comparing linear relationships (https://github.com/mjpirinen/linemodels)^66^. This line model method performs probabilistic clustering of variables based on their observed effect sizes on two outcomes (Supplementary Note).

### Statistics and reproducibility

To maximize the statistical power for detecting genetic variants associated with long COVID, we used data from as many cohorts as possible with information on long COVID and study participants without long COVID. Moreover, to ensure reproducibility, we examined the robustness and replication of the signal across nine independent cohorts that joined the Long COVID HGI after data freeze 4 where the association was initially discovered.

For additional methodological details, see Supplementary Note.

### Reporting summary

Further information on research design is available in the Nature Portfolio Reporting Summary linked to this article.

## Online content

Any methods, additional references, Nature Portfolio reporting summaries, source data, extended data, supplementary information, acknowledgements, peer review information; details of author contributions and competing interests; and statements of data and code availability are available at 10.1038/s41588-025-02100-w.

## Supplementary information

Supplementary Information Supplementary Figs. 1–13 and Supplementary Note (Supplementary Methods and Acknowledgements).

Reporting Summary

Supplementary Tables Supplementary Tables 1–36.

Supplementary Data Harmonized association statistics for MR exposures and outcomes.

## Data Availability

We have made the results of these GWAS meta-analyses publicly available for variants passing post-meta-analysis filtering for MAF ≥ 1% and effective sample size >1/3 of the maximum effective sample size for each meta-analysis. The results from the four meta-analyses have been deposited to GWAS Catalog^67^ and LocusZoom^68^, where the associations can be visually explored and the summary statistics exported for further scientific discovery. Strict case definition (long COVID after test-verified SARS-CoV-2 infection) versus broad control definition (population control): https://www.ebi.ac.uk/gwas/studies/GCST90454540 https://my.locuszoom.org/gwas/192226/ Broad case definition (long COVID after any SARS-CoV-2 infection) versus broad control definition: https://www.ebi.ac.uk/gwas/studies/GCST90454541 https://my.locuszoom.org/gwas/826733/ Strict case definition versus strict control definition (individuals that had SARS-CoV-2 but did not develop long COVID): https://www.ebi.ac.uk/gwas/studies/GCST90454542 https://my.locuszoom.org/gwas/793752/ Broad case definition versus strict control definition: https://www.ebi.ac.uk/gwas/studies/GCST90454543 https://my.locuszoom.org/gwas/91854/

## Code availability

Instructions and example code for phenotyping, sample collection, genotyping, genotype and sample quality control, imputation and association analyses are shared in our central analysis plan (https://github.com/long-covid-hg/LongCovidTools/blob/main/COVID19HostGenetics_AnalysisPlan_LongCOVID_v1.docx, https://github.com/long-covid-hg/LongCovidTools/blob/main/PhenotypeDefinitions_LongCOVID_v1.docx). Furthermore, we have used GitHub public repositories for providing code for GWAS summary statistics lift-over and meta-analyses (https://github.com/long-covid-hg/META_ANALYSIS, modified from the previously published COVID-19 HGI pipeline^15,16^), for PCA projecting and plotting (https://github.com/long-covid-hg/pca_projection) and for MR and genetic correlation (https://github.com/marcoralab/MRcovid). Code used for fine mapping (https://github.com/mkanai/slalom)^27^ and Bayesian clustering of effects based on linear relationships (https://github.com/mjpirinen/linemodels)^66^ is also publicly available and has been previously published.
