# Supplementary material for: Genome-wide association study of long COVID
Source: Nat Genet. 2025 May 21;57(6):1402–17. doi: 10.1038/s41588-025-02100-w (PMC12165857; doi:10.1038/s41588-025-02100-w)
Supplement: Supplementary file 1 — Supplementary Figs. 1–13 and Supplementary Note (Supplementary Methods and Acknowledgements). [file 41588_2025_2100_MOESM1_ESM.pdf]

nature genetics

Supplementary information https://doi.org/10.1038/s41588-025-02100-w

Genome-wide association study of long COVID

In the format provided by the authors and unedited

Supplementary Information

Supplementary Figures **2**

Supplementary Figure 1. Frequency of Long COVID symptoms. 2

Supplementary Figure 2. Manhattan plots of each of the four Long COVID GWAS meta-analyses. 3

Supplementary Figure 3. Chromosome 6 lead variant across the contributing studies and ancestries in discovery and replication GWAS meta-analyses. 5

Supplementary Figure 4. Minor allele frequency of lead variant across ancestries. 7

Supplementary Figure 5. Principal component (PC) projection. 8

Supplementary Figure 6. Expression quantitative trait loci (eQTL) across tissues. 9

Supplementary Figure 7. Colocalization analyses of Long COVID with *FOXP4* eQTL, lung cancer, and COVID-19 hospitalization. 10

Supplementary Figure 8. Phenome-wide association study (PheWAS) of the lead variant. 11

Supplementary Figure 9. *FOXP4* expression in blood associates with Long COVID. 12

Supplementary Figure 10. Single cell analysis of *FOXP4* expression in lung COVID-19 autopsy donor samples. 13

Supplementary Figure 11. Fine-mapping with SLALOM. 14

Supplementary Figure 12. Cumulative Long COVID cases by *FOXP4* genotype. 14

Supplementary Figure 13. *FOXP4* variant effect on subtypes of Long COVID in FinnGen and VA Million Veteran Program. 15

References for Supplementary Figures 16

Supplementary Note: Methods **17**

Long COVID Host Genetics Initiative - Inclusion & Ethics 17

Phenotype definitions 17

Codes to extract cases using registry or electronic health record data 17

Strict and broad phenotype definitions 18

Cohort ancestry and description 19

Data harmonization 19

GWAS meta-analyses 20

Expression quantitative trait loci (eQTL) 20

Colocalization 20

Cell-type specific *FOXP4* expression 20

RNA sequencing in the BQC19 21

Enhancers, transcription factor binding sites, and active chromatin regions 21

Phenome-wide association study (PheWAS) 22

Mendelian Randomisation 22

Genetic correlation 24

Fine-mapping 24

Bayesian clustering of effects based on linear relationships 24

Stratified and adjusted analyses for strain, vaccination, and severity 25

Cox proportional hazard and recessive model 26

References for Supplementary Methods 26

Supplementary Note: Acknowledgements **28**

Supplementary Figures

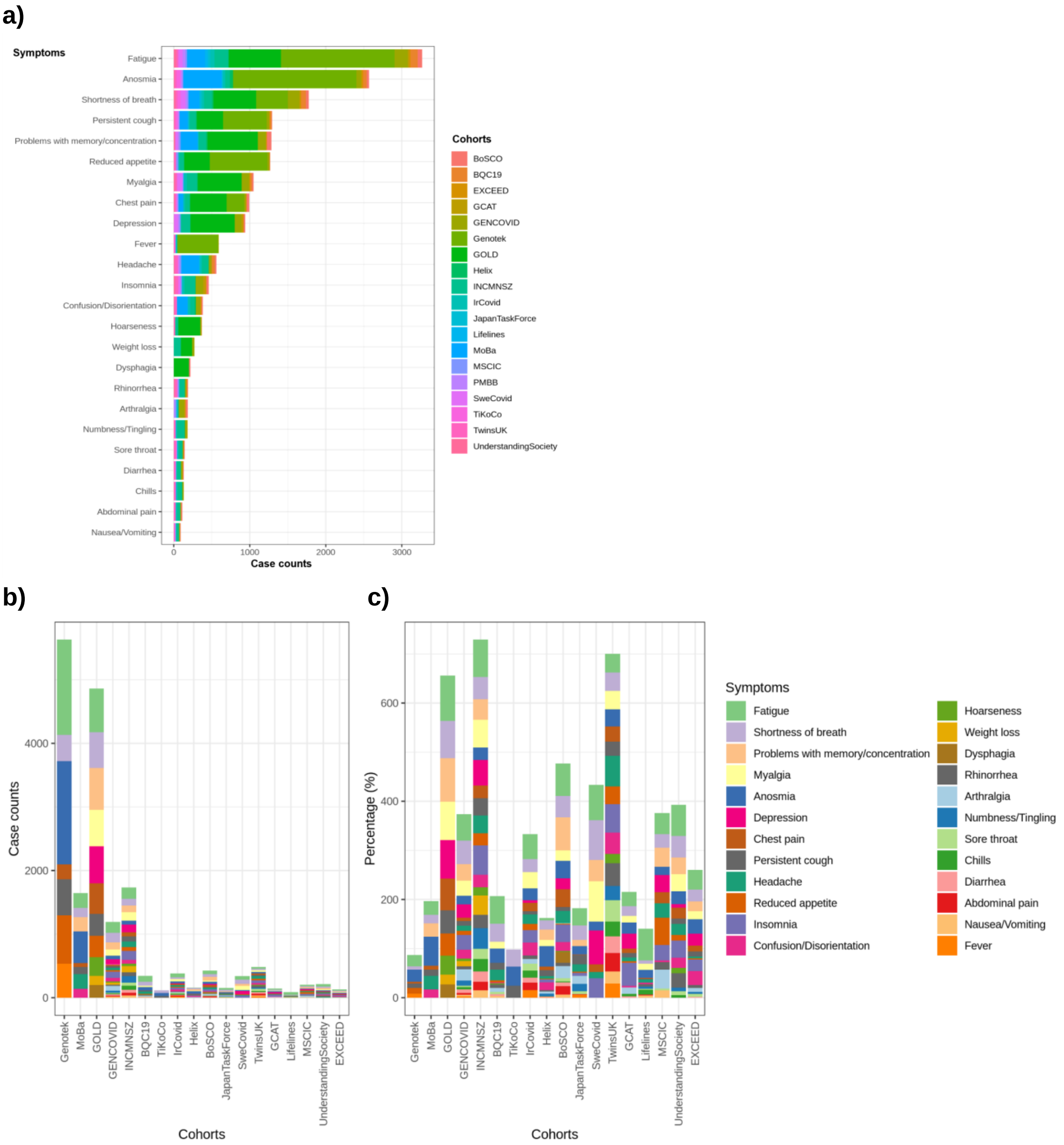

Supplementary Figure 1. Frequency of Long COVID symptoms.

a) Total case counts per each symptom, with contributing cohorts separated by colours.
b) Case counts and **c)** percentages of cases with each symptom, stratified by cohorts. (Note that an individual may have several symptoms, thus the percentages do not total into 100%.) [INCMNSZ = MexGen-COVID Initiative]

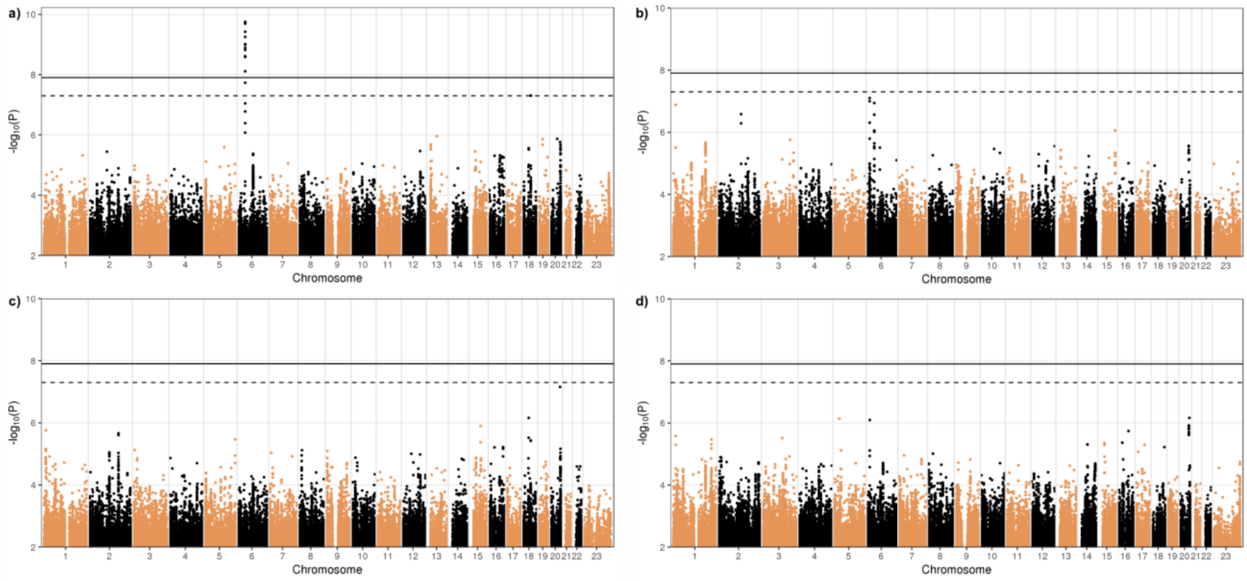

Supplementary Figure 2. Manhattan plots of each of the four Long COVID GWAS meta-analyses.

Manhattan plots of **a)** Long COVID after test-verified SARS-CoV-2 infection (strict case definition, N = 3,018) compared to all other individuals in each data set (population controls, broad control definition, N = 994,582), **b)** Long COVID after any (test-verified, physician-diagnosed, or self-report) SARS-CoV-2 infection (broad case definition, N = 6,450) compared to population controls (broad control definition, N = 1,093,995), **c)** Long COVID after test-verified SARS-CoV-2 infection (strict case definition, N = 3,018) compared to those recovered within three months after test-verified SARS-CoV-2 infection (strict control definition, N = 37,935), and **d)** Long COVID after any (test-verified, doctor-diagnosed or self-report) SARS-CoV-2 infection (broad case definition, N = 6,450) compared to those recovered within three months after any SARS-CoV-2 infection (strict control definition, N= 46,208). A genome-wide significant association with Long COVID (strict case and broad control definition) was found in the chromosome 6, upstream of the *FOXP4* gene (chr6:41515652:G:C, GRCh38, rs9367106, as the lead variant; $P = 1.76\times10^{-10}$, Bonferroni $P = 7.06\times10^{-10}$, increased risk with the C allele, OR = 1.63, 95% CI: 1.40-1.89). Horizontal lines indicate genome-wide significant thresholds for inverse variance-weighted meta-analyses before ($P < 5\times10^{-8}$, dashed line) and after ($P < 1.25\times10^{-8}$, solid line) Bonferroni correction over the four Long COVID meta-analyses.

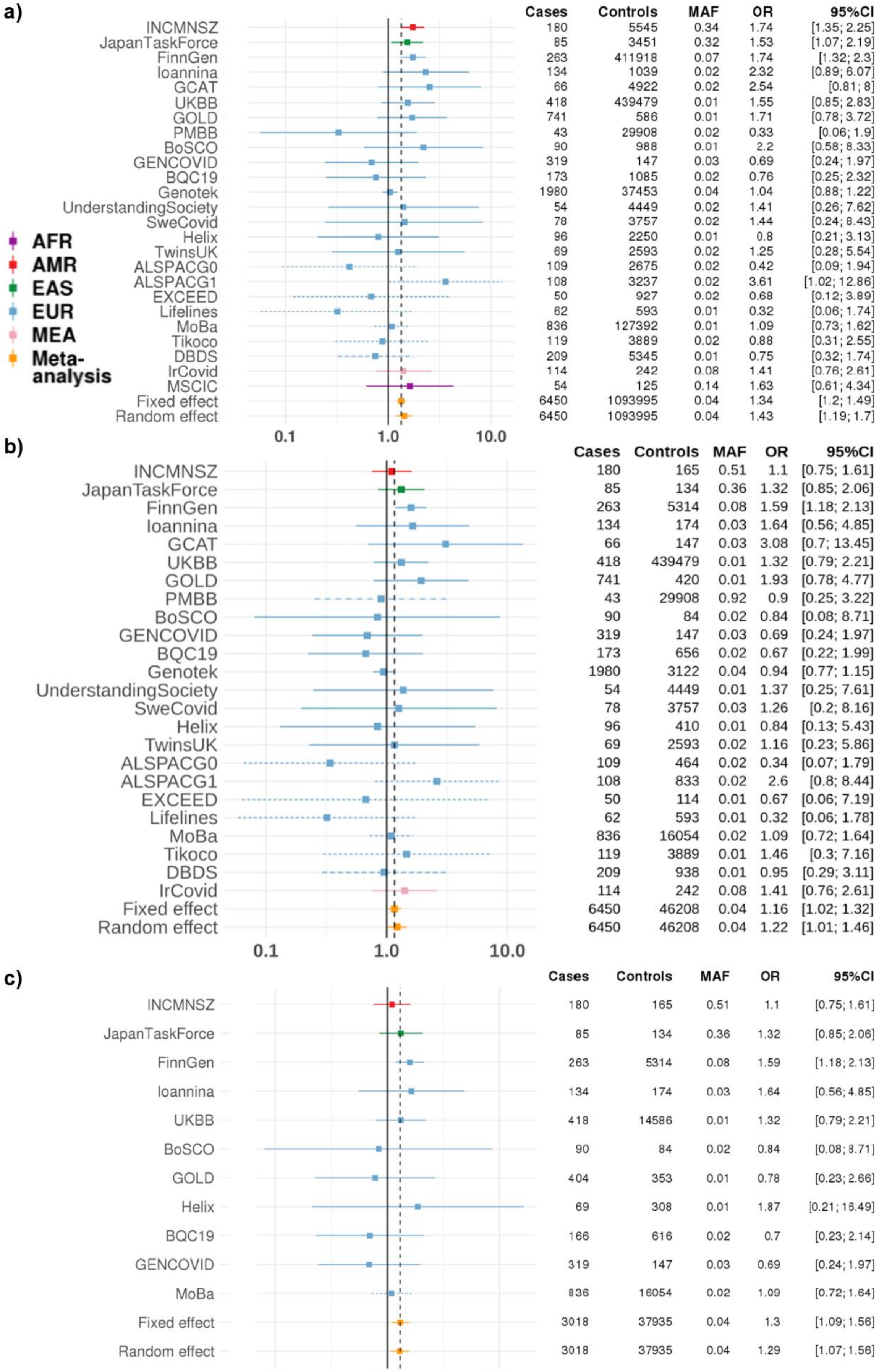
a)
AFR
AMR
EAS
EUR
MEA
Meta-analysis
Cases Controls MAF OR 95%CI
INCMNSZ 180 5545 0.34 1.74 [1.35; 2.25]
JapanTaskForce 85 3451 0.32 1.53 [1.07; 2.19]
FinnGen 263 411918 0.07 1.74 [1.32; 2.3]
Ioannina 134 1039 0.02 2.32 [0.89; 6.07]
GCAT 66 4922 0.02 2.54 [0.81; 8]
UKBB 418 439479 0.01 1.55 [0.85; 2.83]
GOLD 741 586 0.01 1.71 [0.78; 3.72]
PMBB 43 29908 0.02 0.33 [0.06; 1.9]
BoSCO 90 988 0.01 2.2 [0.58; 8.33]
GENCOVID 319 147 0.03 0.69 [0.24; 1.97]
BQC19 173 1085 0.02 0.76 [0.25; 2.32]
Genotek 1980 37453 0.04 1.04 [0.88; 1.22]
UnderstandingSociety 54 4449 0.02 1.41 [0.26; 7.62]
SweCovid 78 3757 0.02 1.44 [0.24; 8.43]
Helix 96 2250 0.01 0.8 [0.21; 3.13]
TwinsUK 69 2593 0.02 1.25 [0.28; 5.54]
ALSPACG0 109 2675 0.02 0.42 [0.09; 1.94]
ALSPACG1 108 3237 0.02 3.61 [1.02; 12.86]
EXCEED 50 927 0.02 0.68 [0.12; 3.89]
Lifelines 62 593 0.01 0.32 [0.06; 1.74]
MoBa 836 127392 0.01 1.09 [0.73; 1.62]
Tikoco 119 3889 0.02 0.88 [0.31; 2.55]
DBDS 209 5345 0.01 0.75 [0.32; 1.74]
IrCovid 114 242 0.08 1.41 [0.76; 2.61]
MSCIC 54 125 0.14 1.63 [0.61; 4.34]
Fixed effect 6450 1093995 0.04 1.34 [1.2; 1.49]
Random effect 6450 1093995 0.04 1.43 [1.19; 1.7]
0.1 1.0 10.0
b)
Cases Controls MAF OR 95%CI
INCMNSZ 180 165 0.51 1.1 [0.75; 1.61]
JapanTaskForce 85 134 0.36 1.32 [0.85; 2.06]
FinnGen 263 5314 0.08 1.59 [1.18; 2.13]
Ioannina 134 174 0.03 1.64 [0.56; 4.85]
GCAT 66 147 0.03 3.08 [0.7; 13.45]
UKBB 418 439479 0.01 1.32 [0.79; 2.21]
GOLD 741 420 0.01 1.93 [0.78; 4.77]
PMBB 43 29908 0.92 0.9 [0.25; 3.22]
BoSCO 90 84 0.02 0.84 [0.08; 8.71]
GENCOVID 319 147 0.03 0.69 [0.24; 1.97]
BQC19 173 656 0.02 0.67 [0.22; 1.99]
Genotek 1980 3122 0.04 0.94 [0.77; 1.15]
UnderstandingSociety 54 4449 0.01 1.37 [0.25; 7.61]
SweCovid 78 3757 0.03 1.26 [0.2; 8.16]
Helix 96 410 0.01 0.84 [0.13; 5.43]
TwinsUK 69 2593 0.02 1.16 [0.23; 5.86]
ALSPACG0 109 464 0.02 0.34 [0.07; 1.79]
ALSPACG1 108 833 0.02 2.6 [0.8; 8.44]
EXCEED 50 114 0.01 0.67 [0.06; 7.19]
Lifelines 62 593 0.01 0.32 [0.06; 1.78]
MoBa 836 16054 0.02 1.09 [0.72; 1.64]
Tikoco 119 3889 0.01 1.46 [0.3; 7.16]
DBDS 209 938 0.01 0.95 [0.29; 3.11]
IrCovid 114 242 0.08 1.41 [0.76; 2.61]
Fixed effect 6450 46208 0.04 1.16 [1.02; 1.32]
Random effect 6450 46208 0.04 1.22 [1.01; 1.46]
0.1 1.0 10.0
c)
Cases Controls MAF OR 95%CI
INCMNSZ 180 165 0.51 1.1 [0.75; 1.61]
JapanTaskForce 85 134 0.36 1.32 [0.85; 2.06]
FinnGen 263 5314 0.08 1.59 [1.18; 2.13]
Ioannina 134 174 0.03 1.64 [0.56; 4.85]
UKBB 418 14586 0.01 1.32 [0.79; 2.21]
BoSCO 90 84 0.02 0.84 [0.08; 8.71]
GOLD 404 353 0.01 0.78 [0.23; 2.66]
Helix 69 308 0.01 1.87 [0.21; 16.49]
BQC19 166 616 0.02 0.7 [0.23; 2.14]
GENCOVID 319 147 0.03 0.69 [0.24; 1.97]
MoBa 836 16054 0.02 1.09 [0.72; 1.64]
Fixed effect 3018 37935 0.04 1.3 [1.09; 1.56]
Random effect 3018 37935 0.04 1.29 [1.07; 1.56]
0.1 1.0 10.0

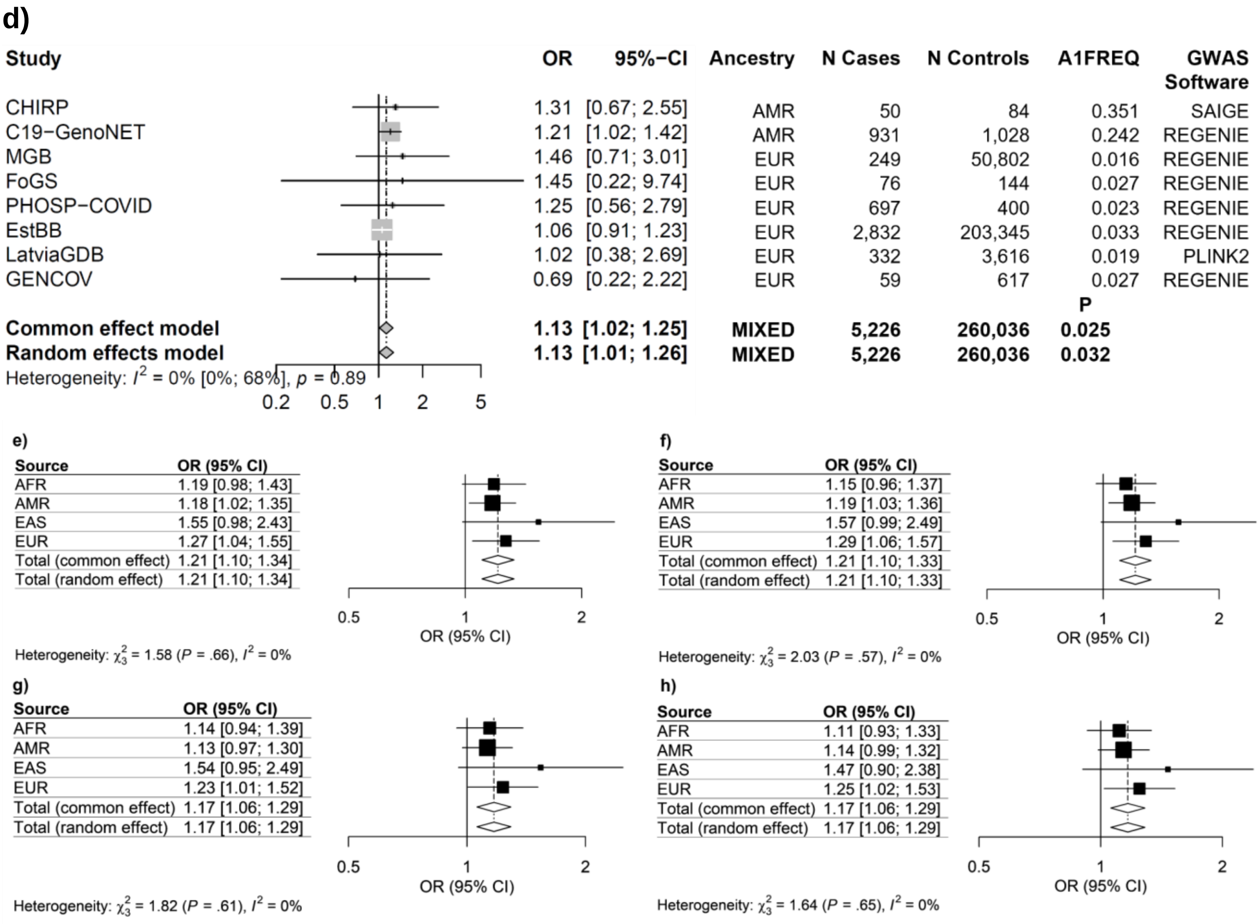

Supplementary Figure 3. Chromosome 6 lead variant across the contributing studies and ancestries in discovery and replication GWAS meta-analyses.

P values from inverse variance-weighted meta-analyses with common i.e. fixed-effect and random effect models, using metagen function from meta package v6 in R v4.3.0 (weight of each sample shown as box size). Horizontal lines: Center = odds ratio (OR); error bars = 95% confidence interval (CI). Vertical lines: Solid line, at OR = 1 i.e. no effect of variant with Long COVID to either direction; dashed line = meta-analyzed effect size (OR).

a,b,c) Long COVID lead variant from the Long COVID Host Genetics Initiative data freeze 4 meta-analysis with additional case and control definitions (see **Fig. 2**). If lead variant rs9367106 (solid line) was missing from the dataset, we imputed the plot by the variant with the highest linkage disequilibrium (LD) correlation coefficient (r) with the lead variant for illustrative purposes. Dotted line: rs12660421 (r = 0.98 in European in 1000G+HGDP samples[1]), Long-dashed line: rs1886814 (r = 0.65, for one cohort [DBDS]), Dashed line: rs1886817 (r = 0.52 for one cohort (PMBB) in **c**)). For the imputed variants, beta was weighted by multiplying by the LD correlation coefficient (r = 0.98 or 0.65). Minor allele frequency (MAF) varies across ancestries: Finnish European (FIN),

African (AFR), Admixed American (AMR), East Asian (EAS), European (EUR), Middle Eastern (MEA). **a)** Long COVID with broad case and broad control definition. **b)** Long COVID with broad case and strict control definition. **c)** Long COVID with strict case and strict control definition.

d) Replication of the association of Long COVID by strict case and broad control definition with rs12660421 across eight independent cohorts (for cohort details, see **Supplementary Table 12**) that did not participate in the initial discovery GWAS (**Fig. 2**). Risk allele frequency (A1FREQ) varies from 1.6% to 2.7% across cohorts with EUR ancestry (Mass General Brigham Biobank (MGB), Fondazione Genomics SARS-CoV-2 Study (FoGS), The Post-hospitalisation COVID-19 study (PHOSP-COVID), Estonian Biobank (EstBB), COVID-19 cohort at LGDB (LatviaGDB), GENCOV Study (GENCOV)) and from 24% to 35% within AMR ancestry (COVID-19 Genomics Network (C19-GenoNet), COVID-19 Host Immune Response Pathogenesis Study (CHIRP)).

e-h) Replication of the association of Long COVID with variants rs12660421 and rs9367106 across ancestries in VA Million Veteran Program data (imputed using 1000 Genomes Project + African Genome Resources reference panels). Strict case definition, Long COVID cases after test-verified SARS-CoV-2 infection, N = 4,274, of which AFR 791, AMR 741, EAS 47, and EUR 2,695.

e,f) Broad control definition (population controls, N = 538,799, of which 107,988 AFR, 47,151 AMR, 6,311 EAS, and 377,349 EUR); association replicated for rs12660421 (e) and rs9367106 (f), both P = 0.0001. **g,h)** Strict control definition (controls with test-verified SARS-CoV-2 infection but no Long COVID, N = 73,739, of which 16,414 AFR, 7,970 AMR, 849 EAS, and 48,506 EUR); association replicated for rs12660421 (g) and rs9367106 (h), both P = 0.0018.

[INCMNSZ = MexGen-COVID Initiative]

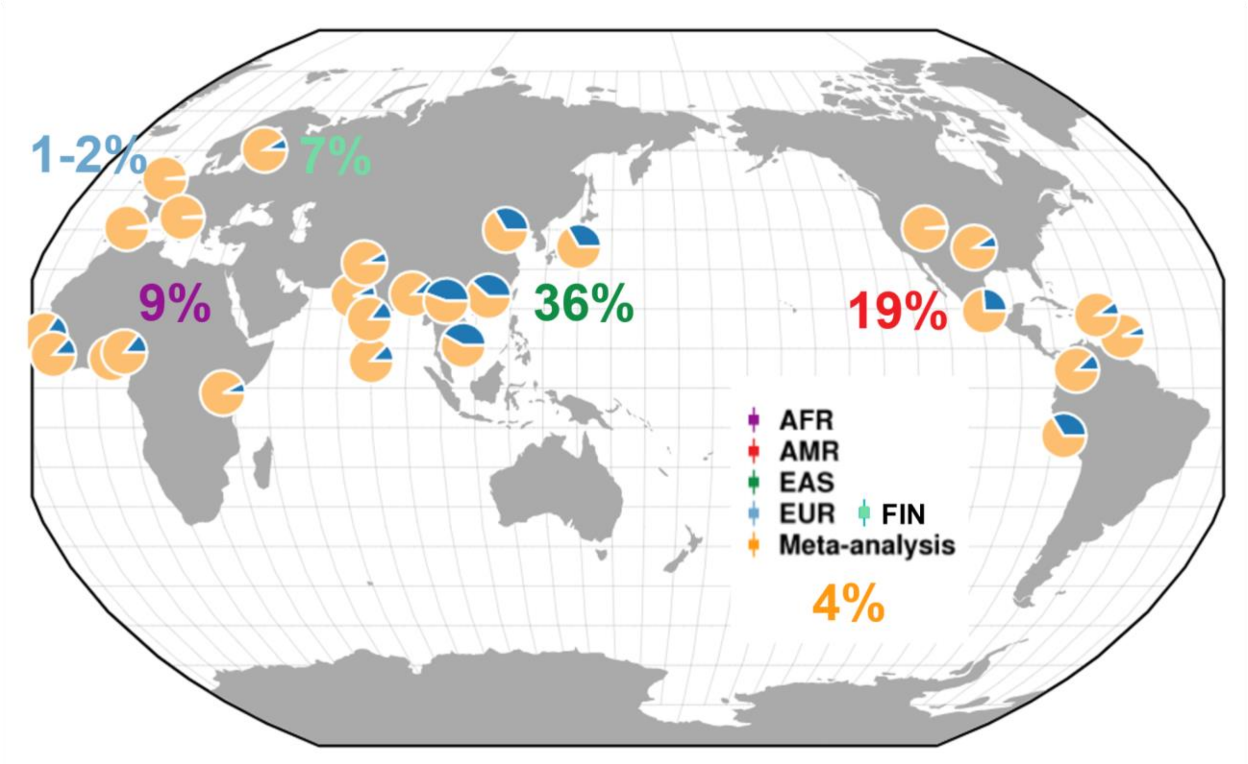

Supplementary Figure 4. Minor allele frequency of lead variant across ancestries.

Frequency of the Long COVID risk allele (rs9367106-C, marked with blue in pie charts; G allele in yellow) across different ancestries (Geography of Genetic Variants Browser, v0.4 beta, https://popgen.uchicago.edu/ggv/?data=%221000genomes%22&chr=6&pos=41483390)[2]. Long COVID risk allele frequency in populations included in our Long COVID meta-analyses (AFR = African, AMR = Admixed American, EAS = East Asian, EUR = European) marked with ancestry-coloured numbers (gnomAD v3.1.2, https://gnomad.broadinstitute.org/variant/6-41515652-G-C?dataset=gnomad_r3) [2,3].

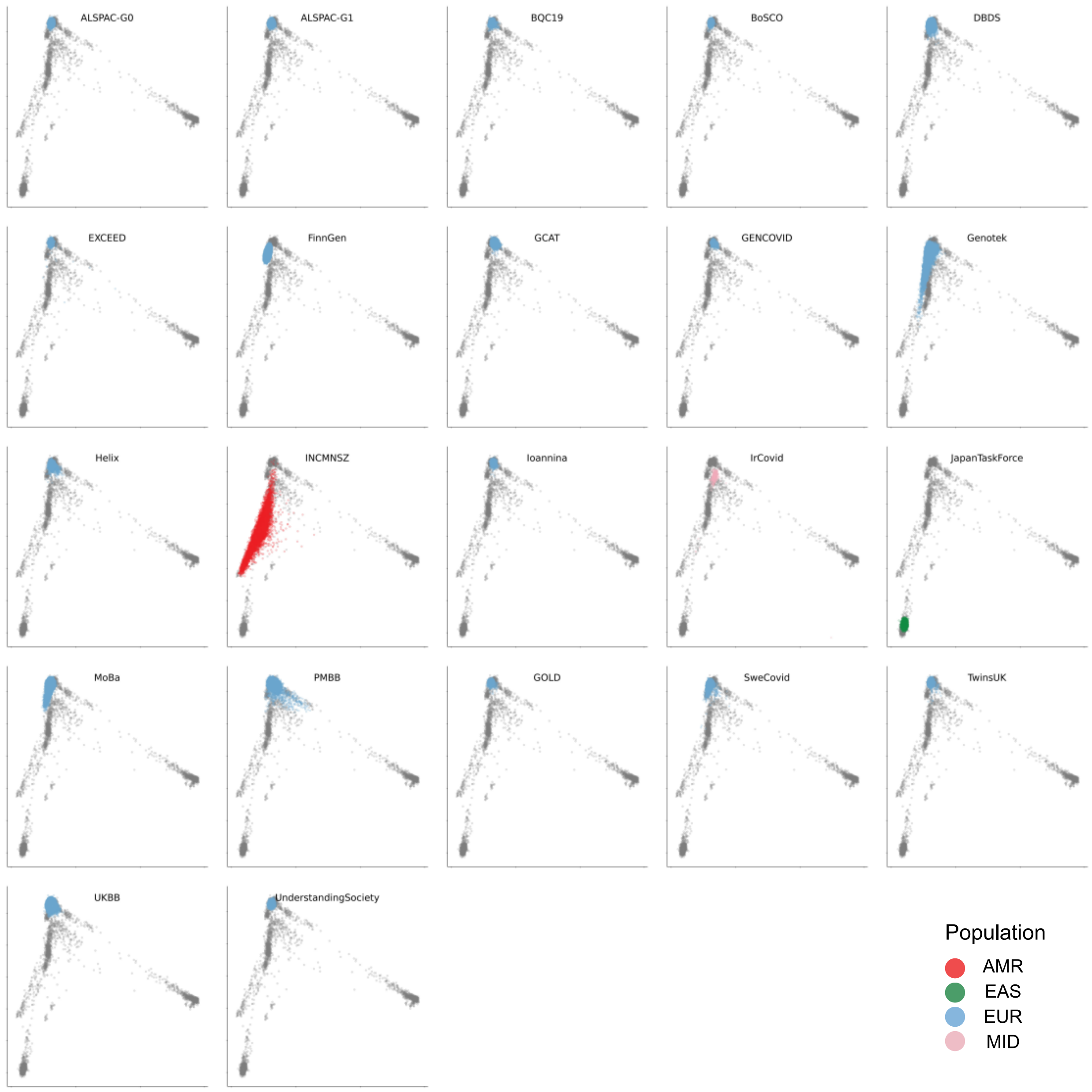

Supplementary Figure 5. Principal component (PC) projection.

Projection of 1000 Genomes genetic principal components 1 (x-axis) and 2 (y-axis) into studies contributing to the meta-analyses (see **Supplementary Table 11, 12**). Each study's samples are colored based on ancestry (Admixed American (AMR), East Asian (EAS), European (EUR), Middle-Eastern (MID)), with 1000 Genomes samples of all ancestries colored in grey. [INCMNSZ = MexGen-COVID Initiative]

Tissue	Samples	NES	p-value	m-value
Lung	515	0.558	5.3e-9	1.00
Brain - Hypothalamus	170	1.37	2.4e-6	0.919
Heart - Left Ventricle	386	0.220	0.02	0.733
Skin - Sun Exposed (Lower leg)	605	0.137	0.03	0.692
Cells - Cultured fibroblasts	483	0.145	0.04	0.657
Brain - Anterior cingulate cortex (BA24)	147	-0.482	0.09	0.283
Colon - Transverse	368	0.171	0.1	0.602
Esophagus - Mucosa	497	0.155	0.1	0.619
Thyroid	574	0.0869	0.1	0.522
Brain - Amygdala	129	-0.790	0.2	0.335
Pancreas	305	0.146	0.2	0.541
Liver	208	0.336	0.2	0.501
Brain - Nucleus accumbens (basal ganglia)	202	0.355	0.2	0.555
Muscle - Skeletal	706	-0.0862	0.2	0.0450
Uterus	129	0.449	0.2	0.510
Brain - Substantia nigra	114	0.416	0.2	0.536
Artery - Aorta	387	0.108	0.3	0.552
Brain - Spinal cord (cervical c-1)	126	0.347	0.3	0.453
Stomach	324	0.0869	0.3	0.502
Esophagus - Muscularis	465	0.0731	0.3	0.391
Pituitary	237	-0.182	0.3	0.267
Heart - Atrial Appendage	372	0.108	0.3	0.475
Spleen	227	0.144	0.3	0.459
Ovary	167	0.214	0.4	0.519
Adipose - Visceral (Omentum)	469	0.0595	0.4	0.405
Brain - Cerebellar Hemisphere	175	-0.187	0.4	0.277
Prostate	221	0.143	0.4	0.469
Cells - EBV-transformed lymphocytes	147	0.175	0.4	0.480
Colon - Sigmoid	318	0.0802	0.5	0.377
Brain - Cortex	205	-0.133	0.5	0.295
Minor Salivary Gland	144	0.0948	0.5	0.425
Artery - Tibial	584	0.0428	0.5	0.272
Adrenal Gland	233	0.112	0.5	0.462
Brain - Caudate (basal ganglia)	194	0.181	0.5	0.441
Breast - Mammary Tissue	396	-0.0524	0.5	0.182
Brain - Cerebellum	209	0.0866	0.6	0.425
Artery - Coronary	213	0.0601	0.6	0.429
Kidney - Cortex	73	-0.214	0.6	0.388
Brain - Hippocampus	165	0.104	0.7	0.422
Brain - Putamen (basal ganglia)	170	0.116	0.7	0.469
Brain - Frontal Cortex (BA9)	175	0.0968	0.7	0.469
Small Intestine - Terminal Ileum	174	0.0463	0.7	0.373
Skin - Not Sun Exposed (Suprapubic)	517	0.0224	0.7	0.184
Testis	322	-0.0284	0.8	0.189
Adipose - Subcutaneous	581	0.0182	0.8	0.224
Esophagus - Gastroesophageal Junction	330	-0.0221	0.8	0.248
Whole Blood	670	-0.00676	0.9	0.0480
Vagina	141	0.0297	0.9	0.416
Nerve - Tibial	532	-0.00427	1	0.245

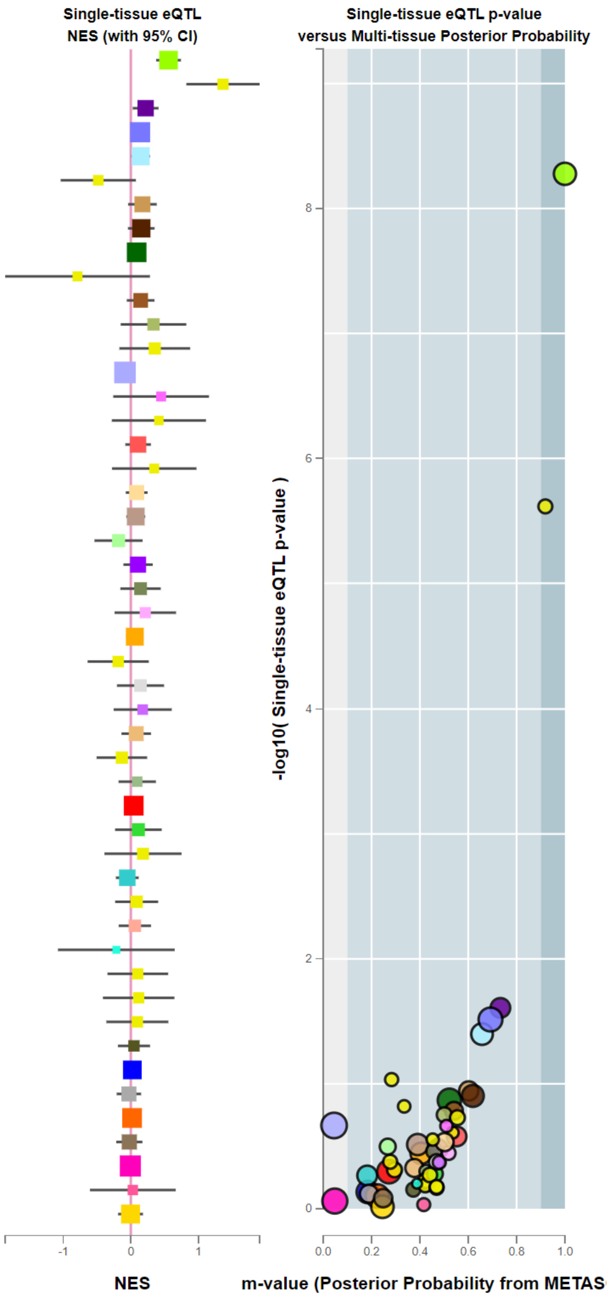

Supplementary Figure 6. Expression quantitative trait loci (eQTL) across tissues.

We show cross-tissue eQTL signals for rs12660421 to allow comparison of signals across tissues (https://gtexportal.org/home/snp/rs12660421). Tissues are sorted by eQTL P value. NES = normalized effect size. m-value = a posterior probability value for each variant-gene pair and tissue tested i.e. the probability that the eQTL effect exists in the given tissue, given the profile of eQTL effects across all investigated tissues (m-value ≥ 0.9 considered as significant)[4].

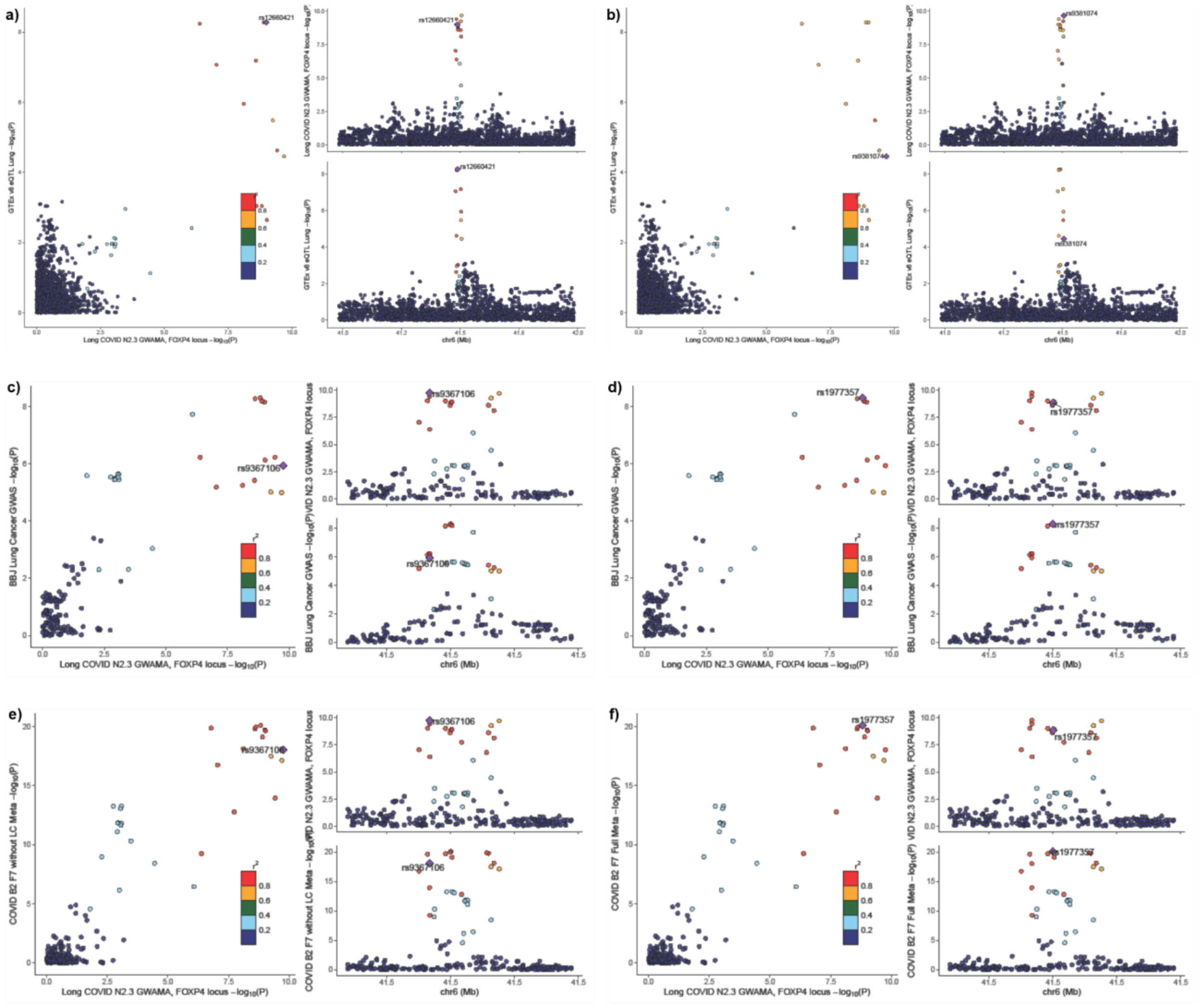

Supplementary Figure 7. Colocalization analyses of Long COVID with *FOXP4* eQTL, lung cancer, and COVID-19 hospitalization.

a-b) Long COVID association results with GTEx v8 *FOXP4* expression association results in lung tissue (posterior probability (pp) of shared association = 0.91) in the *FOXP4* locus. Colocalization analysis using eQTL data from GTEx v8 tissue type and Long COVID association data. Plots illustrate -$\log_{10}$ P-value for Long COVID (x-axis) and for *FOXP4* expression in the Lung (y-axis), regional association of the *FOXP4* locus variants with Long COVID, and regional association of the *FOXP4* variants with RNA expression measured in the lung in GTEx. Variants are coloured by 1000 Genomes European-ancestry LD r^2 with **a)** the lead variant (rs12660421) for *FOXP4* expression in lung tissue and **b)** variant (rs9381074) representing the most significant Long COVID variant overlapping the GTEx v8 dataset.
c-d) Long COVID association results and Biobank Japan lung cancer association results (pp = 0.98) in the *FOXP4* locus. Plots illustrate -$\log_{10}$ P-value for Long COVID (x-axis) and for lung cancer (y-axis), regional association of the *FOXP4* locus variants with Long COVID, and regional

association of the *FOXP4* variants with lung cancer. Variants are coloured by 1000 Genomes European-ancestry LD r^2 with **c)** the lead variant for Long COVID (rs9367106) and **d)** for lung cancer (rs1977357).

e-f) Long COVID association results and COVID-19 hospitalization association results (pp = 0.97) in the *FOXP4* locus. Plots illustrate $-\log_{10}$ P-value for Long COVID (x-axis) and for COVID-19 (y-axis), regional association of the *FOXP4* locus variants with Long COVID, and regional association of the *FOXP4* variants with COVID-19 hospitalization. Variants are coloured by 1000 Genomes European-ancestry LD r^2 with **e)** the lead variant (rs9367106) for Long COVID and **f)** for COVID-19 hospitalization (rs1977357).

(See also **Supplementary Table 17** for colocalization results.)

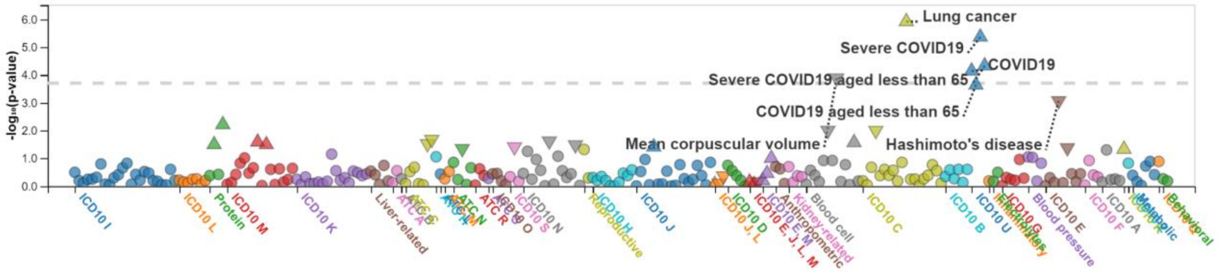

Supplementary Figure 8. Phenome-wide association study (PheWAS) of the lead variant.

Variant-level PheWAS analysis of the Long COVID lead variant rs9367106 (chr6:41515652:G:C, GRCh38) and all traits (N = 262) in Biobank Japan (https://pheweb.jp/variant/6:41483390-G-C). Significant associations with lung cancer and COVID-19 (P values above the dashed line significant after Bonferroni correction ($0.05/262 = 1.9\times10^{-4}$)). (See **Supplementary Table 18** for all associations with $P < 0.05$.)

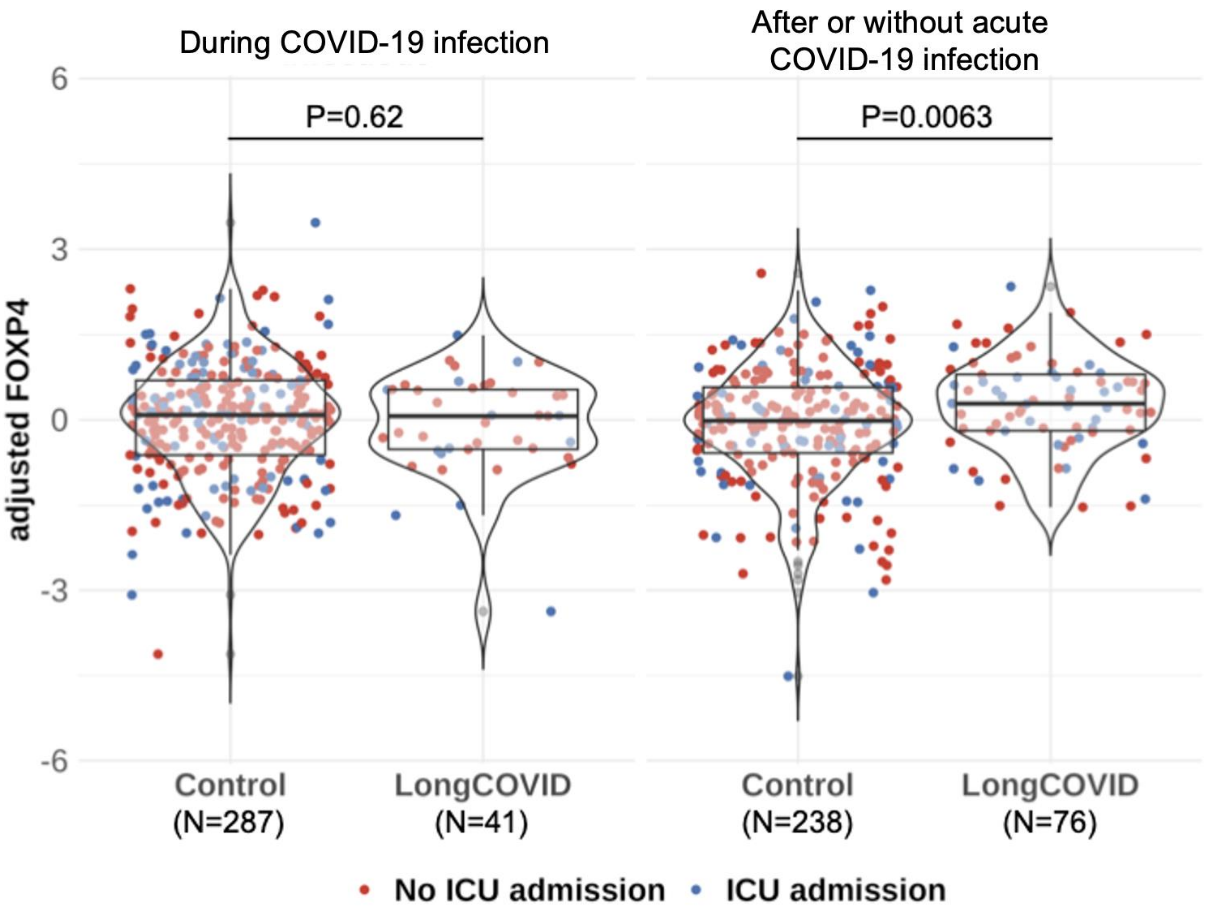

Supplementary Figure 9. *FOXP4* expression in blood associates with Long COVID.

Adjusted *FOXP4* RNA expression level in blood samples of acute and non-acute COVID-19 infection from individuals with and without Long COVID in the Biobanque québécoise de la COVID-19 (BQC19). We defined non-acute COVID-19 samples as those collected at least 31 days after onset of their symptoms from patients with SARS-CoV-2 infection or samples collected from patients negative for SARS-CoV-2 by PCR (N = 314). Furthermore, we defined acute COVID-19 samples as those collected within -2 to 14 days from symptom onset from patients positive for SARS-CoV-2 (N = 328). *FOXP4* level was adjusted for age, sex and ICU admission. P-values were obtained by logistic regression to assess the association of adjusted *FOXP4* with Long COVID. Lower edge of the whisker: the lowest value within 1.5 * IQR of the hinge, lower hinge: 25% quantile, horizontal line contained within the box: median value, upper hinge: 75% quantile, the upper edge of the whisker: the highest value that is within 1.5 * IQR of the hinge.

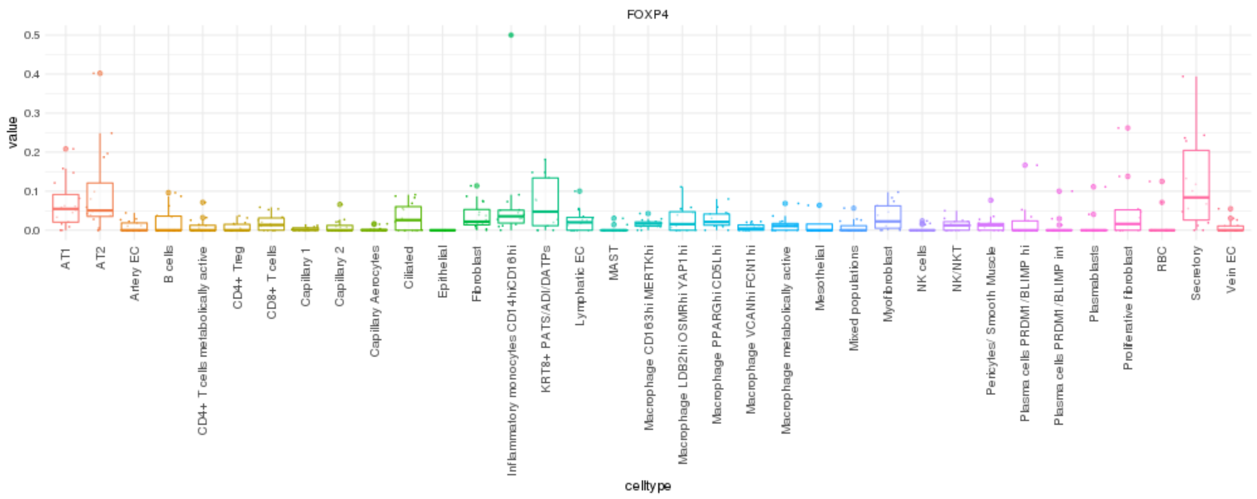

Supplementary Figure 10. Single cell analysis of *FOXP4* expression in lung COVID-19 autopsy donor samples.

Single-cell *FOXP4* expression of 23 lung COVID-19 autopsy donor tissue samples from GSE171668[5]. The mean value of *FOXP4* expression of the cells annotated in the same subcategory was represented as a dot per each sample. The cell type annotation was manually performed in the original publication. AT1 alveolar type 1 epithelial cells, AT2 alveolar type 2 epithelial cells, EC endothelial cells, KRT8+ PATS/ADI/DATPs KRT8+ pre-alveolar type 1 transitional cell state, MAST mast cells, RBC red blood cells. Lower edge of the whisker: the lowest value within 1.5 * IQR of the hinge, lower hinge: 25% quantile, horizontal line contained within the box: median value, upper hinge: 75% quantile, the upper edge of the whisker: the highest value that is within 1.5 * IQR of the hinge.

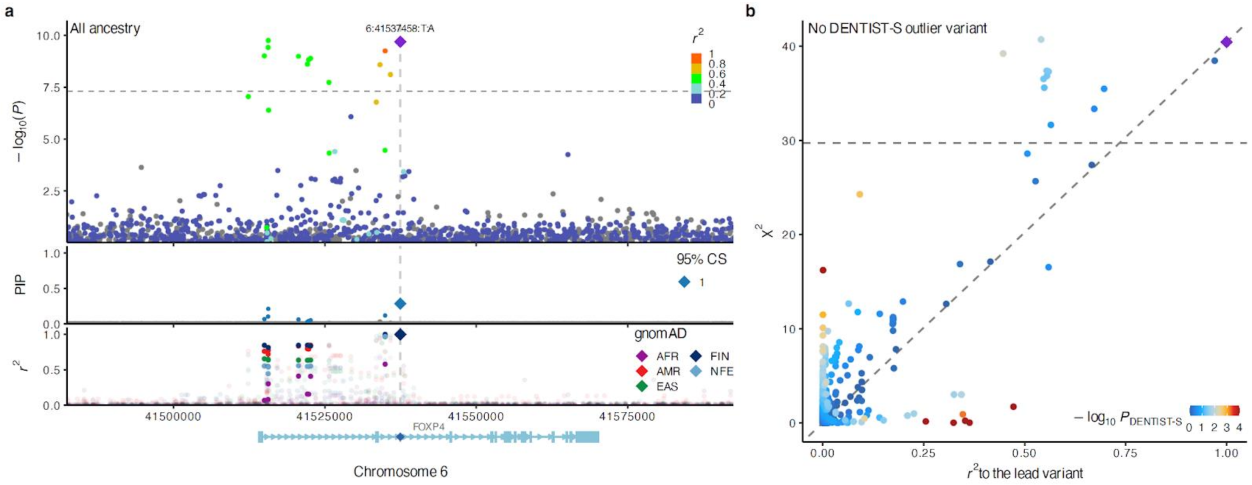

Supplementary Figure 11. Fine-mapping with SLALOM.

a) Fine-mapping using SLALOM[6] at the *FOXP4* locus indicates rs9381074 with the highest posterior probability for a causal variant. The figure shows posterior inclusion probability (PIP) in the middle and local LD by r^2 and ancestry groups at the bottom. **b)** A diagnosis plot using r^2 values to the lead variant versus marginal χ2. Colors show $-\log_{10}$ ($P_{\text{DENTIST-S}}$) values[6]. Outlier variants with $P_{\text{DENTIST-S}} < 10^{-4}$ are shown in red with a diamond shape.

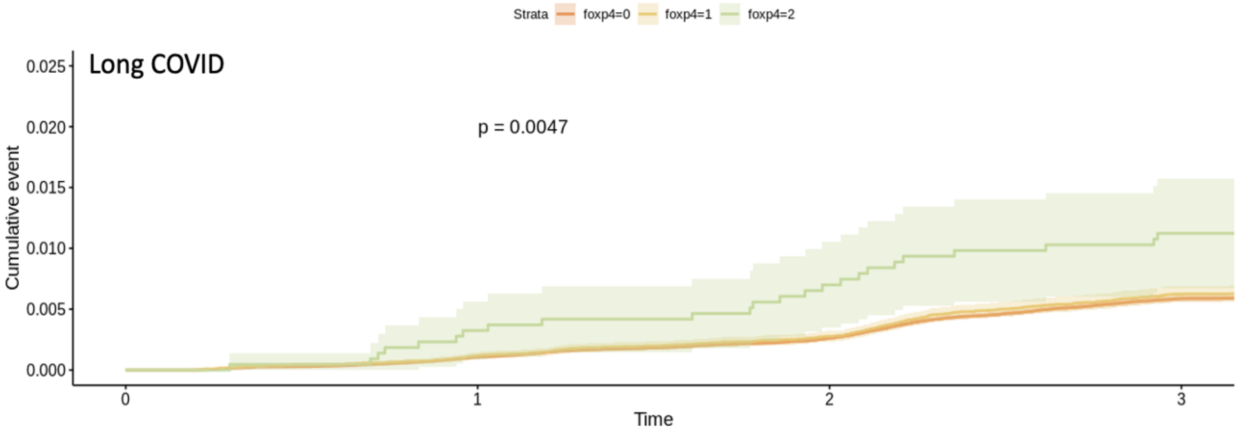

Supplementary Figure 12. Cumulative Long COVID cases by *FOXP4* genotype.

Analysis of Long COVID incidence over time since 2020. Time is in years since 2020, event is the date of COVID-19 infection, and Cox proportional hazard model is adjusted for age, sex and ten first principal components. Analysis was run in FinnGen with 3,684 individuals with Long COVID and 496,664 population controls (release 12; description of FinnGen study, earlier release, in [7]). The p-value was obtained through the log-rank test as part of the survminer package v0.4.9, and the 95% confidence interval of the Kaplan-Meier curve is depicted as the shaded areas. Risk genotype alt/alt is depicted in green.

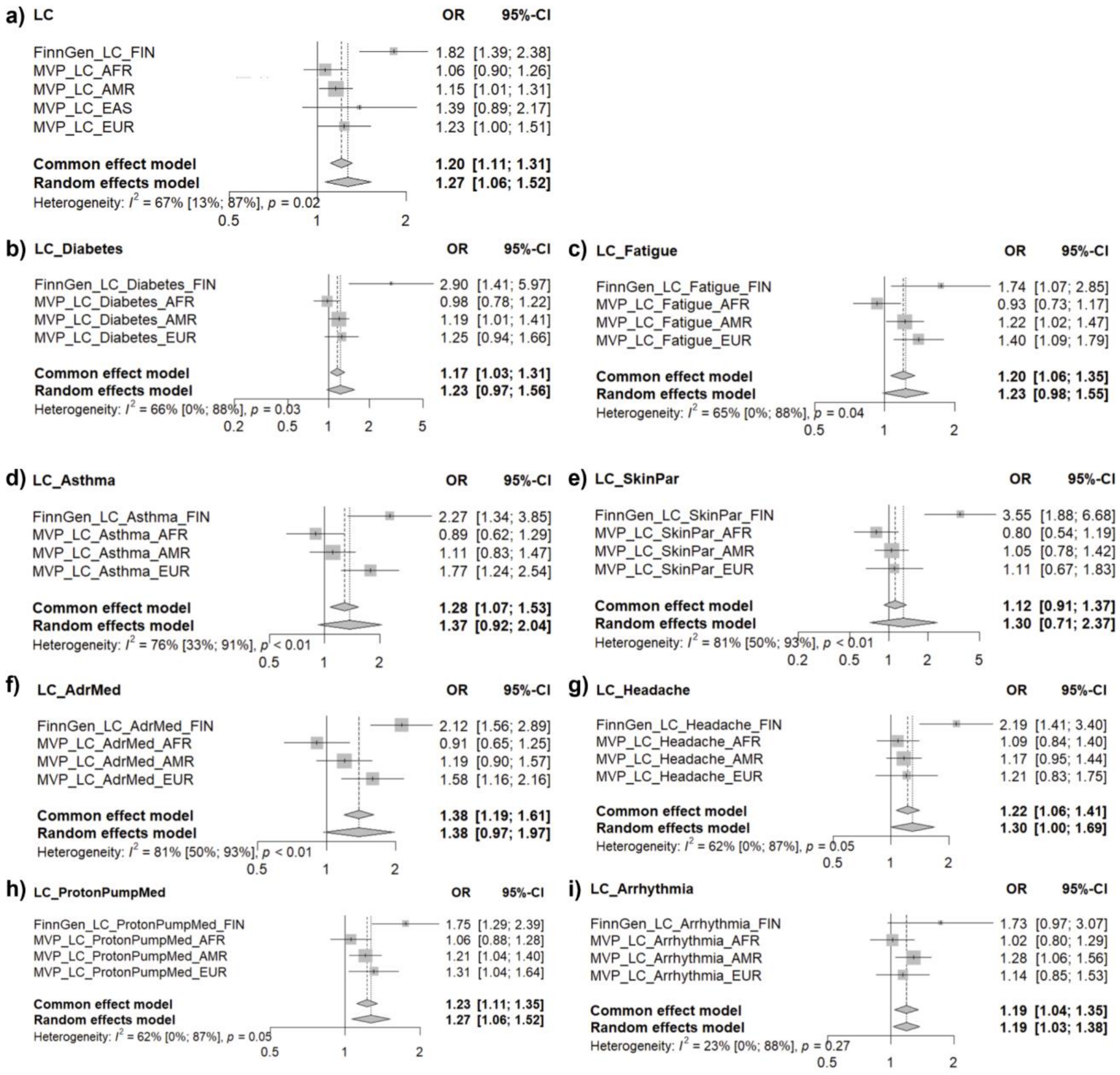

Supplementary Figure 13. *FOXP4* variant effect on subtypes of Long COVID in FinnGen and VA Million Veteran Program.

Meta-analysis of Long COVID (LC) in FinnGen[7] and VA Million Veteran Program (MVP). **a)** All Long COVID cases (ICD-10 diagnosis code: U09* [where * can be any string]). **b-i)** Long COVID diagnosis with a lifetime occurrence of **b)** diabetes (ICD-10: E10*, E11*, E12*, E13*, E14*), **c)** fatigue and malaise (ICD-10: R53*, G93.3), **d)** asthma (ICD-10: J45*), **e)** skin paraesthesia (ICD-10: R20.2), **f)** beta-adrenergic inhalants (ATC drug code: R03AC*), **g)** headache (ICD-10: R51*), **h)** proton pump inhibitors (ATC: A02BC*), or **i)** cardiac arrhythmia / abnormalities of heart beat (ICD-10: I49*, R00*).
Odds ratio (OR) with 95% confidence interval (95% CI) of risk variant rs9367106-C on Long

COVID with each symptom or medication in each ancestry separately (logistic regression using glm function in R, adjusted for age, sex and 10 principal components). Common i.e. fixed-effect and random effect meta-analysis combining ancestries run using metagen function from meta package v6.5-0 in R v4.3.0 (weight of each sample shown as box size). Sample sizes for each genetic ancestry (Finnish European (FIN), African (AFR), Admixed American (AMR), East Asian (EAS), European (EUR)) in **Supplementary Table 36**.

References for Supplementary Figures

1. Karczewski, K. J. *et al.* The mutational constraint spectrum quantified from variation in 141,456 humans. *Nature* **581**, 434–443 (2020).
2. Marcus, J. H. & Novembre, J. Visualizing the geography of genetic variants. *Bioinformatics* **33**, 594–595 (2017).
3. Chen, S. *et al.* A genome-wide mutational constraint map quantified from variation in 76,156 human genomes. 2022.03.20.485034 Preprint at https://doi.org/10.1101/2022.03.20.485034 (2022).
4. Gamazon, E. R. *et al.* Using an atlas of gene regulation across 44 human tissues to inform complex disease- and trait-associated variation. *Nat Genet* **50**, 956–967 (2018).
5. Delorey, T. M. *et al.* COVID-19 tissue atlases reveal SARS-CoV-2 pathology and cellular targets. *Nature* **595**, 107–113 (2021).
6. Kanai, M. *et al.* Meta-analysis fine-mapping is often miscalibrated at single-variant resolution. *Cell Genom* **2**, 100210 (2022).
7. Kurki, M. I. *et al.* FinnGen provides genetic insights from a well-phenotyped isolated population. *Nature* **613**, 508–518 (2023).

Supplementary Note: Methods

Long COVID Host Genetics Initiative - Inclusion & Ethics

The Long COVID Host Genetics Initiative (HGI) is a global and ongoing collaboration project to study genetic factors associated with the risk for developing long-term health problems after SARS-CoV-2 infection. The initiative is open to all studies around the world that have data to run Long COVID genome-wide association study (GWAS) using our phenotypic criteria described below. We encourage studies from all around the world and all ancestries to join this collaborative effort.

The phenotypes and research plan have been designed together by the open global working group of the Long COVID HGI. Each contributing local study has collected the data, run their GWAS, and shared their GWAS summary statistics, which have been meta-analysed together by the initiative. Participants provided informed consent to participate in each respective study, with recruitment and ethics following study-specific protocols approved by their respective Institutional Review Boards and studies performed in accordance with the Declaration of Helsinki (Details are provided in **Supplementary Table 12**). Contributing researchers from each local study have been acknowledged as co-authors.

Phenotype definitions

The current World Health Organization definition includes any symptoms that present after COVID-19 and persist for at least three months[1]. We used clinical diagnosis or self-reported Long COVID in agreement with the World health organisation guidelines following criteria "Post COVID-19 condition occurs in individuals with a history of probable or confirmed SARS CoV-2 infection, usually 3 months from the onset of COVID-19 with symptoms and that last for at least 2 months and cannot be explained by an alternative diagnosis" (https://www.who.int/publications-detail-redirect/WHO-2019-nCoV-Post_COVID-19_condition-Clinical_case_definition-2021.1).

To limit study heterogeneity, we used either self-reported or test-verified COVID-19 infection status to define study participants as Long COVID cases. Self-reported SARS-CoV-2 infection was ascertained if the participant had a documented positive SARS-CoV-2 test result (referred to as "test-verified"), or if they had self-reported suspected COVID-19 (for example, by questionnaire; referred to as "reported") or they had SARS-CoV-2 diagnosis codes in EHR. We aimed to use this broader definition of COVID-19 diagnosis, as many affected individuals may miss a documented COVID-19 diagnosis due to the delay in developing an effective test, limited testing capacity at scale, restrictions on the breadth of public testing, and inadequate record-keeping and linkage, amongst other reasons, especially in the early stages of the pandemic[2–4].

Codes to extract cases using registry or electronic health record data

In FinnGen, to include cases based on electronic health record (EHR) data, we used the international classification of diseases version 10 (ICD-10) codes U09.9 (Post COVID-19 condition, unspecified) to assign Long COVID.

In UK Biobank, we used the following codes in the primary care data (the data access was Feb 25, 2022)
TPP local codes: Y2b89, Y2b8a, Y2b87, Y2b88
SNOMED CT: 1325161000000102, 1325031000000108, 1325041000000104, 1325181000000106, 1325021000000106, 1325141000000103, 1325081000000107, 1325061000000103, 1325071000000105, 1325051000000101

We acknowledge that the phenotype definition of Long COVID both by questionnaire and by EHR data is likely to become more precise when more is learned about the disease entity.

Strict and broad phenotype definitions

We used the following criteria for assigning case control status for Long COVID aligning with the World Health Organization guidelines (Supplementary Methods). Study participants were defined as Long COVID cases if, at least three months since SARS-CoV-2 infection or COVID-19 onset, they met any of the following criteria:

1) presence of one or more self-reported COVID-19 symptoms that cannot be explained by an alternative diagnosis
2) report of ongoing significant impact on day-to-day
3) any diagnosis codes of Long COVID (e.g. post COVID-19 condition, ICD-10 code U09(.9))

Criteria 1 and 2 were applied only to questionnaire-based cohorts, whereas 3 was used in studies with electronic health records (EHR). Detailed phenotyping criteria and diagnosis codes of each study are provided in **Supplementary Table 12**.

We used two Long COVID case definitions, a strict definition requiring a test-verified SARS-CoV-2 infection and a broad definition including self-reported or clinician-diagnosed SARS-CoV-2 infection (any Long COVID).

We applied two control definitions. First, we used population controls, i.e. everybody that is not a case. Population controls were genetic-ancestry matched individuals who were not defined as Long COVID cases using the above-mentioned questionnaire or EHR-based definition. In the second analysis, we compared Long COVID cases to individuals who had had SARS-CoV-2 infection but who did not meet the criteria of Long COVID, i.e. had fully recovered within 3 months from the infection.

We used in total four different case-control definitions to generate four GWASs as below;

1) Long COVID cases after test-verified SARS-CoV-2 infection vs population controls (strict case definition vs broad control definition)
2) Long COVID cases within test-verified SARS-CoV-2 infection (strict case definition vs strict control definition)
3) Any Long COVID cases vs population controls (broad case definition vs broad control definition)
4) Long COVID cases within any SARS-CoV-2 infection (broad case definition vs strict control definition)

As all contributing studies did not have data for all of the phenotypes, each meta-analysis comprised those studies that had the phenotypes present and had run that particular GWAS and

gone through the quality control. The GWAS results using questionnaire (Q) and EHR (E) based phenotypes were combined in the meta-analysing phase. For studies where all subjects had had a test-verified SARS-CoV-2 infection and thus qualified for the strict case definition, we included the GWASs with strict cases in the meta-analyses with broad case definitions. Similarly, for studies where all control subjects had had a SARS-CoV-2 infection and thus qualified for the strict control definition, we included the GWASs with strict controls in the broad control definition meta-analyses.
Thus, the meta-analysis with broad case and control definitions (marked with ‘3’ in the list above) included data from all of our contributing studies, and the other meta-analyses from subsets of the studies. For this reason, the results of these four Long COVID meta-analyses cannot be directly compared, and the differences between them cannot be interpreted directly as caused by e.g. test-verified vs any SARS-CoV-2, or within-COVID or population-controlled analysis.

Cohort ancestry and description

24 studies from 16 countries and 6 ancestries contributed data in the GWAS meta-analyses. In Fig. 1., we display the effective sample size of each analysis which we calculated using the formula $((4 \times N_{case} \times N_{control})/(N_{case} + N_{control}))$. Per-study sample sizes across each phenotype are given in **Supplementary Table 11**. Study-specific information on participants and methods, including ethics and consent, is provided in **Supplementary Figure 2**.

Each study projected their cohort onto a multi-ethnic genetic principal component space, with pre-computed PC loadings and reference allele frequencies from unrelated samples from the 1000 Genomes Project and the Human Genome Diversity Project. PCA script internally used the PLINK2 --score function with the variance-standardise option and reference allele frequencies (--read-freq). Consequently, each cohort-specific genotype dosage matrix was mean-centred and variance-standardised with respect to reference allele frequencies, but not cohort-specific allele frequencies. We further normalised the projected PC scores by dividing the values by a square root of the number of variants used for projection to account for a subtle difference due to missing variants.

Data harmonization

To allow harmonized data across cohorts, we first compared allele frequencies to Gnomad and aligned alleles to gnomAD 3.0. For any cohorts that were not in genome build 38, we used Picard for liftover. As cohorts provided only summary statistics level information, we examined allele frequency by imputation info scores at cohort level. In addition, we examined association statistics by plotting quantile quantile plots for expected and observed P-values in each cohort. Furthermore, we plotted Manhattan plots for each cohort.

GWAS meta-analyses

We computed inverse-variance weighted meta-analysis, which is a method that summarises effect sizes across the multiple studies by computing the mean of the effect sizes weighted by the inverse variance in each individual study. We provide the code to perform the meta-analysis at LongCOVID HGI GitHub (https://github.com/long-covid-hg/META_ANALYSIS/). This meta-analysis pipeline is a modified version of the pipeline used for the main COVID HGI analysis (https://github.com/covid19-hg/META_ANALYSIS). We provide Bonferroni-adjusted threshold that accounts for multiple testing of our 4 phenotypes, albeit it might be overly conservative given that the traits we tested were all Long COVID and were correlated with each other and comprised of partially overlapping individuals. Thus, we also report loci ($P < 5\times10^{-8}$) and report the unadjusted P values for each variant. Furthermore, we investigated the heterogeneity between estimates from contributing studies at variant level using Cochran's Q-test. This is calculated for each variant as the weighted sum of squared differences between the effects sizes and their meta-analysis effect, the weights being the inverse variance of the effect size. Q is distributed as a χ2 statistic with k (number of studies) minus one degree of freedom. Furthermore, we identified a proxy variant, rs12660421 ($r^2 = 0.90$) using all individuals from the 1000 Genomes Project[5] to use in replication cohorts and downstream analyses where rs9367106 was not available.

Expression quantitative trait loci (eQTL)

For the single (Bonferroni-corrected) genome-wide significant lead variant, rs9367106, we used the GTEx portal (https://gtexportal.org/) to understand if this variant had any tissue-specific effects on gene expression. As rs9367106 was not available in the GTEx database, we first identified a proxy variant, rs12660421 ($r^2 = 0.90$) using all individuals from the 1000 Genomes Project[5], and then performed a lookup in the portal's GTEx v8 dataset[6].

Colocalization

In the coloc R package (v5.1.0.1), we performed all colocalization analyses using the *coloc.abf* function, which calculates approximate Bayes factors, with both p1 (prior probability a SNP is associated with trait 1, Long COVID) and p2 (prior probability a SNP is associated with trait 2, the named trait in the table) set to the default 1e-4 and with p12 (prior probability a SNP is associated with both traits) set to the default 1e-5. For the GTEx colocalization, we used the Ensembl Gene ID "ENSG00000137166" (corresponding to *FOXP4*) to import results from the eQTL catalogue's ftp site (ftp://ftp.ebi.ac.uk/pub/databases/spot/eQTL/imported/GTEx_V8/ge/).

Cell-type specific *FOXP4* expression

To assess the relevant cell types for *FOXP4*, we evaluated the transcriptional expression in the lung of healthy controls. We downloaded the single-cell type transcriptomic analyses, where we used all cell types in the lung (GSE13014870). We visualized RNA single cell type tissue cluster

data (transcript expression levels summarized per gene and cluster), using log10(protein-transcripts per million (pTPM)) values with “corrplot v 0.92“ R package.

RNA sequencing in the BQC19

The BQC19 (https://en.quebeccovidbiobank.ca) is a prospective cohort enrolling participants with PCR-proven SARS-CoV-2 infection and PCR-proven SARS-CoV-2 negative indivimeaduals who presented to the hospital with signs or symptoms consistent with COVID-19. Participants were recruited from eight academic hospitals in the province of Quebec, Canada. RNA is extracted from the PAXgene RNA tube collected at the same study visit and standard short-read RNA sequencing on poly-A RNAs performed on a fraction of the RNA extracted. All bulk RNA-sequencing libraries were pooled and this library pool was sequenced 100 base pair single-end on an Illumina NovaSeq 6000 to an average depth of 2,500 million reads per sample. Adapter sequences and low-quality score bases were trimmed from reads using Trim Galore (v0.6.3, Cutadapt -q 20). Trimmed reads were pseudoaligned to a custom transcriptome containing both the Homo sapiens reference transcriptome (GRCh38) and the Cal/04/09 transcriptome (downloaded from Ensembl) using the quant function in kallisto (v0.46.1). Gene-level expression estimates were calculated using the R (v4.1.2) package tximport (v1.22.0). Expression data was filtered for protein-coding genes that were sufficiently expressed across all samples (median logCPM > 1). After removing non-coding and lowly-expressed genes, normalization factors to scale the raw library sizes were calculated using calcNormFactors in edgeR (v3.36.0). The voom function in limma (v3.50.0) was used to apply the size factors, estimate the mean-variance relationship, and convert counts to logCPM values. RNA extraction facilities and sequencing batch and were regressed using the ComBat function in sva (v3.42.0).

The resultant *FOXP4* expression level was subjected to the downstream analysis. After removing the outliers (adjusted logCPM value < -2), we defined non-acute COVID-19 samples as those collected at least 31 days after onset of their symptoms from patients with positive for SARS-CoV-2 by PCR or samples collected from patients negative for SARS-CoV-2 (n = 314). Furthermore, we defined acute COVID-19 samples as those collected within -2 to 14 days from symptom onset from patients positive for SARS-CoV-2 (n = 328). *FOXP4* level was adjusted for age, sex and icu admission. P-values were obtained by logistic regression to assess the association of adjusted *FOXP4* with Long COVID.

Enhancers, transcription factor binding sites, and active chromatin regions

We performed functional annotation from ENCODE (https://www.encodeproject.org/), Regulome V2 (https://regulomedb.org/)[7], Cistrome (http://cistrome.org/)[8], and Variant annotation portals (http://www.mulinlab.org/vportal/index.html), examining methylation status, transcriptional activity and transcription factor binding at the variants part of the *FOXP4* haplotype. Furthermore, we identified and visualized methylation and active chromatin regions using the WashU Epigenome Browser[9] (ENCFF778NUQ.bam, ENCFF563OCJ.bam, FOXA1: ENCFF896BCU.bam, ENCFF631DQI.bam, GATA3: ENCFF999YEG.bam, ENCFF498PGZ, EP300:

ENCFF217XRA.bam, ENCFF983ZOH.bam) and validated the DNase and Chip sequencing peaks using Bamtools[10].

Phenome-wide association study (PheWAS)

To identify other phenotypes associated with the Long COVID lead variant rs9367106, we used the Biobank Japan PheWeb portal (https://pheweb.jp/)[11] to perform a phenome-wide association analysis, as the minor allele frequency of rs9367106 is highest in East Asia.

Mendelian Randomisation

Two-sample Mendelian randomization (MR) was employed to estimate causal associations between 38 cardiometabolic, behavioural, and psychiatric traits and Long COVID using the same approach as previously employed for determining causal associations with COVID-19 susceptibility and severity. Exposures included (**Supplementary Table 26**): Smoking initiation, Ischemic stroke, High-density lipoproteins, CRP, Diastolic blood pressure, Depression, Insomnia symptoms, Height, Coronary artery disease, Schizophrenia, Lupus, Sleep duration, ADHD, Pulse pressure, Systolic blood pressure, Alzheimer's disease, Risk tolerance, Cigarettes per day, Diabetes, Amyotrophic lateral sclerosis, Rheumatoid arthritis, Multiple sclerosis, Heart failure, Bipolar disorder, Low-density lipoproteins, Total cholesterol, Triglycerides, Chronic kidney disease, BMI, Autism spectrum disorder, Platelet count, Parkinson’s disease, Asthma, Red blood cell count, Idiopathic pulmonary fibrosis, White blood cell count, eGFR, and 25 hydroxyvitamin D. We also evaluated the causal association between COVID-19 hospitalization, COVID-19 critical illness, and SARS-CoV-2 infection and Long COVID. These exposures were selected based on their potential as COVID-19 risk factors based on their clinical correlation with disease susceptibility, severity, or mortality. For each exposure, the corresponding publication provides information on how it was measured or diagnosed, the units of measurement used, and the statistical models employed to generate variant associations. Where cross-ancestry discovery GWAS were conducted, EUR-ancestry only GWAS summary statistics were obtained and used in downstream analyses.

MR utilizes genetic variants as proxies for environmental exposures to estimate the causal link between an intermediate exposure and a disease outcome. MR can be compared to a "genetic randomized controlled trial," where risk factors or genotypes are randomly assigned from parents to offspring. This random assignment is not influenced by confounding factors that may affect both risk factors and disease and is unaffected by reverse causation. The genetic variants used in MR function as instrumental variables, provided the following assumptions are satisfied: (1) the genetic variants are known to be associated with the exposure (non-zero effect assumption); (2) the genetic variants are not associated with confounders (independence assumption); and (3) the genetic variants are not directly associated with the outcome (exclusion restriction assumption). In a two-sample MR, analyses are conducted using published genome-wide association summary statistics, with the SNP-exposure and SNP-outcome effects obtained from separate GWAS performed on each trait independently. These separate GWAS are assumed to be conducted in the same underlying population and have no sample overlap.

For each exposure, the respective discovery GWAS was used for both instrument selection and effect size determination. Independent genome-wide significant SNPs ($p < 5e-8$) were chosen as genetic instruments through LD clumping using PLINK ($r2 = 0.001$, 10-Mb clumping window, 1000 Genomes EUR LD reference panel). For genetic instruments not present in the Long COVID GWAS's, PLINK was used to find proxy variants in LD ($r2 > 0.8$). Variants without suitable proxy variants were excluded. The exposure and outcome datasets were then harmonized to ensure that a variant's effect corresponded to the same allele, inferring the positive strand based on allele frequencies for palindromic variants. Causal estimates were calculated using fixed-effect inverse variance weighted (IVW) meta-analysis as the primary analysis and weighted median estimator (WME), weighted mode-based estimator (WMBE), MR-Egger regression, and Mendelian randomization pleiotropy residual sum and outlier (MR-PRESSO) as sensitivity analyses. Although IVW offers the highest statistical power for estimating causal associations, it assumes that all variants are valid instruments and may produce biased estimates if the average pleiotropic effect deviates from zero. Sensitivity analyses provide consistent causal effect estimates even when some instrumental variables are invalid but at the expense of reduced statistical power. The global MR-PRESSO test was employed to assess heterogeneity, and the MR-Egger intercept to evaluate horizontal pleiotropy. Robust causal estimates were defined as those significant at an FDR of 5% and either (1) displayed no evidence of heterogeneity (MR-PRESSO global test $P > 0.05$) or horizontal pleiotropy (Egger intercept $P > 0.05$); or (2) in the presence of heterogeneity or horizontal pleiotropy, the WME-, WMBE-, MR-Egger-, or MR-PRESSO-corrected estimates were significant ($P < 0.05$).

Since no significant causal associations were found between Long COVID and the 38 disease, health, and neuropsychiatric phenotypes, sensitivity analyses like LHC-MR or MRLap, which account for sample overlap, were not carried out. To avoid sample overlap between exposure GWASs (here COVID-19 hospitalization and SARS-CoV-2 reported infection) and outcome GWASs (here Long COVID phenotypes), we performed meta-analyses of COVID-19 hospitalization and SARS-CoV-2 reported infection using data freeze 7 of the COVID-19 HGI by excluding studies that participated in the Long COVID (freeze 4) effort.
Leave-one-variant-out MR analysis (IVW, random effects model) was also carried out to test robustness of the causal association observed from COVID-19 hospitalization to Long COVID.

The Long COVID phenotype dataset was predominantly composed of individuals of European (EUR) ancestry. An MR analysis with European only Long COVID cohorts was run as a sensitivity analysis with COVID hospitalization as exposure and Long COVID as outcome. Furthermore, as the genetic instruments for most exposures were selected entirely from populations of EUR ancestry, we cannot comment on applicability of those findings to other ancestries. However, the findings should be generalizable across other exposure levels and timings.

Statistical analyses were performed using R v.4.0.3 (where not otherwise mentioned). Initial MR analysis was conducted using the 'TwoSampleMR' v.0.5.5 package, and sensitivity MR analyses (leave-one-variant-out and EUR only) using 'TwoSampleMR' v.0.6.8 and R v.4.3.0.

Genetic correlation

We used Linkage disequilibrium score regression (LDSC)[12] to estimate genetic correlations between the Long COVID phenotypes and a set of potential risk factors, biomarkers, and diseases that were earlier studied as part of COVID-19 susceptibility and severity[13]. In addition, we computed genetic correlation analysis with COVID-19 susceptibility and severity. We provide the sources for each GWAS summary statistics for these other traits and the association statistics for all exposure variants as part of the supplementary material (**Supplementary Table 26, Supplementary Dataset 1**, respectively).

Furthermore, we compared the differences between the observed genetic correlations of SARS-CoV-2 infection and COVID-19 severity using a *z*-score method[14].

Fine-mapping

We estimated LD between variants using 1000 Genomes reference panel as implemented in the LD link web portal[15]. Furthermore, we performed fine-mapping within the 70 kb region at chr6:41,490,001-41,560,000, the *FOXP4* locus suggested by the local LD. We utilized SLALOM for fine-mapping[16].

Bayesian clustering of effects based on linear relationships

As individuals who develop Long COVID need to have earlier COVID-19 infection, and as COVID-19 severity has been associated with Long COVID in epidemiological studies, we compared effect size estimates between Long COVID and COVID severity, and similarly, between Long COVID and SARS-CoV-2 infection. We used COVID-19 hospitalization as a proxy for severity. For this purpose, we selected those variants that were earlier classified as having effect on COVID-19 severity or susceptibility (see Supplementary Fig. 5, Supplementary Table 5, and Supplementary Note in [17]) and examined if these variants had shared effects with Long COVID. To do this, we utilized a Bayesian mixture model as implemented in the linemodels R package for comparing linear relationships (https://github.com/mjpirinen/linemodels)[18]. This method performs probabilistic clustering of variables based on their observed effect sizes on two outcomes. Instead of a direct distance comparison between the lines and the points, this method accounts for varying uncertainty of the effects on the two outcomes and for possible correlation between the effects on the two outcomes and defines Gaussian probability models surrounding the lines.
In both of our analyses, we used four models to represent the variants: (1) effects on only susceptibility or severity, (2) effects on only Long COVID, (3) effects on both outcomes with a smaller slope, and (4) effects on both outcomes with a larger slope. We allowed two separate

line models for the shared effects to model the possibility that there can be more than one relationship between the effect sizes among the variants with shared effects (P for model improvement compared to model with only one shared effect = 0.005). In both analyses, we first optimized the slope parameters of the two models representing the shared effects using function '*line.models.optimize*'. All scale parameters were fixed to 0.25 and all cor parameters were fixed to 0.995 throughout the analyses. As the result, the optimized slope parameters for the two models with shared effects were 4.92 (model 'both1') and 1.34 (model 'both2') in Infection vs. Long COVID analysis (**Fig. 5e**) and 1.69 (model 'both1') and 0.24 (model 'both2') in Hospitalization vs. Long COVID analysis (**Fig. 5f**). The posterior probabilities of each variant belonging to each of the four models were computed using the function *'line.models.with.proportions'*.

Stratified and adjusted analyses for strain, vaccination, and severity

We utilized data from FinnGen, Estonian Biobank, UK Biobank, and Mass General Brigham biobank to run adjusted and stratified analyses for COVID-19 severity and vaccination. For vaccination, we ran adjusted analysis using COVID-19 vaccine prior to SARS-CoV-2 infection as a covariate. This analysis was done in all subcohorts. In addition, we ran stratified analysis, where the cases had a SARS-CoV-2 infection prior to their first vaccination and developed Long COVID, and second where the cases had the infection after their first vaccination and then developed Long COVID.

For severity, we ran an analysis using COVID-19 hospitalization as a dichotomized covariate. This analysis was performed in all above mentioned subcohorts. In addition, we prepared the following phenotypes and ran these primarily in FinnGen[19]:

Within-COVID = only individuals that have had SARS-CoV-2 included in controls i.e. strict control condition.
COVID_Hosp = Hospitalized with SARS-CoV-2 infection.
LC_COVID_Hosp = population-controlled analysis with only Long COVID cases after hospitalized COVID-19.
LC_S_nonHospit = stratified analysis with Long COVID compared to individuals not hospitalized with COVID-19.
LC_S_CtrlNonHospit = similar analysis as previous, except hospitalized only excluded from controls, not Long COVID cases.
LC_A_COVID_Hosp = adjusted analysis with dichotomous COVID hospitalization added as a covariate.

Finally, we defined epidemic seasons by viral strain as defined by local health authorities and stratified the analysis by Wuhan (wild type), alpha, delta or omicron waves. This analysis was computed in FinnGen and Estonian Biobank.

Cox proportional hazard and recessive model

We computed a cox proportional hazard model for *FOXP4* genotypes using age, sex and first ten principal components as covariates, COVID-19 diagnosis or test date as an event date. We included data from FinnGen release 12 that had a longer follow up time till April 2023 and a larger number of Long COVID cases (N = 3,684, N population controls = 496,664).
In addition, we computed a recessive model using regenie in FinnGen. We then examined the recessive association in Estonian Biobank and in MexGene-COVID cohorts. In Estonian Biobank we observed that 27 individuals were homozygous for *FOXP4* risk allele. Out of these 27 individuals, 4 had a diagnosis of Long COVID. Furthermore, to obtain more power for this analysis we examined data from MexGene-COVID cohort, where allele frequency of the *FOXP4* risk variant is higher. Among controls 14% were homozygous for the *FOXP4* risk allele, whereas 23% of the individuals with Long COVID diagnosis were homozygous for the *FOXP4* risk allele.

References for Supplementary Methods

1. Soriano, J. B., Murthy, S., Marshall, J. C., Relan, P. & Diaz, J. V. A clinical case definition of post-COVID-19 condition by a Delphi consensus. Lancet Infect. Dis. 22, e102–e107 (2022).
2. Vandenberg, O., Martiny, D., Rochas, O., van Belkum, A. & Kozlakidis, Z. Considerations for diagnostic COVID-19 tests. Nat. Rev. Microbiol. 19, 171–183 (2021).
3. Iacobucci, G. Covid-19: Lack of capacity led to halting of community testing in March, admits deputy chief medical officer. BMJ 369, m1845 (2020).
4. Holmgren, A. J., Apathy, N. C. & Adler-Milstein, J. Barriers to hospital electronic public health reporting and implications for the COVID-19 pandemic. J. Am. Med. Inform. Assoc. JAMIA 27, 1306–1309 (2020).
5. Auton, A. et al. A global reference for human genetic variation. Nature 526, 68–74 (2015).
6. The GTEx Consortium atlas of genetic regulatory effects across human tissues. Science 369, 1318–1330 (2020).
7. Dong, S. et al. Annotating and prioritizing human non-coding variants with RegulomeDB v.2. Nat. Genet. 55, 724–726 (2023).
8. Liu, T. et al. Cistrome: an integrative platform for transcriptional regulation studies. Genome Biol. 12, R83 (2011).
9. Li, D. et al. WashU Epigenome Browser update 2022. Nucleic Acids Res. 50, W774–W781 (2022).
10. Barnett, D. W., Garrison, E. K., Quinlan, A. R., Strömberg, M. P. & Marth, G. T. BamTools: a C++ API and toolkit for analyzing and managing BAM files. Bioinforma. Oxf. Engl. 27, 1691–1692 (2011).
11. Sakaue, S. et al. A cross-population atlas of genetic associations for 220 human phenotypes. Nat. Genet. 53, 1415–1424 (2021).
12. Finucane, H. K. et al. Partitioning heritability by functional annotation using genome-wide association summary statistics. Nat. Genet. 47, 1228–1235 (2015).
13. Yang, Z. et al. Genetic Landscape of the ACE2 Coronavirus Receptor. Circulation 145, 1398–1411 (2022).

14. Zhou, T. et al. Educational attainment and drinking behaviors: Mendelian randomization study in UK Biobank. Mol. Psychiatry 26, 4355–4366 (2021).
15. Machiela, M. J. & Chanock, S. J. LDlink: a web-based application for exploring population-specific haplotype structure and linking correlated alleles of possible functional variants. Bioinformatics 31, 3555–3557 (2015).
16. Kanai, M. et al. Meta-analysis fine-mapping is often miscalibrated at single-variant resolution. Cell Genom 2, 100210 (2022).
17. Kanai, M. et al. A second update on mapping the human genetic architecture of COVID-19. Nature 621, E7–E26 (2023).
18. Pirinen, M. linemodels: clustering effects based on linear relationships. Bioinformatics 39, btad115 (2023).
19. Kurki, M. I. et al. FinnGen provides genetic insights from a well-phenotyped isolated population. Nature 613, 508–518 (2023).

Supplementary Note: Acknowledgements

We are extremely grateful to all the participants, healthcare professionals, interviewers, computer and laboratory technicians, clerical workers, research scientists, volunteers, managers, and everyone participating in making possible the collection and analysis of data sets contributing to this study. In addition, we acknowledge the following funding and research infrastructure support (full author information of all Long COVID Host Genetics Initiative contributors in **Supplementary Table 2**):

FinnGen

Hanna M. Ollila, Vilma Lammi: We are grateful for the support from the Academy of Finland (#353812), the NIH (R01AI170850), and the EU. This project has received funding from the European Union's Horizon Europe research and innovation programme under grant agreement No 101057553. This work was supported by the Swiss State Secretariat for Education, Research and Innovation (SERI) under contract number 22.00094.

Andrea Ganna: This project has received funding from the RIA HORIZON Research and Innovation Actions under GA No. 101057775. (Funded by the European Union. Views and opinions expressed are however those of the author(s) only and do not necessarily reflect those of the European Union or European Health and Digital Agency (HADEA). Neither the European Union nor the granting authority can be held responsible for them.).

FinnGen: We want to acknowledge the participants and investigators of FinnGen study. The FinnGen project is funded by two grants from Business Finland (HUS 4685/31/2016 and UH 4386/31/2016) and the following industry partners: AbbVie Inc., AstraZeneca UK Ltd, Biogen MA Inc., Bristol Myers Squibb (and Celgene Corporation & Celgene International II Sàrl), Genentech Inc., Merck Sharp & Dohme LCC, Pfizer Inc., GlaxoSmithKline Intellectual Property Development Ltd., Sanofi US Services Inc., Maze Therapeutics Inc., Janssen Biotech Inc, Novartis AG, and Boehringer Ingelheim International GmbH. Following biobanks are acknowledged for delivering biobank samples to FinnGen: Auria Biobank (www.auria.fi/biopankki), THL Biobank (www.thl.fi/biobank), Helsinki Biobank (www.helsinginbiopankki.fi), Biobank Borealis of Northern Finland (https://www.ppshp.fi/Tutkimus-ja-opetus/Biopankki/Pages/Biobank-Borealis-briefly-in-English.aspx), Finnish Clinical Biobank Tampere (www.tays.fi/en-US/Research_and_development/Finnish_Clinical_Biobank_Tampere), Biobank of Eastern Finland (www.ita-suomenbiopankki.fi/en), Central Finland Biobank (www.ksshp.fi/fi-FI/Potilaalle/Biopankki), Finnish Red Cross Blood Service Biobank (www.veripalvelu.fi/verenluovutus/biopankkitoiminta), Terveystalo Biobank (www.terveystalo.com/fi/Yritystietoa/Terveystalo-Biopankki/Biopankki/) and Arctic Biobank (https://www.oulu.fi/en/university/faculties-and-units/faculty-medicine/northern-finland-birth-cohorts-and-arctic-biobank). All Finnish Biobanks are members of BBMRI.fi infrastructure (www.bbmri.fi). Finnish Biobank Cooperative -FINBB (https://finbb.fi/) is the coordinator of BBMRI-ERIC operations in Finland. The Finnish biobank data can be accessed through the Fingenious® services (https://site.fingenious.fi/en/) managed by FINBB.

ALSPAC - Avon Longitudinal Study of Parents and Children

Ruth E. Mitchell: REM works within the MRC Integrative Epidemiology Unit at the University of Bristol, which is supported by the Medical Research Council (MC_UU_00032/01).

Alex S.F. Kwong: We are extremely grateful to all the families who took part in the ALSPAC study, the midwives for their help in recruiting them, and the whole ALSPAC team, which includes interviewers, computer and laboratory technicians, clerical workers, research scientists, volunteers, managers, receptionists and nurses. The UK Medical Research Council and Wellcome (Grant ref: 217065/Z/19/Z) and the University of Bristol provide core support for ALSPAC. GWAS data was generated by Sample Logistics and Genotyping Facilities at Wellcome Sanger Institute and LabCorp (Laboratory Corporation of America) using support from 23andMe. This publication is the work of the authors and Nicholas J. Timpson and Ruth E. Mitchell will serve as guarantors for the contents of this paper.

George Davey Smith: GDS works within the MRC Integrative Epidemiology Unit at the University of Bristol, which is supported by the Medical Research Council (MC_UU_00032/01).

Nicholas J. Timpson: NJT is a Wellcome Trust Investigator (202802/Z/16/Z), is the PI of the Avon Longitudinal Study of Parents and Children (MRC & WT 217065/Z/19/Z), is supported by the University of Bristol NIHR Biomedical Research Centre (BRC-1215-2001), the MRC Integrative Epidemiology Unit (MC_UU_00011/1) and works within the CRUK Integrative Cancer Epidemiology Programme (C18281/A29019). For the purpose of Open Access, the author has applied a CC BY public copyright licence to any Author Accepted Manuscript version arising from this submission.

BoSCO - Bonn Study of COVID Genetics

Kerstin U. Ludwig: The BoSCO study thank Julia Fazaal and Selina Rolker for support in study management and technical work.

DBDS - Danish Blood Donor Study

Ole B.V. Pedersen: The Danish analysis was funded by 101057129 — REACT — HORIZON-HLTH-2021-DISEASE-04 and DFF 0214-00127B.

DBDS Genomic Consortium: We thank the DBDS participants for their contributions. We acknowledge funding from the Novo Nordisk Foundation (grants NNF17OC0027594 and NNF14CC0001) and Sygesikring Danmark (2020-0178).

EXCEED - Extended Cohort for E-health, Environment and DNA

Chiara Batini: EXCEED has been supported by the University of Leicester, the National Institute for Health and Care Research Leicester Respiratory Biomedical Research Centre, the Wellcome Trust (WT 202849), and Medical Research Council grants G0902313 and UKRI_PC_19004, the latter through the UK Research and Innovation Industrial Strategy Challenge Fund, delivered through Health Data Research UK. EXCEED has also been supported by Cohort Access fees from studies funded by the Medical Research Council (MRC), Biotechnology and Biological Sciences Research Council, National Institute for Health and Care Research, the UK Space Agency, and GlaxoSmithKline. The views expressed are those of the author(s) and not necessarily those of the NIHR or the Department of Health and Social Care. This study used the ALICE High Performance Computing Facilities at the University of Leicester. The EXCEED study gratefully acknowledges the support of all participants and staff who have contributed to the study.

Louise V. Wain: LVW holds a GlaxoSmithKline Asthma + Lung UK Chair in Respiratory Research (C17-1). This research was partially supported by the National Institute for Health Research (NIHR) Leicester Biomedical Research Centre; the views expressed are those of the author(s) and not necessarily those of the National Health Service, the NIHR, or the Department of Health.

Catherine John: C.J. was supported by a Medical Research Council Clinical Research Training Fellowship (MR/P00167X/1).

Anna L. Guyatt: A.L.G. was funded by internal fellowships at the University of Leicester from the Wellcome Trust Institutional Strategic Support Fund (204801/Z/16/Z) and the BHF Accelerator Award (AA/18/3/34220).

GCAT - Genomes for Life (COVICAT study - Cohort COVID in Catalonia)

Rafael de Cid: This study makes use of data generated by the GCAT - Genomes for Life and COVICAT study - Cohort COVID in Catalonia. GCAT is a cohort study of the Genomes of Catalonia, Fundacio IGTP. IGTP is part of the CERCA Program / Generalitat de Catalunya. GCAT have additional support by Spanish National Grant PI18/01512, TED2021-130626B-I00, La MaratoTV3 167/C/2021 and European Union under grant agreement no. 101046314 (END-VOC), and with partial support of MENARINI. Additional data included in this study was obtained in part by the COVICAT Study Group (Cohort Covid de Catalunya) supported by ISGlobal and IGTP, EIT COVID-19 Rapid Response activity 20873A and SR20-01024 La Caixa Foundation. This study was carried out using anonymized data provided by the Catalan Agency for Quality and Health Assessment, within the framework of the PADRIS Program. The authors of this study would like to acknowledge all GCAT project investigators who contributed to the generation of the GCAT data. A full list of the investigators is available from www.genomesforlife.com/. We thank the Blood and Tissue Bank from Catalonia (BST) and all the GCAT volunteers that participated in the study.

Beatriz Cortés: Beatriz Cortés is supported by national grants PI18/01512.

Xavier Farré: Xavier Farré is supported by TED2021-130626B-I00.

GENCOVID - Genetic Bases of COVID19 Clinical Variability

GEN-COVID Multicenter Study: Project Tuscany Health Ecosystem spoke 7 translational medicine for rare, oncological and infectious diseases financed by European Union- Next generation EU, Mission 4 Component 2 Inv. 15 CUP:2266-2022-RA-PROFCMUR_PNRR_PC_THE_AFFSPOK; and INTERVENE EU H2020-SC1-FA-DTS-2018–2020, International consortium for integrative genomics prediction - Grant Agreement No. 101016775.

GOLD - Genetics Of Long Covid

Patrick J. Short: We would like to acknowledge the participants in the Genetics of Long COVID study for their time and effort to participate and help to advance our understanding of genetic factors influencing Long COVID risk and severity. We would also like to acknowledge the funders, InnovateUK (grant numbers 87774 and 60502).

Helix - Helix Exome+ and Healthy Nevada Project COVID-19 Phenotypes

Elizabeth T. Cirulli: Funding was provided to Desert Research Institute (DRI) by the Nevada Governor's Office of Economic Development. Funding was provided to the Renown Institute for Health Innovation by Renown Health and the Renown Health Foundation.

Ioannina - Covid-19 Ioannina Biobank

Konstantinos K. Tsilidis: We want to acknowledge the participants and investigators of the Epirus Health Study (EHS). The EHS project is co-financed by the European Regional Development Fund of the European Union and Greek national funds through 1. the Operational Program Competitiveness, Entrepreneurship and Innovation (EPAnEK), NSRF 2014-2020 (Project code MIS: ΟΠΣ 5047228), and 2. the Operational Programme Epirus 2014–2020 of the Prefecture of Epirus (Project code MIS: ΗΠ1ΑΒ-0028180). The work in the Epirus Health Study

was also supported by the National Public Investment Program of the Ministry of Development and Investment/General Secretariat for Research and Technology, in the framework of the Flagship Initiative to address SARS-CoV-2 (2020ΣE01300001).

JapanTaskForce - Japan COVID-19 Task Force

Makoto Ishii: This research was supported by AMED under Grant Number JP22wm0325031.

Lifelines

C.A. Robert Warmerdam: We wish to acknowledge the efforts of the Lifelines Cohort Study, the contributing research centers delivering data to Lifelines, all the study participants, and the contributions of the investigators to this study: Raul Aguirre-Gamboa, Patrick Deelen, Lude Franke, Jan A Kuivenhoven, Esteban A Lopera Maya, Ilja M Nolte, Serena Sanna, Harold Snieder, Morris A Swertz, Judith M Vonk and Cisca Wijmenga. We wish to acknowledge the efforts of the Lifelines Corona Research Initiative and the following initiative participants: H. Marike Boezen, Jochen O. Mierau, Lude H. Franke, Jackie Dekens, Patrick Deelen, Pauline Lanting, Judith M. Vonk, Ilja Nolte, Anil P.S. Ori, Annique Claringbould, Floranne Boulogne, Marjolein X.L. Dijkema, Henry H. Wiersma, C.A. Robert Warmerdam, Soesma A. Jankipersadsing and Irene V. van Blokland.

Lude H. Franke: Lude Franke and Robert Warmerdam are supported by grants from the Dutch Research Council (ZonMW-VICI 09150182010019 to L.F. ZonMW LongCOVID grant 10430302110002), European Union’s Horizon Europe Research and Innovation Program grant 101057553 (LongCovid) and through a Senior Investigator Grant from the Oncode Institute.

MexGen-COVID - MexGen-COVID Initiative

Teresa Tusié-Luna: Financial support grants CONACYT 312688 and PAPIIT/UNAM IG20042.

Alicia Huerta-Chagoya: A.H.-C. is supported by the American Diabetes Association grant 11-23-PDF-35.

SweCovid / COMRI - Follow-UP study of patients with critical COVID-19 / COVID-19 Cohort Study of the University Hospital of the Technical University Munich (Muenchen rechts der Isar)

Eva C. Schulte: COMRI was financed through in house institutional funding from the Technical University of Munich. ECS was supported by the Munich Clinician Scientist Programm and the German Research Foundation (SCHU 2419/2-1).

Michael Marks-Hultström: The SweCOVID study thank research nurses Joanna Wessbergh and Elin Söderman for their expertise in compiling patient data, biobank assistants Philip Karlsson, Erik Danielsson, Labolina Spång and Amanda Svensson for sample collection and patient inclusion. The SweCOVID was funded by the SciLifeLab/Knut and Alice Wallenberg national COVID-19 research program (M.H.: KAW 2020.0182, KAW 2020.0241), the Swedish Heart-Lung Foundation (M.H.: 20210089, 20190639, 20190637), the Swedish Research Council (R.F.: 2014-02569, 2014-07606, and H.Z.: 2021-03050), Swedish Society of Medicine (M.H.:SLS-938101), the and Swedish Kidney Foundation (R.F.: F2020-0054).

TiKoCo - Tirschenreuth Study

Ralf Wagner: This work was supported by the Bavarian States Ministry of Science and Arts (StMWK; grant to Ralf Wagner and Klaus Überla) as well as by the National Research Network of the University Medicine (NUM; applied surveillance and testing; B-FAST) to Ralf Wagner and Klaus Überla. The funders had no role in study design, data collection and analysis, decision to publish, or preparation of the manuscript. We are particularly grateful to all the study participants. We would like to thank the office staff and numerous students of the University of Regensburg and the University Erlangen as well as the employees of the Bavarian Red Cross,

the members of the civil protection, the county office, and the public health office of county Tirschenreuth, respectively, for their tremendous support. We are also grateful to Christine Wolff from wECARE and Jakob Niggel from MaganaMed for providing questionnaires and the database for the study.

Iris M. Heid: This work was supported by the Bavarian States Ministry of Science and Arts (StMWK; grant to Ralf Wagner and Klaus Überla) as well as by the National Research Network of the University Medicine (NUM; applied surveillance and testing; B-FAST) to Ralf Wagner and Klaus Überla. The funders had no role in study design, data collection and analysis, decision to publish, or preparation of the manuscript. We are particularly grateful to all the study participants. We would like to thank the office staff and numerous students of the University of Regensburg and the University Erlangen as well as the employees of the Bavarian Red Cross, the members of the civil protection, the county office, and the public health office of county Tirschenreuth, respectively, for their tremendous support. We are also grateful to Christine Wolff from wECARE and Jakob Niggel from MaganaMed for providing questionnaires and the database for the study.

TwinsUK

Massimo Mangino: TwinsUK is funded by the Wellcome Trust, Medical Research Council, Versus Arthritis, European Union Horizon 2020, Chronic Disease Research Foundation (CDRF), Zoe Ltd, the National Institute for Health and Care Research (NIHR) Clinical Research Network (CRN) and Biomedical Research Centre based at Guy’s and St Thomas’ NHS Foundation Trust in partnership with King’s College London.

UnderstandingSociety - Understanding Society: UK Household Longitudinal Study

Meena Kumari: The UK Household Longitudinal Study (Understanding Society) is led by the Institute for Social and Economic Research at the University of Essex. The UK Household Longitudinal Study is funded by the Economic and Social Research Council (ESRC: ES/H029745/1). The survey was conducted by Nat Cen and the genome-wide scan data were analyzed and deposited by the Wellcome Trust Sanger Institute (WT098051). The Understanding Society COVID-19 study is funded by the ESRC (ES/K005146/1) and the Health Foundation (2076161). Fieldwork for the survey is carried out by Ipsos MORI and Kantar. AD is partially funded by the ESRC (ES/S012486/1). BH is an ESRC/Biotechnology & Biological Sciences Research Council funded Soc-B Ph.D student (2604276). Information on how to access the data can be found on the Understanding Society website https://www.understandingsociety.ac.uk/.

C19-GenoNet - COVID-19 Genomics Network

Ricardo A. Verdugo and C19-GenoNet team: We want to acknowledge the participants and investigators of C19-GenoNet study and the University of Chile's Biobank (BTUCH). The C19-GenoNet project is funded by grants ANID COVID0961, COVID0789, COVID1005, ESR UTA2295 and ACT210085.

CHIRP - COVID-19 Host Immune Response Pathogenesis Study

Sulggi A. Lee: We want to acknowledge the participants of the CHIRP study. This work was supported by awards 2160 from Fast Grants.

EstBB - Estonian Biobank

Erik Abner: The work of E.A. was funded by the European Union through Horizon 2020 and Horizon Europe research and innovation programs under grants no. 894987, 101137201 and 101137154, and Estonian Research Council Grant PRG1291.

Estonian Biobank Research Team: We want to acknowledge the participants of the Estonian Biobank for their contributions. The Estonian Biobank analyses were partially carried in the High Performance Computing Center, University of Tartu.

FOGS - Fondazione COVID-19 Genomic Study

Luca V.C. Valenti: Fondazione IRCCS Ca' Granda 5permille, "Fondazione COVID-19 Genomic Study (FOGS)".

GENCOV - GENCOV Study

GENCOV Study: GENCOV: This work was supported by the Canadian Institutes of Health Research (Funding Reference Number VR4-172753).

LGDB - Genome Database of the Latvian Population

Laura Ansone: LGDB: European Regional Development Fund (ERDF), Measure 1.1.1.1 “Support for applied research”, project „Identification of molecular determinants associated with the risk for various COVID-19 long term effects: a comprehensive cohort-based study in Latvian population. (POST-COVID-TRACK)” financially supported sample analysis for Latvian cohort.

MGB – MassGeneralBrigham

Richa Saxena: Richa Saxena and Hanna M. Ollila are partially supported by NIH R01AI170850.

MVP - VA Million Veteran Program

Kelly Cho, Alexis A. Rodriguez, Tianxi Cai, Daniel C. Posner, Shiuh-Wen Luoh, Ravi K. Madduri, Sudha K. Iyengar: This research is supported by the Million Veteran Program (awards MVP#000 and MVP#067) and the Centralized Interactive Phenomics Resource (CIPHER) Program funded by the Office of Research and Development, Veterans Health Administration. This research used resources of the Knowledge Discovery Infrastructure at Oak Ridge National Laboratory, which is supported by the Office of Science of the US Department of Energy under Contract No. DE-AC05-00OR22725. This publication does not represent the views of the Department of Veteran Affairs or the United States Government.

VA Million Veteran Program: We thank the Veterans, staff, and investigators of VA Million Veteran Program. The VA Million Veteran Program is funded by the Office of Research and Development, Veterans Health Administration.

PHOSP-COVID - Post-hospitalisation COVID-19 study

Beatriz Guillen-Guio: BGG is supported by Wellcome Trust grant 221680/Z/20/Z. For the purpose of open access, the author has applied a CC BY public copyright license to any Author Accepted Manuscript version arising from this submission.

PHOSP-COVID Collaborative Group: PHOSP-COVID is jointly funded by a grant from the MRC-UK Research and Innovation and the Department of Health and Social Care through the National Institute for Health Research (NIHR) rapid response panel to tackle COVID-19 (grant references: MR/V027859/1 and COV0319). The views expressed in the publication are those of the author(s) and not necessarily those of the National Health Service (NHS), the NIHR or the Department of Health and Social Care. This study would not be possible without all the participants who have given their time and support. We thank all the participants and their families. We thank the many research administrators, health-care and social-care professionals who contributed to setting up and delivering the study at all of the 65 NHS trusts/Health boards and 25 research institutions across the UK, as well as all the supporting staff at the NIHR Clinical Research Network, Health Research Authority, Research Ethics Committee, Department of Health and Social Care, Public Health Scotland, and Public Health England, and support from

the ISARIC Coronavirus Clinical Characterisation Consortium. We thank Kate Holmes at the NIHR Office for Clinical Research Infrastructure (NOCRI) for her support in coordinating the charities group. The PHOSP-COVID industry framework was formed to provide advice and support in commercial discussions, and we thank the Association of the British Pharmaceutical Industry as well NOCRI for coordinating this. We are very grateful to all the charities that have provided insight to the study: Action Pulmonary Fibrosis, Alzheimer's Research UK, Asthma + Lung UK, British Heart Foundation, Diabetes UK, Cystic Fibrosis Trust, Kidney Research UK, MQ Mental Health, Muscular Dystrophy UK, Stroke Association Blood Cancer UK, McPin Foundations, and Versus Arthritis. We thank the NIHR Leicester Biomedical Research Centre patient and public involvement group and Long Covid Support.

Other Long COVID Host Genetics Initiative contributors

Brian E. Fulton-Howard: This work was supported in part through the computational resources and staff expertise provided by Scientific Computing at the Icahn School of Medicine at Mount Sinai and supported by the Clinical and Translational Science Awards (CTSA) grant UL1TR004419 from the National Center for Advancing Translational Sciences. Research reported in this paper was supported by the Office of Research Infrastructure of the National Institutes of Health under award number S10OD026880 and S10OD030463. The content is solely the responsibility of the authors and does not necessarily represent the official views of the National Institutes of Health.

Pasquale Striano: Research supported by PNRR-MUR-M4C2 PE0000006 Research Program "MNESYS"—A multiscale integrated approach to the study of the nervous system in health and disease. IRCCS 'G. Gaslini' is a member of ERN-Epicare.
