# Supplementary material for: Genome-wide association study of long COVID
Source: Nat Genet. 2025 May 21;57(6):1402–17. doi: 10.1038/s41588-025-02100-w (PMC12165857; doi:10.1038/s41588-025-02100-w)
Supplement: Supplementary file 2 — Reporting Summary [file 41588_2025_2100_MOESM2_ESM.pdf]

nature portfolio

Corresponding author(s): Hanna M. Ollila, Hugo Zeberg

Last updated by author(s): 01/14/2025

Reporting Summary

Nature Portfolio wishes to improve the reproducibility of the work that we publish. This form provides structure for consistency and transparency in reporting. For further information on Nature Portfolio policies, see our Editorial Policies and the Editorial Policy Checklist.

Statistics

For all statistical analyses, confirm that the following items are present in the figure legend, table legend, main text, or Methods section.

n/a	Confirmed	
☐	☒	The exact sample size (*n*) for each experimental group/condition, given as a discrete number and unit of measurement
☐	☒	A statement on whether measurements were taken from distinct samples or whether the same sample was measured repeatedly
☐	☒	The statistical test(s) used AND whether they are one- or two-sided<br>*Only common tests should be described solely by name; describe more complex techniques in the Methods section.*
☐	☒	A description of all covariates tested
☐	☒	A description of any assumptions or corrections, such as tests of normality and adjustment for multiple comparisons
☐	☒	A full description of the statistical parameters including central tendency (e.g. means) or other basic estimates (e.g. regression coefficient) AND variation (e.g. standard deviation) or associated estimates of uncertainty (e.g. confidence intervals)
☐	☒	For null hypothesis testing, the test statistic (e.g. *F*, *t*, *r*) with confidence intervals, effect sizes, degrees of freedom and *P* value noted<br>*Give P values as exact values whenever suitable.*
☐	☒	For Bayesian analysis, information on the choice of priors and Markov chain Monte Carlo settings
☐	☒	For hierarchical and complex designs, identification of the appropriate level for tests and full reporting of outcomes
☐	☒	Estimates of effect sizes (e.g. Cohen's *d*, Pearson's *r*), indicating how they were calculated

Our web collection on statistics for biologists contains articles on many of the points above.

Software and code

Policy information about availability of computer code

Data collection	Provided in the manuscript Methods section, Supplementary Table 12, and Supplementary Note.<br>Instructions and example code for phenotyping, sample collection, genotyping, genotype and sample quality control, imputation, and association analyses shared in our central analysis plan.
Data analysis	Provided in the manuscript Methods section, Supplementary Table 12, Supplementary Note, and in the Code Availability statement.<br>Instructions and example code for phenotyping, sample collection, genotyping, genotype and sample quality control, imputation, and association analyses are shared in our central analysis plan (https://github.com/long-covid-hg/LongCovidTools/blob/main/COVID19HostGenetics_AnalysisPlan_LongCOVID_v1.docx, https://github.com/long-covid-hg/LongCovidTools/blob/main/PhenotypeDefinitions_LongCOVID_v1.docx). Furthermore, we have used GitHub public repositories for providing code for GWAS summary statistics lift-over and meta-analyses (https://github.com/long-covid-hg/META_ANALYSIS, modified from the previously published COVID-19 HGI pipeline), for PCA projecting and plotting (https://github.com/long-covid-hg/pca_projection), and for Mendelian randomization and genetic correlation (https://github.com/marcoralab/MRcovid). Code used for fine-mapping (https://github.com/mkanai/slalom) and Bayesian clustering of effects based on linear relationships (https://github.com/mjpirinen/linemodels) is also publicly available and has been previously published.

For manuscripts utilizing custom algorithms or software that are central to the research but not yet described in published literature, software must be made available to editors and reviewers. We strongly encourage code deposition in a community repository (e.g. GitHub). See the Nature Portfolio guidelines for submitting code & software for further information.

Data

Policy information about availability of data

All manuscripts must include a data availability statement. This statement should provide the following information, where applicable:

- Accession codes, unique identifiers, or web links for publicly available datasets
- A description of any restrictions on data availability
- For clinical datasets or third party data, please ensure that the statement adheres to our policy

Data availability (as provided in the manuscript)
We have made the results of these GWAS meta-analyses publicly available for variants passing post-meta-analysis filtering for minor allele frequency >=1% and effective sample size >1/3 of the maximum effective sample size for each meta-analysis. The results from the four meta-analyses have been deposited to GWAS Catalog and LocusZoom, where the associations can be visually explored and the summary statistics exported for further scientific discovery.
Strict case definition (Long COVID after test-verified SARS-CoV-2 infection) vs broad control definition (population control):
https://www.ebi.ac.uk/gwas/studies/GCST90454540
https://my.locuszoom.org/gwas/192226/
Broad case definition (Long COVID after any SARS-CoV-2 infection) vs broad control definition:
https://www.ebi.ac.uk/gwas/studies/GCST90454541
https://my.locuszoom.org/gwas/826733/
Strict case definition vs strict control definition (individuals that had SARS-CoV-2 but did not develop Long COVID):
https://www.ebi.ac.uk/gwas/studies/GCST90454542https://my.locuszoom.org/gwas/793752/
Broad case definition vs strict control definition:
https://www.ebi.ac.uk/gwas/studies/GCST90454543https://my.locuszoom.org/gwas/91854/

Research involving human participants, their data, or biological material

Policy information about studies with human participants or human data. See also policy information about sex, gender (identity/presentation), and sexual orientation and race, ethnicity and racism.

Reporting on sex and gender	All GWASs were performed adjusting for sex. Sex-stratified analyses can be performed in future data freezes as the sample sizes grow.
Reporting on race, ethnicity, or other socially relevant groupings	Genetic ancestry was assessed in each contributing study by principal component projection to ensure robustness of our genetic association analyses. Each study ran GWAS within-ancestry, and our multi-ancestry meta-analyses combined all studies regardless of ancestry. More info provided in the Methods and Supplementary Methods.
Population characteristics	Detailed information on the recruitment of study participants, phenotyping using diagnoses from electronic health records or questionnaire information on COVID symptoms and recovery, genetic ancestry, genotyping etc. is provided by each contributing study in the Supplementary Table 12.
Recruitment	Each of the 24 initially contributing studies and 9 replication studies recruited their participants independently. Some of the studies (such as FinnGen and UK Biobank) were larger biobank-type data sets, whereas others were smaller clinical cohorts. Please see more detailed information in the Supplementary Table 12.
Ethics oversight	Participants provided written informed consent to participate in each respective study, with recruitment and ethics following study-specific protocols approved by their respective Institutional Review Boards and studies performed in accordance with the Declaration of Helsinki. Details are provided in Supplementary Table 12 where we have now added the replication cohorts.

Note that full information on the approval of the study protocol must also be provided in the manuscript.

Field-specific reporting

Please select the one below that is the best fit for your research. If you are not sure, read the appropriate sections before making your selection.

☒ Life sciences ☐ Behavioural & social sciences ☐ Ecological, evolutionary & environmental sciences

For a reference copy of the document with all sections, see nature.com/documents/nr-reporting-summary-flat.pdf

Life sciences study design

All studies must disclose on these points even when the disclosure is negative.

Sample size	The Long COVID Host Genetics Initiative (HGI) is a global and ongoing collaboration project to study genetic factors associated with the risk for developing long-term health problems after SARS-CoV-2 infection. The initiative is open to all studies around the world that have data to run Long COVID genome-wide association study (GWAS). We have meta-analysed all Long COVID GWAS that contributing studies ran and shared to us. A total of 24 studies contributed to the analysis, with a total sample size of 6,450 Long COVID cases with 46,208 COVID-19 positive controls and 1,093,955 population controls from 6 ancestries. The finding was replicated in an independent dataset of nine additional cohorts with 9,500 Long COVID cases and 798,835 population controls.

	To maximize statistical power for detecting genetic variants associated to Long COVID, we utilized data from as many cohorts as possible with information of Long COVID and study participants without Long COVID. Moreover, to ensure reproducibility, we examined the robustness and replication of the signal across nine independent cohorts that joined the Long COVID Host Genetics Initiative after the data freeze 4 where the initial association was discovered.
Data exclusions	Genetic variants with allele frequency <0.1% or imputation INFO score <0.6 were excluded from the GWAS meta-analyses. Study-specific information on data collection and analysis is provided in the Supplementary Table 12.
Replication	The association in FOXP4 locus was replicated using an independent dataset with nine additional cohorts with 9,500 Long COVID cases and 798,835 controls.
Randomization	The phenotype definitions were designed by our global Long COVID Host Genetics Initiative working group based on clinical information on Long COVID symptoms. Each study then defined the case and control groups based on observational data (either electronic health record diagnosis data, or questionnaire information on symptoms and recovery) within their data set. Randomization does not apply to this study design.
Blinding	Our study was not a controlled trial but a genome-wide association study (GWAS) using genotypic information combined to questionnaire and electronic health record data to define case and control groups, and thus blinding and randomization do not apply.

Reporting for specific materials, systems and methods

We require information from authors about some types of materials, experimental systems and methods used in many studies. Here, indicate whether each material, system or method listed is relevant to your study. If you are not sure if a list item applies to your research, read the appropriate section before selecting a response.

Materials & experimental systems

n/a	Involved in the study	
☒	☐	Antibodies
☒	☐	Eukaryotic cell lines
☒	☐	Palaeontology and archaeology
☒	☐	Animals and other organisms
☒	☐	Clinical data
☒	☐	Dual use research of concern
☒	☐	Plants

Methods

n/a	Involved in the study	
☒	☐	ChIP-seq
☒	☐	Flow cytometry
☒	☐	MRI-based neuroimaging
